# Induced pluripotent stem-cell-derived corneal epithelium for transplant surgery: a single-arm, open-label, first-in-human interventional study in Japan

**DOI:** 10.1016/S0140-6736(24)01764-1

**Published:** 2024-11-16

**Authors:** Takeshi Soma, Yoshinori Oie, Hiroshi Takayanagi, Shoko Matsubara, Tomomi Yamada, Masaki Nomura, Yu Yoshinaga, Kazuichi Maruyama, Atsushi Watanabe, Kayo Takashima, Zaixing Mao, Andrew J Quantock, Ryuhei Hayashi, Kohji Nishida

**Affiliations:** a Department of Ophthalmology, Graduate School of Medicine, Osaka University, Suita, Osaka, Japan; b Department of Vision Informatics, Graduate School of Medicine, Osaka University, Suita, Osaka, Japan; c Laboratory of Stem Cells and Applied Medicine, Graduate School of Medicine, Osaka University, Suita, Osaka, Japan; d Department of Medical Innovation, Osaka University Hospital, Suita, Osaka, Japan; e Facility for iPS Cell Therapy, CiRA Foundation, Kyoto, Japan; f Integrated Frontier Research for Medical Science Division, Institute for Open and Transdisciplinary Research Initiatives, Osaka University, Suita, Osaka, Japan; g Uehiro Research Division for iPS Cell Ethics, Center for iPS Cell Research and Application, Kyoto University, Kyoto, Japan; h R&D Division, Topcon Corporation, Tokyo, Japan; i The School of Optometry and Vision Sciences, Cardiff University, Cardiff, UK; j Premium Research Institute for Human Metaverse Medicine, Osaka University, Suita, Osaka, Japan

## Abstract

**Background:**

The loss of corneal epithelial stem cells from the limbus at the edge of the cornea has severe consequences for vision, with the pathological manifestations of a limbal stem-cell deficiency (LSCD) difficult to treat. Here, to the best of our knowledge, we report the world's first use of corneal epithelial cell sheets derived from human induced pluripotent stem cells (iPSCs) to treat LSCD.

**Methods:**

This non-randomised, single-arm, clinical study involved four eyes of four patients with LSCD at the Department of Ophthalmology, Osaka University Hospital. They comprised a woman aged 44 years with idiopathic LSCD (patient 1), a man aged 66 years with ocular mucous membrane pemphigoid (patient 2), a man aged 72 years with idiopathic LSCD (patient 3), and a woman aged 39 years with toxic epidermal necrosis (patient 4). Allogeneic human iPSC-derived corneal epithelial cell sheets (iCEPSs) were transplanted onto affected eyes. This was done sequentially in two sets of HLA-mismatched surgeries, with patients 1 and 2 receiving low-dose cyclosporin and patients 3 and 4 not. The primary outcome measure was safety, ascertained by adverse events. These were monitored continuously throughout the 52-week follow-up period, and during an additional 1-year safety monitoring period. Secondary outcomes, reflective of efficacy, were also recorded. This study is registered with UMIN, UMIN000036539 and is complete.

**Findings:**

Patients were enrolled between June 17, 2019 and Nov 16, 2020. We had 26 adverse events during the 52-week follow-up period (consisting of 18 mild and one moderate event in treated eyes, and seven mild non-ocular events), with nine recorded in the additional 1-year safety monitoring period. No serious adverse events, such as tumourigenesis or clinical rejection, occurred during the whole 2-year observational period. At 52 weeks, secondary measures of efficacy showed that the disease stage had improved, corrected distance visual acuity was enhanced, and corneal opacification had diminished in all treated eyes. Corneal epithelial defects, subjective symptoms, quality-of-life questionnaire scores and corneal neovascularisation mostly improved or were unchanged. Overall, the beneficial efficacy outcomes achieved for patients 1 and 2 were better than those achieved for patients 3 and 4.

**Interpretation:**

iCEPS transplantation for LSCD was found to be safe throughout the study period. A larger clinical trial is planned to further investigate the efficacy of the procedure.

**Funding:**

The Japan Agency for Medical Research and Development, the Ministry of Education, Culture, Sports, Science, and Technology—Japan, and the UK Biotechnology and Biological Sciences Research Council.

## Introduction

The cornea of the eye is overlaid by a stratified epithelium that is essential for vision. At the limbus—an anatomical zone situated at the edge of the cornea where it adjoins the sclera (the white of the eye)—the corneal epithelium contains a reservoir of stem cells, which are located basally.^1^ These cells proliferate to provide a continuous supply of epithelial cells to the central cornea, maintaining its healthy state. Corneal limbal epithelial stem cells are highly proliferative, express p63 transcription factor, and exhibit holoclone-forming capabilities.^2^ In their absence, or when insufficient numbers are present, patients suffer from a condition known as a limbal stem-cell deficiency (LSCD).^3^ A unilateral LSCD is often associated with acquired non-immune-mediated aetiologies such as trauma to the eye caused by thermal or chemical burns. Bilateral LSCDs can be caused by acquired primary immune-mediated aetiologies (for example, Stevens-Johnson syndrome or ocular mucous membrane pemphigoid) or idiopathic or hereditary disease such as congenital aniridia.^3^ Whatever its origin, an LSCD typically leads to the enveloping of the corneal surface by fibrotic conjunctival tissue and a consequent loss of vision.

The management of an LSCD necessitates the optimisation of the ocular surface and subsequent surgical removal of conjunctival scar tissue from the corneal surface, followed by a graft of functional corneal epithelial tissue.^4^ The choice of graft material depends on the type of disease. For patients with a unilateral LSCD, autologous transplant procedures should initially be considered because they tend to engender better long-term graft survival. Such surgeries include keratolimbal autografts,^5^ autologous cultivated limbal epithelial cell transplantation,^6^, ^7^, ^8^ and autologous simple limbal transplantation,^9^ in which tissue biopsies are obtained from the patient's unaffected eye. If a patient suffers from a bilateral LSCD, however, no autologous ocular tissue is available, so candidate procedures include cadaveric limbal stem-cell transplantation,^10^ allogeneic cultivated limbal epithelial cell transplantation (including in situations in which the biopsy can be obtained from a living related donor),^11^ allogeneic cultivated limbal epithelial cell transplantation,^12^ and autologous cultivated oral mucosal epithelial cell sheet transplantation.^13^, ^14^ Despite the value of these treatments, the autologous and allogeneic approaches both come with drawbacks. These include the requirement for a biopsy of healthy limbus or oral mucosal epithelial tissue for autologous procedures, with the variability of autologous cell sources and individual fabrication regimes contributing to a sometimes uncertain outcome.^15^ In addition, postoperative neovascularisation following cultivated oral mucosal epithelial cell sheet transplantation is inevitable, variable in its severity, and very difficult to manage.^13^, ^14^ Allogeneic therapies are accompanied by problems that include the risk of immunological rejction.^10^

**Research in context**

**Evidence before this study**

We assessed the scientific literature for reports of patients with a limbal stem cell deficiency (LSCD) in which induced pluripotent stem cells (iPSCs) were used. A search of PubMed from the database's inception to April 4, 2024 was done with the terms “limbal stem cell deficiency” AND “induced pluripotent stem cells” with no language restriction. This identified 22 articles, the majority of which were review articles. No clinical studies where iPSC-related cells were applied to patients with an LSCD were found. The choice of surgery for LSCD depends on whether or not one or both eyes are affected. For patients with unilateral LSCD, autologous transplant procedures should be considered as the first surgical option because they offer better long-term graft survival potential and avoid the risks related to systemic immunosuppression that is required following allogeneic transplant surgery.

**Added value of this study**

To our knowledge, this is the first use of iPSC-derived corneal epithelial cells in transplant surgery. The procedure, grafting iPSC-derived corneal epithelial cell sheets (iCEPS) onto the ocular surface after the removal of fibrotic tissue that envelops the ocular surface, was successful and well-tolerated 2 years postoperatively in all four operated eyes. We found no safety issues (eg, clinical immunological rejection or tumour formation) throughout the whole 2-year observational period in any of the patients. At the end of the initial 52-week follow-up period, all four eyes also showed positive results clinically, with an improvement in the clinical stage of the disease, better visual acuity, and diminished corneal opacification. The outcomes were achieved without HLA matching or the use of immunosuppressive agents, apart from corticosteroids. This can probably be explained by a relatively low expression of HLA class I and II, and an absence of immunocompetent cells, such as Langerhans cell, from iCEPS.

**Implications of all the available evidence**

We describe a series of first-in-human surgeries that use human iPSC-derived epithelial cell sheets to repair the corneas of patients with visual impairment because of an LSCD.

**Figure 1:**
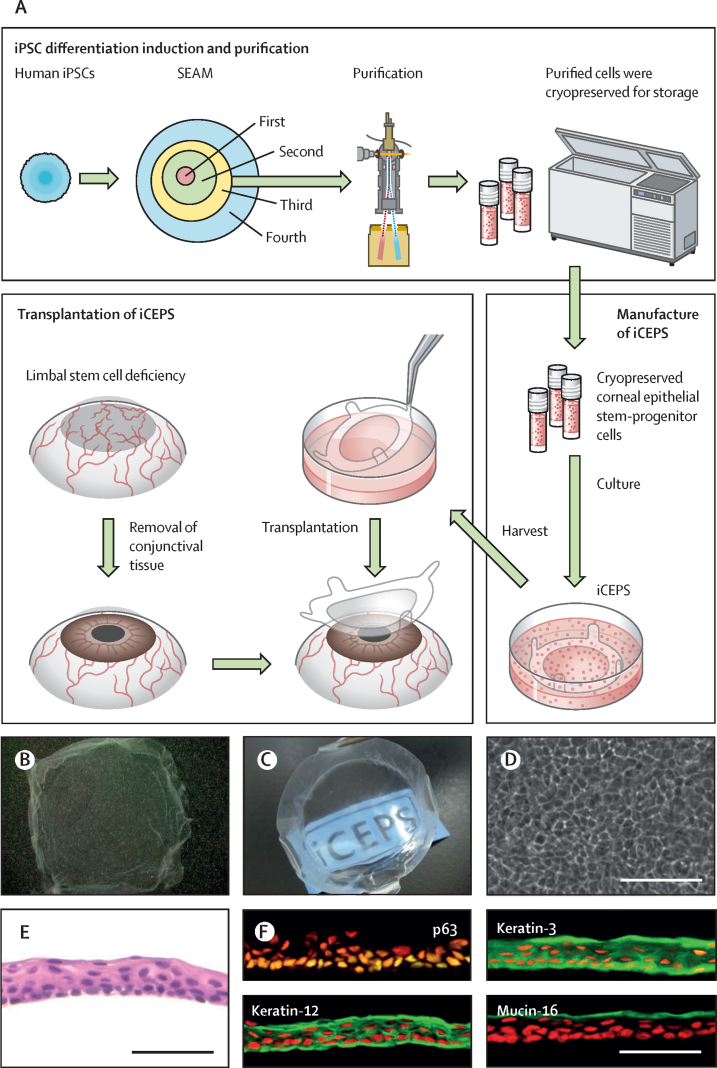
Fabrication and transplantation of human iCEPSs (A) A schematic of the whole procedure, starting with the cultivation of human iPSCs into SEAMs and the subsequent purification of corneal epithelial stem–progenitor cells from the third zone of the SEAM by means of a cell sorter. These cells were cryopreserved before their fabrication into iCEPSs at a good gene, cellular, and tissue-based products manufacturing practice-grade facility, and the subsequent transplantation of iCEPS onto affected eyes after removal of conjunctival tissue in patients with an LSCD. (B, C) Macrophotographs of iCEPSs before engraftment showing their transparent nature, with the text “iCEPS” visible below the construct. (D) Cobblestone-like appearance of cells in an iCEPS imaged by phase contrast microscopy. Scale bar, 100 μm. (E) iCEPSs are made up of three to five cell layers and resemble the normal corneal epithelium on haematoxylin and eosin staining. Scale bar, 50 μm. (F) iCEPSs are immunostained green and positive for p63, keratin-12, keratin-3, and mucin-16, identifiers of the corneal epithelium; the nuclei are shown in red. Scale bar, 50 μm. iPSC=induced pluripotent stem cell. iCEPSs=iPSC-derived corneal epithelial cell sheets. LSCD=limbal stem cell deficiency. SEAM=self-formed, ectodermal, autonomous, multi-zone.

Here, we report a novel regenerative therapy for an LSCD that uses human induced pluripotent stem cells (iPSCs; figure 1A). The approach is based on our development of a self-formed, ectodermal, autonomous, multizone (SEAM) cultivation protocol that effectively produces precursor cells of ocular tissues from human iPSCs.^16^ This groundbreaking method partly mimics whole-eye development, and from SEAMs we have successfully fabricated functional ocular tissues including conjunctiva and lacrimal gland.^17^, ^18^ The SEAM technology also facilitates the generation of human iPSC-derived corneal epithelial cell sheets (iCEPSs), which were able to reconstruct the cornea in an experimentally induced animal corneal injury model.^16^

Autologous therapy by means of iPSCs has advantages that include the avoidance of immunological rejection, but it also comes with disadvantages related to the time and expense required for cultivation and the need for tumourigenicity tests.^19^ Additionally, from a practical point of view, any instability in the quality of the iPSCs or the graft materials derived from them will lead to the unwanted cancellation of scheduled surgeries. With allogeneic therapy, there is a ready supply of cells, although immunological rejection now becomes an important consideration. However, somewhat unexpectedly, experiments have shown that corneal epithelial cell sheets derived from human iPSCs express lower levels of HLA class I and II compared with somatic cell-derived sheets.^20^ Indeed, mixed lymphocyte reaction tests showed no difference in the immune response to iCEPS between HLA-matched and HLA-mismatched peripheral blood mononuclear cells. Another important consideration is that the iCEPS construct does not contain immunocompetent cells. It has been reported that a high rate of rejection (about 40%) occurs following an allogeneic corneal limbal transplantation in patients with an LSCD.^10^ Previous studies have also indicated that donor-derived Langerhans cells promote early and acute corneal allograft rejection, acting in concert with allogeneic MHC-specific cytotoxic T cells. ^21^ Graft material for corneal limbal tissue transplantation contains copious antigen-presenting cells,^22^ which might increase the probability of a direct pathway to rejection; iCEPSs, however, do not contain immunocompetent cells because only induced corneal epithelial progenitor cells derived from SEAMs are used to fabricate them. Thus, we hypothesise that HLA compatibility and the use of immunosuppressive agents (above and beyond corticosteroid use) is not necessary for iCEPS transplantation, and incorporate an initial examination of this in our study design. Herein, we report the 52-week follow-up (plus an additional 1-year additional safety monitoring period) of the first-in-human iCEPS transplant surgery in four eyes of four patients with vision loss owing to a bilateral LSCD.

## Methods

### Study design and participants

We did a non-randomised, single-arm, clinical study involving four participants at the Department of Ophthalmology, Osaka University Hospital, Suita, Osaka, Japan. The follow-up period was 52 weeks, with an additional monitoring period up to week 104. The study protocol, amendments, and other related documents were reviewed to ensure adherence to the Act on the Safety of Regenerative Medicine, after which the study was granted approval by the First Certified Special Committee for Regenerative Medicine at Osaka University followed by the Subcommittee for Regenerative Medicine of the Health Sciences Council at the Japanese Ministry of Health, Labour and Welfare (approval number NA8140001). The authors vouch for the completeness and accuracy of the data and analyses and for the fidelity of the study protocol, which is included in the appendix. After explaining the clinical research by means of documents and audio support material^23^ to ensure adequate understanding by the patients with visual impairment, written informed consent was obtained. The study ended after the planned number of patients had been enrolled and the observational period had ended. The study complied with the principles of the Declaration of Helsinki.

**Figure 2:**
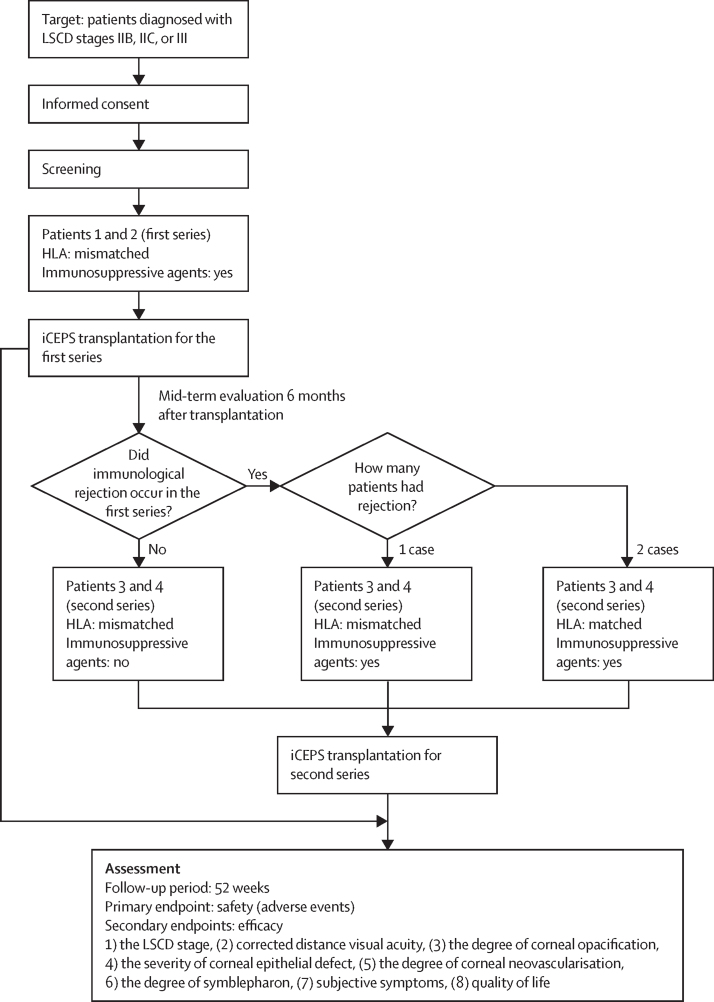
Flowchart of study design The flowchart outlines patient recruitment criteria and how the four recruited patients were split into two groups of two, with mid-term evaluations of immunological rejection in the first two patients (1 and 2) used to inform the use or not of HLA compatibility matching and immunosuppressive medication in the second set of two patients (3 and 4). The primary outcome measure was safety, assessed as the occurrence or absence of serious adverse events, with the criteria for the secondary outcome measure, efficacy, indicated at the bottom of the flowchart. LSCD=limbal stem cell deficiency. iCEPS=iPSC-derived corneal epithelial cell sheet. iPSC=induced pluripotent stem cell.

Inclusion criteria were contingent on a diagnosis of LSCD stage IIB, IIC, or III (appendix pp 17, 28). In addition, for iCEPS transplantation cases 1 and 2, patients were selected whose HLA type was mismatched with CiRA_F-supplied iPSC lines established from donors homozygous for the most common HLA haplotypes in Japan. Patients scheduled for surgeries 3 and 4 were selected on the basis of the number of cases of immunological rejection evident at the mid-term evaluation of the first two iCEPS transplantation procedures (figure 2). If neither of the first two surgeries rejected, or if only one did, then HLA-mismatched patients would be chosen for surgeries 3 and 4. If there were two cases of rejection, however, HLA-matched patients would be selected. Finally, all patients were required to be aged at least 20 years at the time of informed consent, and to have provided written consent to participate. Regarding exclusion criteria, patients were contraindicated if antibacterial drugs, corticosteroids, immunosuppressive agents, or anaesthetic drugs were used (appendix p 28). Likewise, patients were excluded if they were allergic to antibiotics, such as penicillin or streptomycin, or had a history of allergy to animals. They were also excluded if they had an active hepatitis B infection or a history of hepatitis B as established by HBs antigen, HBs antibody, or HBc antibody testing; had an active hepatitis C infection as established by hepatitis C virus (HCV) antibody or HCV-RNA testing; were positive for HIV antibodies; had presented with a malignant tumour within 5 years of screening, or were currently suspected to have a malignant tumour; had glaucoma with poorly controlled intraocular pressure; or suffered from diabetes with poor glycaemic control. Females who were pregnant or possibly pregnant, were lactating, or who planned to become pregnant during the course of the study were also excluded. Finally, individuals who had participated in another clinical study within 16 weeks of the planned transplantation date were not enrolled, nor were those currently participating, or scheduled to participate, in another clinical study, apart from observational research (appendix p 28). Patients were recruited through our referral networks as well as through institutional recruitment as part of the trial. Three cases were referred from outside Osaka University Hospital and one from within. We confirmed that all four patients met the inclusion criteria and enrolled them in sequence as consecutive cases.

We strictly adhered to the protocol for LSCD diagnosis using slit-lamp examination with fluorescein staining as recommended by the global consensus.^4^ We also confirmed the absence of palisades of Vogt (ie, radial infoldings of the limbus used as a clinical indication of the existence of corneal stem cells), and the presence of corneal opacity and superficial neovascularisation as observed by slit-lamp microscopy. Continuous invasion of hyper-reflective conjunctival epithelium onto the corneal surface was also identified by anterior segment optical coherence tomography, as instructed by the global consensus. As indicated in the research plan, for patients 1 and 2 (ie, the first pair of surgeries), HLA-A, HLA-B, and HLA-DR were mismatched by use of cells supplied by the clinical-grade iPSC haplobank at Kyoto University's Center for iPS Cell Research and Application Foundation (CiRA_F).^24^ As mentioned, we judge that immunosuppression is not required for iCEPS transplant surgery. However, following deliberations with the ethical committee from the perspective of patient protection and as a potential risk mitigation measure, we agreed to initially administer immunosuppressive agents, although at lower concentrations (100 ng/mL as a trough level) than used in other organ transplantations. A mid-term evaluation for signs of immunological rejection was carried out in patients 1 and 2, 6 months after surgery to establish the need for HLA compatibility matching and immunosuppressive treatment for the second pair of surgeries (ie, patients 3 and 4). Immunological rejection was established on the basis of three findings—corneal stromal oedema, ciliary injection, and corneal epithelial defects. This study is registered with UMIN, UMIN000036539.

### Procedures

A human iPSC line (clone ID YZWJs524) established by use of cord blood derived from an HLA-homozygous donor was obtained from the clinical-grade iPSC haplobank at CiRA_F,^24^ after which stocks were produced by the Contract Development and Manufacturing Organization at the TAKARA BIO Center for Gene and Cell Processing (Kusatsu City, Japan). From these stocks, iCEPSs were fabricated at a good gene, cellular, and tissue-based products manufacturing practice-grade facility, the Cell Processing Center of the Medical Center for Translational Research at Osaka University Hospital. Established standard operating procedures were followed and iCEPS fabrication was guided and recorded by means of a process management system. Before transplantation, iCEPSs were tested for quality control (total cell number, cell viability, cell purity, immunostaining for p63, ZO-1 [an immunofluorescent antibody] and mucin-16, plus droplet digital PCR for *LIN28A*; appendix p 25) and only those that met the criteria were used.

A single iCEPS was transplanted onto each of the patients' diseased eyes following procedures described in detail elsewhere (video).^8^, ^13^ Briefly, a keratectomy to remove sub-epithelial fibrotic tissue—including, importantly, tissue from the limbal region—was done before grafting the iPSC-derived epithelial sheets onto the patients' eyes. Thus, host corneal limbal epithelial cells did not remain in the diseased eyes, as has been shown in animal studies, whereby a keratectomy surgery leads to the total depletion of corneal epithelial stem cells.^25^ An iCEPS, which had been lifted from its temperature-responsive culture dish (appendix p 15), was then placed directly onto the corneal surface and sutured in place. Finally, a therapeutic soft contact lens was applied to the eye to protect the graft.

In all patients, postoperative medication included topical antibiotics (0·5% cefmenoxime) and corticosteroids (0·1% betamethasone) as eye drops four times a day, along with betamethasone and neomycin ointment once a day, to suppress postoperative inflammation. Systemic corticosteroid was administered as 125 mg methylprednisolone on the day of surgery, followed by 2 mg betamethasone for two days and 1 mg betamethasone for 1 month with tapering. In patients 1 and 2, cyclosporine was administered at a blood concentration of about 100 ng/ml as a trough level. This concentration is lower than that used in other organ transplantations but is effective in corneal graft surgery.^26^ Mycophenolic acid mofetil was used in patient 2 for the treatment of the primary disease, ocular mucous membrane pemphigoid. Therapeutic contact lenses were used during the entire postoperative follow-up period.

### Outcomes

As the primary endpoint measure, the safety of iCEPS transplantation was evaluated by monitoring adverse events. These were recorded and converted to standard terms by means of the Medical Dictionary for Regulatory Activities (version 23.0). We collected adverse events continuously throughout the whole observation period starting from the time of transplant up to the 2-year postoperative timepoint. The evaluation period was from the day of iCEPS transplantation (during transplantation) to week 52 after iCEPS transplantation or the date of discontinuation. The additional safety monitoring period (to week 104) was included for the reasons outlined in section 7 of the clinical protocol (appendix). Secondary endpoints as measures of the efficacy of iCEPS surgery were evaluated throughout the 52-week follow-up as follows: LSCD stage (appendix p 17);^4^ the presence or severity of a corneal epithelial defect; subjective symptoms; corrected distance visual acuity assessed by means of a decimal visual acuity chart and an Early Treatment Diabetic Retinopathy Study (ETDRS) letter chart; quality of life (QOL) as evaluated by the 25-item National Eye Institute Visual Function Questionnaire; the severity of corneal opacification;^27^ the severity of corneal vascularisation;^27^ and the existence and severity of symblepharon (a partial or complete adhesion of the palpebral conjunctiva of the eyelid to the bulbar conjunctiva of the eyeball).^27^ We assessed LSCD stage, corneal epithelial defects, subjective symptoms, visual acuity, corneal opacification, corneal neovascularisation, and symblepharon preoperatively and at 2, 4, 8, 16, 24, 32, 40, and 52 weeks postoperatively. QOL was assessed preoperatively and at the 52-week timepoint. The metrics shown in the appendix (pp 30–40, 45–56) were also examined at week 104. As with preoperative evaluations, the LSCD stage was established by slit-lamp examination with fluorescein staining as recommended by the global consensus (appendix p 17).^4^ Subinvestigators and evaluating physicians not directly involved in the research assessed and decided upon adverse events, the LSCD stage, corneal epithelial defect status, and the severity of corneal opacification, neovascularisation, and symblepharon (appendix pp 26–27). An assessment was also made, on the basis of interviews with the patients, about the influence of ocular adverse events on their daily activities. If serious adverse events occurred, the safety monitoring committee reviewed and provided its opinion on the clinical significance of causality of serious adverse events, the conduct of the research, the effect of information obtained from reports of research on similar treatments, the continuation of the entire clinical research (eg, if a neoplastic lesion developed), and the need for protocol revision. The funders played no role in data collection, analysis, interpretation, writing of the manuscript, or the decision to submit.

## Results

An iCEPS is a transparent cell sheet (figure 1B, C), comprised of cells with a cobblestone morphology (figure 1D) that form a multilayer (figure 1E). Within the iCEPS, expression of corneal epithelial-specific markers (p63, keratin-12, keratin-3, and mucin-16) was confirmed by immunostaining (figure 1F). Tumourigenesis tests for iCEPS included subcutaneous transplantation in immunodeficient mice (NOG/Shi-scid, IL-2RγKO Jic), karyotype tests, measurement of undifferentiated iPSC marker (*LIN28A*) expression, serial passage culture tests, and a genome analysis (appendix pp 13–14, 20–24). None of these investigations yielded results suggestive of tumourigenicity. As detailed, iCEPSs were subjected to quality control tests to confirm standard value compliance before they were provided for transplantation (appendix pp 16, 25).

Patients, who each self-identified their sex, were enrolled between June 17, 2019 and Nov 16, 2020, with the first two comprising a female aged 44 years with an idiopathic LSCD (patient 1) and a male aged 66 years with ocular mucous membrane pemphigoid (patient 2; table 1; appendix p 29). Both received an iCEPS transplantation (figure 2) and were followed up for the scheduled observation period. No clinical immunological rejection was seen in either patient at mid-term evaluations (appendix p 18). As a consequence, it was established that neither the use of immunosuppressive agents (above and beyond the topical and systemic application of corticosteroids) nor HLA compatibility matching would be required for the second pair of patients as had been stipulated in the clinical protocol (appendix p 29). The second pair of patients comprised a male aged 72 years with an idiopathic LSCD (patient 3) and a female aged 39 years with toxic epidermal necrosis (patient 4). All four patients self-identified as ethnic Japanese.

**Table 1:** Patient characteristics and clinical summary before and after induced pluripotent stem cell-derived corneal epithelial cell sheet transplantation

	**Patient number, sex, and age**	**Eye**	**Diagnosis**	**Safety endpoints**		**Efficacy endpoints**					
HLA compatibility (immunosuppressive agent)				Clinical rejection	Tumour formation	Before transplantation			52 weeks after transplantation		
						LSCD severity stage	CDVA decimal visual acuity (logMAR)	Corneal opacification grade	LSCD severity stage	CDVA decimal visual acuity (logMAR)	Corneal opacification grade
Mismatched (+)	1, female, 44 years	Left	Idiopathic LSCD	0	0	III	0·03 (1·52)	2·8	IA	0·3 (0·52)	0·5
Mismatched (+)	2, male, 66 years	Right	Ocular MMP	0	0	III	0·01 (2·00)	3·0	IA	0·15 (0·82)	1·0
Mismatched (−)	3, male, 72 years	Right	Idiopathic LSCD	0	0	IIB	0·15 (0·82)	1·0	IA	0·7 (0·16)	0·4
Mismatched (−)	4, female, 39 years	Right	Toxic epidermal necrolysis	0	0	III	0·02 (1·68)	3·0	IIB	0·04 (1·40)	1·6

The severity of corneal opacification was graded with a previously described system^27^ from 0 to 3. 0=clear cornea with iris details clearly visualised. 1=partial obscuration of the iris details. 2=iris details poorly seen with pupil margin just visible. 3=complete obscuration of iris and pupil details. LSCD=limbal stem cell deficiency. CDVA=corrected distance visual acuity. logMAR=logarithm of the minimum angle of resolution. MMP=mucous membrane pemphigoid.

No immunological rejection or tumour formation occurred (table 1), nor were any serious adverse events, as defined by the clinical protocol, seen. Thus, the safety monitoring committee were not called on. During the 52-week follow-up period, ten non-serious adverse events occurred following the first two surgeries, with 16 seen after the second two (table 2; appendix pp 30–31). These were not clinically significant and were readily managed without sequelae. Nine non-serious adverse events were documented during the 1-year additional safety monitoring period (weeks 52–104) and are reported in the appendix (p 32).

**Table 2:** Summary of adverse events during the 52-week follow-up period

	**Adverse events**	**Expression site**	Severity*	**Serious adverse event**	**Treatment for adverse events**	**Outcome of adverse events**	**Causality relationship between adverse events and the protocol**	**Cause of adverse events**
**First series**								
Patient 1								
	Eye complication associated with device†	Treated eye	Mild	No	Wear contact lens	Recovered	Possible	Surgery
	Eye pain	Treated eye	Mild	No	Analgesics	Recovered	Yes	Surgery
	Intraocular pressure increased	Treated eye	Mild	No	Antiglaucoma eye drops	Recovered	Possible	Corticosteroid
	Corneal epithelium defect	Treated eye	Mild	No	Use of minimal essential eyedrops	Unrecovered	Possible	Side-effect of eyedrops
	Cataract	Treated eye	Mild	No	None	Unrecovered	Possible	Corticosteroid
Patient 2								
	Constipation	Outside the eyes	Grade 1	No	Laxatives	Recovered	No	Random coincidence
	Corneal herpes	Treated eye	Mild	No	Anti-herpes drug	Recovered	Possible	Corticosteroid and immunosuppressive agents
	Cataract aggravated	Treated eye	Moderate	No	None	Unrecovered	Possible	Corticosteroid
	Corneal epithelium defect	Treated eye	Mild	No	Use of minimal essential eyedrops	Unrecovered	Possible	Side-effect of eyedrops
	Intraocular pressure increased	Treated eye	Mild	No	Anti-glaucoma eye drops	Recovered	Possible	Corticosteroid
**Second series**								
Patient 3								
	Constipation	Outside the eyes	Grade 1	No	Laxatives	Recovered	No	Random coincidence
	Corneal epithelium defect	Treated eye	Mild	No	Anti-herpes drug	Recovered	Possible	Mechanical trauma
	Corneal epithelium defect	Treated eye	Mild	No	Corticosteroid	Recovered	No	Primary disease process
	Conjunctival hyperaemia	Treated eye	Mild	No	Corticosteroid	Recovered	No	Primary disease process
	Intraocular pressure Increased	Treated eye	Mild	No	Anti-glaucoma eye drops	Recovered	Possible	Corticosteroid
	Corneal epithelium defect	Treated eye	Mild	No	None	Recovered	No	Primary disease process
	Corneal epithelium defect	Treated eye	Mild	No	Corticosteroid	Recovered	No	Primary disease process
	Corneal epithelium defect	Treated eye	Mild	No	Corticosteroid	Recovered	No	Primary disease process
Patient 4								
	Eye pain	Treated eye	Mild	No	Analgesics	Recovered	Possible	Surgery
	Constipation	Outside the eyes	Grade 1	No	Laxatives	Recovered	No	Random coincidence
	Conjunctival hyperaemia	Treated eye	Mild	No	Corticosteroid	Unrecovered	No	Primary disease process
	Cushingoid	Outside the eyes	Grade 1	No	None	Unrecovered	No	Corticosteroid
	Common cold	Outside the eyes	Grade 1	No	None	Recovered	No	Random coincidence
	Ocular hypertension	Treated eye	Mild	No	Anti-glaucoma eye drops	Unrecovered	No	Primary disease process
	White blood cells increased	Outside the eyes	Grade 1	No	None	Recovered	No	Random coincidence
	Decreased appetite	Outside the eyes	Grade 1	No	None	Unrecovered	No	Random coincidence

* The severity of adverse events outside the eyes were established using the Japanese Clinical Oncology Group Japanese translation of the Common Terminology Criteria for Adverse Events version 4.024) from grade 1 to 5. 1=mild; asymptomatic or mild symptoms; clinical or diagnostic observations only; intervention not indicated. 2=moderate; minimal, local or non-invasive intervention indicated; limiting age-appropriate instrumental activities of daily living. 3=severe or medically significant but not immediately life-threatening; hospitalisation or prolongation of hospitalisation indicated; disabling; limiting self-care activities of daily living. 4=Life-threatening consequences; urgent intervention indicated. 5=death related to adverse events. Adverse events localised to the eye were classified into three levels of severity (mild, moderate, or severe) as follows. Mild=Signs or symptoms that are easily bearable. Moderate=signs or symptoms that interfere with daily activities. Severe=signs or symptoms that hinder work or daily activities.

† Eye complication associated with device indicates dropout of a therapeutic soft contact lens.

**Figure 3:**
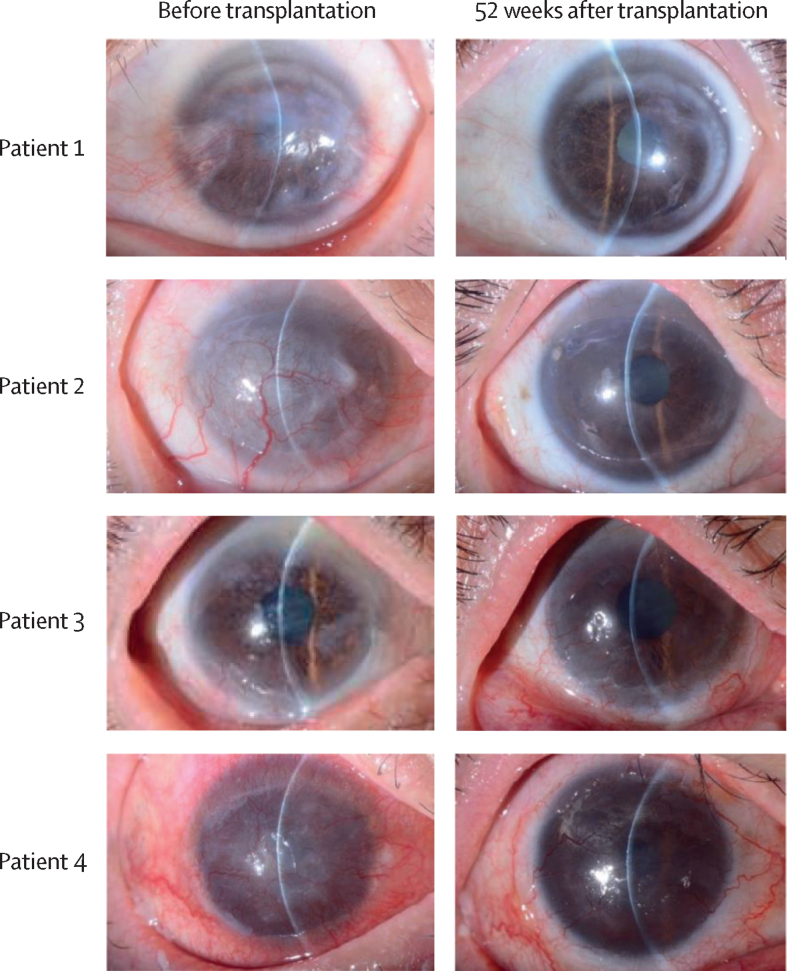
Slit-lamp microscopy images of the treated eyes Slit-lamp microscopic photographs of all four treated eyes before and 52 weeks after induced pluripotent stem cell-derived corneal epithelial cell sheet transplantation

**Figure 4:**
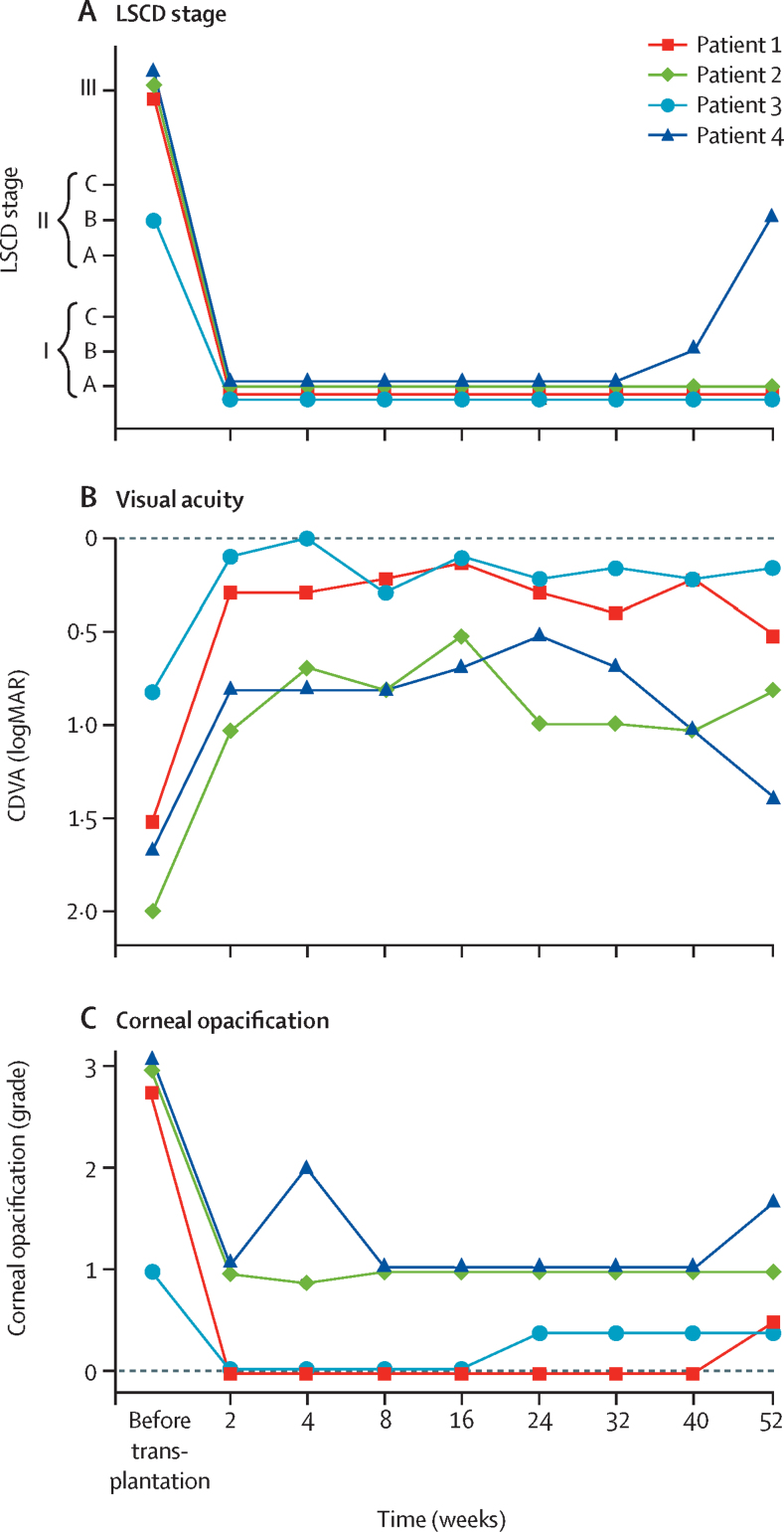
Stage of LSCD, visual acuity, and corneal opacification (A) The stage of LSCD before and after iCEPS transplantation. The LSCD improved from stage III to IA in two patients and from stage IIB to IA in one patient. This improvement was maintained throughout the whole 52-week follow-up period. Patient 4 had improved to LSCD stage IA at 32 weeks after transplantation. However, this had regressed to stage IIB one year after transplantation. (B) CDVA assessed by a decimal chart, which improved in all four patients postoperatively; CDVA is expressed as logMAR, which was converted from decimal visual acuity (a decrease of at least 0·2 in logMAR CDVA corresponds to an improvement of two lines or more on a Landolt C chart). (C) Corneal opacification improved in all four patients following iCEPS surgery. LSCD=limbal stem cell deficiency. CDVA=corrected distance visual acuity. logMAR=logarithm of the minimum angle of resolution. iCEPS=iPSC-derived corneal epithelial cell sheet. iPSC=induced pluripotent stem cell.

The clinical manifestations of LSCD improved in all patients following iCEPS transplant surgery (Figure 3, Figure 4; appendix pp 63–66). Specifically, LSCD improved from stages III to IA in patients 1 and 2 and from stages IIB to IA in patient 3 (appendix p 17). This recovery was maintained throughout the whole follow-up period. Patient 4, whose condition was the most severe, with toxic epidermal necrosis as the causative disease, had improved to LSCD stage IA at the 32-week juncture. However, this had regressed to stage IIB 1 year postoperatively (Figure 3, Figure 4; appendix p 32). Regarding the severity of the corneal epithelial defect, patient 1 was grade 0 preoperatively, whereas patients 2, 3, and 4 were grade 1. At postoperative week 52, patients 1, 2, and 3 were grade 0 and patient 4 was grade 1 (appendix pp 43–44), reflective of either stability or an improvement. Subjective symptoms were generally unchanged or improved (appendix pp 50–55). Corrected decimal distance visual acuity improved by 10·0 logarithm of the minimum angle of resolution (logMAR). lines in patient 1, by 11·8 lines in patient 2, by 6·6 lines in patient 3, and by 2·8 lines in patient 4 (figure 4B; appendix pp 33, 67). ETDRS visual acuity improved by 7·8 lines in patient 1, by 23·8 lines in patient 2, by 3·8 lines in patient 3, and remained unchanged in patient 4 (appendix pp 34–35). QOL scores at 52 weeks were higher in patients 1, 2, and 3 compared with preoperatively but had declined in patient 4 (appendix p 56). An assessment of corneal opacification showed an improvement in all four patients at 52 weeks compared with preoperative levels (figure 4C; appendix pp 36, 68). Corneal neovascularisation was less pronounced in patients 1 and 2, 52 weeks post-surgery, but was more prevalent or unchanged in patients 3 and 4 (appendix pp 45–47). The presence and severity of symblepharon remained the same at the 52-week juncture for all patients (appendix pp 48–49).

## Discussion

Here, we describe a new human iPSC-based transplant surgery to reconstruct the eyes of patients with vision loss owing to an LSCD. The procedure, grafting iCEPSs onto the ocular surface, was well-tolerated 2 years postoperatively in all four eyes involved in this first-in-human study. Because iCEPSs were generated from allogeneic iPSCs, safety issues—tumourigenesis and immunological rejection in particular—were paramount. In this regard, we found no evidence of serious adverse events (our primary endpoint measure) throughout the whole 2-year observational period in any of the patients. Our protocol-planned follow-up period was 52 weeks and the period to 104 weeks was an additional safety monitoring period. We also found improvements in the clinical stage of the LSCD and other secondary endpoints as measures of efficacy as shown in the appendix (pp 19, 33–58). This demonstration of efficacy in some patients is important, and the fact that successful outcomes were achieved without HLA matching suggests that iCEPS transplant surgery has real potential as a new treatment for patients with an LSCD.

Before doing the four surgeries, we confirmed that iCEPS had no tumourigenic potential as ascertained by subcutaneous transplantation into immunodeficient mice, karyotyping, measurement of *LIN28A*, serial passage culture tests, and genome analysis (appendix pp 13–14, 20–22). Genomic analysis of the same batch of iCEPS used for the surgeries indicated no single nucleotide polymorphisms or copy number polymorphisms with amino acid changes that correspond to the COSMIC census list, or a list generated by the Pharmaceuticals and Medical Devices Agency (appendix p 25). Thus, despite the low number of cases, early indications do not point to any major safety concerns regarding tumour formation for the future clinical development of iCEPS transplant surgery. Of note, it has been reported that the transplantation of iPSC-derived, terminally differentiated retinal pigment epithelial cells does not lead to tumour formation,^19^ and this appears to be the case with iCEPS transplant surgery, too.

Of the efficacy endpoints, LSCD disease stage is considered to be the most appropriate to capture the overall efficacy of iCEPS transplantation. This metric was also used as the primary endpoint in our previous examination of autologous cultivated limbal epithelial cell sheet transplantation.^8^ Following surgery, patients 1, 2, and 3 were all consistently at stage IA, indicating sustained efficacy. However, although patient 4 reached LSCD stage IA at postoperative week 32, by the time of the final examination at week 52 at the end of the follow-up period, this had receded to IIB, indicating unsustained efficacy. Across all efficacy endpoints, good results were achieved for patients 1 and 2. However, compared with patients 1 and 2, less success was achieved in reducing the amount of corneal neovascularisation in patients 3 and 4, and for reducing the corneal opacification and improving the QOL score for patient 4.

When we consider possible reasons for the low efficacy in patients 3 and 4, we are led to suspect that, to differing extents, both might have suffered from some subclinical chronic immunological rejection, which has been reported after limbal allograft surgery.^28^ Clinical immunological rejection in our study protocol was established by reference to direct clinical examination focused on corneal stromal oedema, ciliary injection, and corneal epithelial defects. Judged on these criteria clinical immunological rejection was not documented in any of the four treated eyes following HLA-mismatched iCEPS transplantation, and this was the case with or without the use of immunosuppressive medication. It is not possible, however, to categorically discount the possibility of subclinical chronic immunological rejection because it is difficult to identify. Therefore, the type of rejection defined in this study is clinical immunological rejection based on the stated criteria. Perhaps it is the case that the non-use of systemic cyclosporine in patients 3 and 4 might have triggered subclinical chronic immunological rejection, and that the poor clinical success in patient 4, in particular, might have been compounded by the aggressive nature of the underlying disease (toxic epidermal necrosis) and the relatively young age of the patient (39 years) compared with the others.

There are two prevailing hypotheses regarding the possible mechanism of action by which iCEPS transplant surgery was able to recover the corneal surface. The first is that the regenerated corneal epithelium is derived from cells within the iCEPS. Indeed, we have shown that an iCEPS is multistratified with three to four layers and that all the basal cells are p63 positive (figure 1F). We also know that an iCEPS contains stem cells with long-term proliferative potential.^16^ That they have lower immunogenicity compared with cultivated limbal epithelial cells, as shown by mixed lymphocyte reactions, would be an added benefit.^20^ The second hypothesis is that transplanted allogeneic iCEPSs are replaced by host conjunctival epithelial cells, and that these assume a corneal epithelial-like phenotype (a process called conjunctival transdifferentiation^29^) to recover the corneal surface in a vascular-free corneal environment.

With regard to these possibilities, it would be interesting to perform genotyping to know whether or not the transplanted iPSC-derived cells remain; however, it was decided (in conversations with regulatory bodies) that this should not be done because an extra surgical intervention might influence the study results, but more importantly would interfere with the healed corneal epithelium and put at risk ocular surface stability and the patients' sight. Moreover, genotyping would not be a definitive way to establish the mode of action for iCEPS transplantation because several reports of genotyping after allogeneic (cultivated) limbal transplantation have shown a mixture of host-derived cells and allogeneic donor-derived cells.^30^ This might also be the case in patients following iCEPS transplantation.

There are limitations to our study, not least the low number of cases, meaning that the safety and efficacy of iCEPS cannot be categorically concluded. It also means that we cannot compare the current results with those of other allogeneic therapies for bilateral LSCD, such as allogeneic cultivated limbal epithelial cell sheet transplantation or simple limbal epithelial transplantation. However, we would argue that the study design is appropriate when doing first-in-human surgeries from the perspective of patient safety. A second point, linked to the low initial number of patients concerns generalisability and the need to assess the applicability of iCEPS graft surgery to LSCDs of a range of aetiologies.

To our knowledge, this study provides the first description of iPSC-derived cell constructs being transplanted into or onto patients' corneas, and it represents a promising future treatment option for individuals with an LSCD. Accordingly, we plan to initiate a multicentre clinical trial to investigate the larger scale efficacy of treating LSCD using iCEPS, to build on the encouraging results described herein.

### Contributors

### Data sharing

All de-identified participant data, including individual participant data and the study protocol are available in the appendix.

## Declaration of interests

KN reports receiving research funds from RAYMEI, a company for which he is a stockholder. KN was not directly involved with the evaluation of efficacy and safety, data management, monitoring, or statistical analysis. After the study's completion HT became an employee of RAYMEI, but during the study had no competing interests. All other authors declare no competing interests. A conflict of interest management plan was submitted to and approved by the First Certified Special Committee for Regenerative Medicine, Osaka University.

## Supplementary Materials

Supplementary appendix

Supplementary Video ‘iPS cell-derived corneal epithelial cell sheet transplantation Patient 2: ocular mucous membrane pemphigoid' **YOUTUBE link:**https://youtu.be/ve1xguKeDQA
