# Supplementary material for: Induced pluripotent stem-cell-derived corneal epithelium for transplant surgery: a single-arm, open-label, first-in-human interventional study in Japan
Source: Lancet. 2024 Nov 16;404(10466):1929–39. doi: 10.1016/S0140-6736(24)01764-1 (PMC11694011; doi:10.1016/S0140-6736(24)01764-1)
Supplement: Supplementary appendix [file mmc1.pdf]

THE LANCET

Supplementary appendix

This appendix formed part of the original submission and has been peer reviewed. We post it as supplied by the authors.

Supplement to: Soma T, Oie Y, Takayanagi H, et al. Induced pluripotent stem-cell-derived corneal epithelium for transplant surgery: a single-arm, open-label, first-in-human interventional study in Japan. *Lancet* 2024; published online Nov 7. https://doi.org/10.1016/S0140-6736(24)01764-1.

Supplementary Appendix

iPS cell-derived corneal epithelium for transplant surgery:

A single arm, open-label, first-in-human interventional study

Takeshi Soma, M.D.,[1)]* Yoshinori Oie, M.D.,[1)]* Hiroshi Takayanagi, M.S.,[1)]* Shoko Matsubara, B.A.,[1)] Tomomi Yamada, Ph.D.,[2)] Masaki Nomura, Ph.D.,[3)] Yu Yoshinaga, M.D.,[1)] Kazuichi Maruyama, M.D.,[1,4,5)] Atsushi Watanabe, M.D.,[1)] Kayo Takashima, RN,[6)] Zaixing Mao, Ph.D.,[7)] Tomohiko Katayama, M.S.,[1)] Yuko Yasukawa, M.S.,[1)] Yurika Yamate, Ph.D.,[1)] Yuki Kobayashi, Ph.D.,[1)] Mio Morita, M.S.,[1)] Shohei Azuma, M.S.,[1)] Saki Inoue, B.A.,[2)] Yuki Ishikawa, M.S.,[1)] Kenji Suzuki, M.S.,[1)] Kaito Yamaguchi, M.S.,[1)] Zhenguo Wang, Ph.D.,[8)] Shiyi Liu, M.Sc.,[8)] Prof. Andrew J. Quantock, Ph.D.,[9)] Ryuhei Hayashi, Ph.D.,[1,10)] and Prof. Kohji Nishida, M.D.,[1,11)] **

1) Department of Ophthalmology, Osaka University Graduate School of Medicine, Osaka, Japan
2) Department of Medical Innovation, Osaka University Hospital, Osaka, Japan
3) Facility for iPS Cell Therapy, CiRA Foundation, Kyoto, Japan
4) Department of Vision Informatics, Graduate School of Medicine, Osaka University, Osaka, Japan
5) Integrated Frontier Research for Medical Science Division, Institute for Open and Transdisciplinary Research Initiatives (OTRI) Osaka University, Osaka, Japan
6) Uehiro Research Division for iPS Cell Ethics, Center for iPS Cell Research and Application, Kyoto University, Kyoto, Japan
7) R&D Division, Topcon Corporation, Tokyo, Japan
8) Topcon Advanced Biomedical Imaging Laboratory, Oakland, New Jersey, United States of America
9) The School of Optometry and Vision Sciences, Cardiff University, Cardiff, United Kingdom
10) Laboratory of Stem Cells and Applied Medicine, Osaka University Graduate School of Medicine, Osaka, Japan
11) Premium Research Institute for Human Metaverse Medicine (WPI-PRIMe), Osaka University, Osaka, Japan

*These authors equally contributed to the work
**Corresponding author

CONTENTS

SUPPLEMENTARY METHODS

1. **Study design**
2. **Fabricating an HLA-homozygous donor-derived iPS cell line**
3. **Fabricating iCEPS for nonclinical tests**
4. ***In vivo* tumourigenicity test**
5. ***In vitro* tumourigenicity tests**
   Karyotype test
   Undifferentiated iPSC maker (*LIN28A*) expression measurement test
   Serial passage culture test
6. **Genome analysis of iCEPS**
   Copy number variation (CNV) analysis with single nucleotide polymorphism (SNP)-genotyping array
   Whole genome and whole exome sequencing
7. **Fabricating iCEPS for transplantation**
   Fabricating the iPS cell stock
   Fabricating iCEPS for transplantation
8. **Quality control tests in iCEPS**
   Sheet peeling test
   Total cell number count, cell viability count, and cell purity test
   Immunofluorescence staining (p63, ZO-1, and Mucin-16)
   LIN28A expression measurement
   Sterility test
   Mycoplasma test
   Endotoxin test
9. **Analysis of iCEPS characteristics**
   Microscopic observation
   Haematoxylin and eosin staining
   Immunofluorescence staining
   Colony forming assay
10. **Patient recruitment**
11. **Clinical procedure**
12. **HLA genotyping**
13. **Midterm evaluation**
14. **Surgical procedure**
15. **Outcome evaluation**
16. **Corneal epithelium-specific antibody tests**
17. **Mixed lymphocyte reaction (MLR)**
18. **Statistical tests**

SUPPLEMENTARY FIGURES

Figure S1. *In vivo* tumourigenicity test.
Figure S2. *In vitro* tumourigenicity tests.
Figure S3. Components for iCEPS culture and shipment.
Figure S4. Colony forming assay.
Figure S5. Staging of limbal stem cell deficiency.
Figure S6. Slit-lamp photographs of patients 1 and 2 on midterm evaluation.
Figure S7. Slit-lamp photographs of patients before, 52, and 104 weeks after transplantation.

SUPPLEMENTARY TABLES

Table S1. Summary of *in vivo* tumourigenicity test.
Table S2. Number of SNVs and Indels and CNVs detected in iCEPS.
Table S3. Sequencing metrics of whole genome sequencing and exome sequencing.
Table S4. Quality control tests and genome analysis in iCEPS for transplantation.
Table S5. Schedule and examination.
Table S6. Inclusion and exclusion criteria.
Table S7. HLA-haplotype in donors and patients with LSCD.
Table S8. Adverse events up to the end of the 52-week follow-up period in the four patients.
Table S9. Adverse events recorded during the week 52-to-104 additional safety monitoring period in the four patients.
Table S10. The LSCD stage following iCEPS transplantation.
Table S11. Corrected distance visual acuity following iCEPS transplantation (decimal visual acuity converted to LogMAR).
Table S12. Corrected distance visual acuity following iCEPS transplantation (Early treatment diabetic retinopathy study total score).
Table S13. Corrected distance visual acuity following iCEPS transplantation (Early treatment diabetic retinopathy study visual acuity).
Table S14. Degree of corneal opacification following iCEPS transplantation.
Table S15. Corneal thickness (μm) following iCEPS transplantation.
Table S16. Transparency of the corneal epithelium following iCEPS transplantation.
Table S17. Corneal epithelial thickness (μm) following iCEPS transplantation.
Table S18. Severity of corneal epithelial defect following iCEPS transplantation.
Table S19. Degree of corneal neovascularisation following iCEPS transplantation.
Table S20. Degree of subepithelial corneal neovascularisation following iCEPS transplantation.
Table S21. Degree of symblepharon following iCEPS transplantation.
Table S22. Ocular pain following iCEPS transplantation.
Table S23. Foreign body sensation following iCEPS transplantation.
Table S24. Lacrimation following iCEPS transplantation.
Table S25. Photophobia following iCEPS transplantation.
Table S26. Dryness following iCEPS transplantation.
Table S27. Discomfort following iCEPS transplantation.
Table S28. Quality of life score using NEI VFQ-25 following iCEPS transplantation.
Table S29. National Eye Institute Visual Function Questionnaire-25 subscale scores following iCEPS transplantation.
Table S30. Immunological tests in patients with LSCD–corneal epithelium-specific antibody tests.
Table S31. Immunological tests in patients with LSCD–MLR.
Table S 32. A repeated measures ANOVA test with Bonferroni correction of LSCD stage.
Table S 33. A repeated measures ANOVA test with Bonferroni correction of CDVA (LogMAR).
Table S 34. A repeated measures ANOVA test with Bonferroni correction of corneal opacification (grade).

SUPPLEMENTARY REFERENCES

SUPPLEMENTARY METHODS

1. Study design

The primary endpoint of this study was to evaluate the safety of induced pluripotent stem cell-derived corneal epithelial cell sheet (iCEPS: refers to a sheet or sheets) transplantation by collecting adverse event data including immunological rejection and tumour formation in patients with a limbal stem cell deficiency (LSCD). Furthermore, the efficacy was evaluated using a variety of clinical measures to explore the feasibility of this treatment. This clinical study is designed as a single-centre, open-label, non-controlled study for the following reasons: 1) no definitive treatment is currently available for LSCD; 2) blinding is not feasible because the treatment involves surgery; and 3) the low prevalence of LSCD (the target disease of this study) would make random allocation of patients unfeasible.

In the first pair of surgeries (patient one and two), iCEPS transplantation was performed in human leukocyte antigen (HLA)-mismatched patients administered immunosuppressive agents. A preclinical study in cynomolgus monkeys conducted prior to this clinical study showed a low rate of rejection with HLA-mismatched corneal epithelial cell sheet transplantation even without the use of immunosuppressive agents.[1] Therefore, the administration of immunosuppressive agents should minimise the incidence of rejection. The protocol for the second pair of surgeries (patient three and four), which was based on the number of cases of uncontrolled rejection in the first two patients, was set as follows: 1) zero cases of rejection, which was set to not include the administration of immunosuppressive agents (above and beyond steroid use) to HLA-mismatched patients, to explore a protocol that could further reduce the patient burden. 2) One case of rejection: the protocol for cases three and four was set to the same protocol for cases one and two to continue investigating the possibility of protocol treatment with the administration of immunosuppressive agents in HLA-mismatched patients. 3) Two cases of rejection: the protocol was set so that cases three and four would be HLA-matched patients because the findings indicated that it would be difficult to control rejection by immunosuppressive agents in HLA-mismatched iCEPS transplantation.

The primary and secondary endpoints were assessed one year after transplantation. A comprehensive outline of the Clinical Study Protocol was provided.

2. Fabricating an HLA-homozygous donor-derived iPS cell line

Cord blood derived from an HLA-homozygous donor (donor ID: YZWJ) was used to establish induced pluripotent stem cells (iPSCs) by electroporation of episomal vectors.[2] The safety of the fabricated iPS cell lines was ensured by testing for residual episomal vectors, genetic mutations in cancer-related genes using genomic analysis, and sterility. The iPS cell line (clone ID: YZWJs524) fabricated in these ways was used in this study. HLA analysis of the HLA homozygous donor was outsourced to LSI Medience (Tokyo, Japan). The production of these iPS cell lines was conducted at the CiRA Foundation (CiRA_F, Kyoto, Japan).

3. Fabricating iCEPS for nonclinical tests

We fabricated iCEPS for nonclinical tests following the procedures described in the previous reports[3,4] using the HLA-homozygous donor-derived iPS cell line from CiRA_F. The iPS cell line obtained from CiRA F was subjected to passage culture, differentiation induction, and cell purification. The purified cells were frozen for storage, and iCEPS was fabricated using thawed cells. Cell purification was performed using a BD FACSAria II (BD Biosciences, Franklin Lakes, NJ, USA). Nonclinical tests, including tumourigenicity tests, were performed using iCEPS fabricated by these procedures. All genetic modification experiments using these human iPSCs were performed in accordance with the institutional guidelines of Osaka University and with the approval of the University's Gene Modification Experiments Safety Committee (Approval No: 03924).

4. *In vivo* tumourigenicity test

An *in vivo* tumourigenicity test was performed on immunocompromised animals to negate the tumourigenic potential of iCEPS. The conditions for this test were set based on the notification issued by the Ministry of Health, Labour and Welfare of Japan (MHLW).[5] The NOG/Shi-scid, IL-2RγKO Jic (NOG) mice (CLEA Japan, Inc, Kanagawa, Japan) were used as experimental animals. For each mouse, one sheet of iCEPS was embedded in 200 µL of Matrigel (BD Biosciences): StemFit medium AK-03 (Ajinomoto, Tokyo, Japan) (1:1) and injected through a subcutaneous incision. Undifferentiated iPSCs 201B7 (RIKEN Bio Resource Center, Ibaraki, Japan) grown on mouse embryonic fibroblasts (1×10^6 cells), which have been validated to stably form tumours, were injected subcutaneously into each mouse as a positive control. For the negative control, 200 µL of Matrigel and AK-03 inclusion material were injected subcutaneously. The number of mice per group was 12 and the observation period was up to 16 weeks. Autopsy and histopathological examination were performed at the end of the observation period. However, an autopsy was performed immediately if the tumour diameter exceeded 17 mm or if the mice lost more than 17% of their body weight within one week. The tumourigenic potential of iCEPS was evaluated based on the follow-up, autopsy, and histopathology results (Table S1). Immunohistochemical staining of injection sites and positive control tumour tissues was performed (Figure S1). The paraffin sections were deparaffinised, hydrophilised, and inactivated in a microwave oven for 10–60 min. After blocking with Tris-buffered saline (TBS) (TAKARA BIO Inc, Kusatsu, Japan.) containing 5% donkey serum (FUJIFILM Wako, Osaka, Japan) and 0·3% Triton X-100 (Sigma-Aldrich, St. Louis, MO, USA), Ki-67 antibody (Cell Signaling Technology, Danvers, MS, USA, catalog no. 9449) was added and incubated at 4 °C overnight. After the TBS wash, the samples were stained with Takara POD-conjugated anti-mouse antibody (TAKARA BIO Inc, catalog no. MK200) and evaluated using the Liquid DAB+ Substrate Chromogen System (Agilent Technologies, Santa Clara, CA, USA, catalog no. K3467). All animal experiments were approved by the Animal Ethics Committee of the Osaka University (Ref. No. 27-088-018) and were conducted in accordance with its regulations.

5. *In vitro* tumourigenicity tests

In vitro tests were conducted in accordance with the notification,[5] to evaluate the tumourigenic potential of iCEPS. *In vitro* tests comprehensively evaluated the tumourigenic potential of iCEPS by examining multiple qualities related to tumourigenic potential in separate tests.

Karyotype test

A karyotype test was performed to ensure that the iCEPS cells were free of chromosomal mutations. Chromosome integrity was evaluated in accordance with the International System for Human Cytogenetic Nomenclature-compliant criteria: structural changes or gain of the same chromosomal region in two or more different metaphase cells; chromosome deletions in at least three out of 50 cells by Giemsa staining and 20 cells by G-band (Figure S2A).

Undifferentiated iPSC maker (LIN28A) expression measurement test

To assess the residual undifferentiated pluripotent stem cells, a method was developed to measure the expression levels of the undifferentiated iPSC marker *LIN28A*[6]. To assess the persistence of undifferentiated iPSCs in iCEPS, the expression level of *LIN28A* was measured using QX200 digital droplet PCR system (Bio-Rad, Hercules, CA, USA). Spike tests were conducted to assess detection sensitivity. TERT-immortalised human corneal epithelial cells (C/TERTs) were obtained from Dr. J. Rheinwald Harvard Institute of Medicine, Boston, MA. Samples spiked with 1%, 0·1%, 0·01%, and 0·001% undifferentiated iPSCs in C/TERTs were analysed using spike tests. Subsequently, four iCEPS samples were used to measure expression levels (Figure S2B). Total RNA was extracted from the samples using Sepasol-RNA I Super G (Nacalai Tesque Inc, Tokyo, Japan). Drop formation was performed using QX200 Droplet Generator and expression measurements were performed using QX200 Droplet Reader. Measurements were recorded as *LIN28A* expression per 50 ng total RNA. The primer and probe sequences for the PCR tests and reaction conditions were previously reported.[6] This test was also applied as a quality control test for the iCEPS used for transplantation (Table S4).

Serial passage culture test

A serial passage culture test was performed to determine the cell growth limit to ensure that the cells comprising iCEPS have not become immortalised by transformation during the fabrication process.[7] Cells isolated from iCEPS were cultured in corneal epithelium maturation medium (CMM; Dulbecco's Modified Eagle Medium without glutamine and Nutrient Mixture F-12 Ham (3:1, Life Technologies, Carlsbad, CA, USA), 0·4 µg/mL hydrocortisone succinate (FIJIFILM Wako), 2 nM 3,3′,5-triiodo-l-thyronine sodium salt (MP Biomedicals, Irvine, CA, USA), supplemented with 5% fetal bovine serum (Nichirei Biosciences Inc, Tokyo, Japan), and 1 nM cholera toxin (List Biological Laboratory, CA), 2·25 µg/mL bovine transferrin HOLO form (Life Technologies), 2 mM L-glutamine, 0·5% insulin transferrin selenium solution (Life Technologies), and 1% penicillin-streptomycin solution) and passaged when sub-confluent. Passage cultures were continued until cell proliferation ceased. HeLa cells were used as positive controls, and passaging culture was performed in the same manner. The cumulative cell count was calculated by counting the number of cells during the passage to show the progress of proliferation (Figure S2C).

6. Genome analysis of iCEPS

Genomic analysis of iCEPS cells was performed to investigate the occurrence of genomic mutations caused by the fabrication process. Genomic variation was examined by comparing the genomic sequence of each sample with its origin; that is, cord blood cells. Single nucleotide variants (SNVs), insertions, deletions, copy number variations (CNVs), and collation with the COSMIC Cancer Gene Census[8] and Shibata's gene list[9] detected in iCEPS for nonclinical tests (one sample) and transplantation (four samples) relative to the origin are summarised in Tables S2 and S4.

Copy number variation analysis with single nucleotide polymorphism (SNP)-genotyping array

SNP-genotyping arrays HumanOmniExpress-24 (Illumina, CA, USA) were used for CNV calling. For analysing the nonclinical and clinical samples, v1.2 and v1.3 arrays were used, respectively. Genomic DNA (200 ng) was hybridised on DNA Bead Chips, and the intensities were scanned using an iScan (Illumina) following the manufacturer's protocol. After exporting the final reports using GenomeStudio 2011·1 (for v1.2 arrays) and 2·0 ·4 (for v1.3 arrays) (Illumina), CNV candidates were called as previously described[2] by comparing the results between the control and test samples followed by manual curation of the CNVs.

Whole genome and whole exome sequencing

Whole-genome sequencing (WGS) libraries for HiSeq2500 and NovaSeq6000 were generated using the KAPA Hyper Prep Kit (Kapa Biosystems, MS) without PCR using 200 and 420 ng of genomic DNA, respectively. Whole exome sequencing (WES) libraries for HiSeq2500 were prepared using the KAPA Hyper Prep Kit and sequencing libraries were constructed using SeqCap EZ Human Exome Library v3·0 (Roche, Basel, Switzerland) from 100 ng of genomic DNA. Whole exome sequencing libraries for NovaSeq6000 were generated from 50 ng of genomic DNA using the Twist Library Preparation EF Kit and Twist Comprehensive Exome. Libraries were sequenced with HiSeq2500 (Illumina) using HiSeq SBS Kit v4, and cluster generation was performed using HiSeq PE Cluster Kit v4-cBot and NovaSeq6000 (Illumina). All experiments were performed according to the manufacturer's instructions. After generating FASTQ files using bcl2fastq v2·17·1 14 for HiSeq2500 and v2·20·0 422 for NovaSeq6000 (Illumina), SNVs/Indels and CNVs were called as previously described.[2] The sequencing metrics are summarised in Table S3.

7. Fabricating iCEPS for transplantation

Fabricating the iPS cell stock

iPS cell stocks for transplantation were fabricated using an iPS cell line derived from CiRA_F HLA

homologous donors. iPS cell stocks were produced from an iPS cell line obtained from CiRA_F, expanded through two passaging cultures, and cryopreserved. Their fabrication was conducted at the Contract Development and Manufacturing Organization (CDMO), TAKARA BIO Inc, Center for Gene and Cell Processing.

Fabricating iCEPS for transplantation

Using the iPS cell stock fabricated in CDMA, iCEPS were fabricated using the same procedure as iCEPS for nonclinical tests. Cell purification was performed using a BD Influx (BD Biosciences). The cell sheet components for packaging were provided by Japan Tissue Engineering Co., Ltd., Gamagori, Japan. These components were used for culturing and shipment of iCEPS (Figure S3). Purified cells were seeded and cultured in an UpCell 3·5-cm dish (CellSeed Inc, Tokyo, Japan) with a harvest support ring. When the iCEPS was ready for shipment, the culture dish was attached to a dish holder and contained in a cell sheet container for shipping. Fabrication was performed at the Cell Processing Center of the Medical Center for Translational Research, Osaka University Hospital (MTR-CPC).

8. Quality control tests in iCEPS

Quality control tests were conducted to verify the quality of processed cellular products for transplantation. These tests were set up based on the notification issued by MHLW.[10] These tests were performed using one of the same batches of cellular products on the day before transplantation. The results of the quality tests for the four cases are summarised in Table S4. After confirming that the test results met the standard value of the management organisation of manufacturing facilities, the cellular products were shipped and provided for transplantation.

Sheet peeling test

The medium in the culture dish containing iCEPS was replaced with DPBS (-) (Life Technologies) containing 1 mM $CaCl_2$ (Otsuka Pharmaceutical Co., Ltd., Tokyo, Japan) and incubated at room temperature (RT: 1–30°C) for 10–15 min. After incubation, iCEPS were detached from the dish with forceps (together with the harvest support ring) to ensure that they were harvested without damage.

Total cell number count, cell viability count, and cell purity test

Half of the harvested cell sheets were digested with 0·25% trypsin-EDTA (Life Technologies) to isolate cells. A portion of the cells was stained with Trypan Blue Solution (0·4%; Life Technologies) and counted to calculate total cell number and cell viability. Isolated cells were fixed and permeabilised (BD Cytofix/Cytoperm kit, BD Biosciences) and stained with Keratin-14 antibody (Abcam, Cambridge, UK, catalog no. RCK-107), or normal IgG (Dako, catalog no. GA-75066-2) and analysed using a BD FACS Verse (BD Biosciences).

Immunofluorescence staining (p63, ZO-1, and Mucin-16)

One-quarter of the harvested cell sheets were embedded in Tissue-Tek O.C.T. The compound (Sakura Finetek Japan Co. Ltd., Tokyo, Japan) was frozen, thinly sliced using a CM3050S cryostat (Leica, Wetzlar, Germany) and mounted on a glass slide. The sections were fixed in acetone at −30°C for 20 min (p63 staining only) and washed with TBS. After incubation in TBS containing 5% donkey serum and 0·3% Triton X-100 for 1 h for blocking, the primary antibodies were applied and incubated at RT (1–30°C) (p63 staining at 37°C) for 90 min. Subsequently, three TBS washes for 10 min were performed, and the samples were incubated for 1 h at RT (1–30°C) with a 1:200 dilution of a secondary antibody and Hoechst 33342 (Sigma-Aldrich, catalog no. B2261) and observed using an Axio Observer D1 (Carl Zeiss, BW, Germany). Stem cell content was confirmed by anti-p63 staining, and barrier function factors by anti-ZO-1 and anti-Mucin-16 staining, respectively. The following antibodies were used: p63 (Abcam, catalog no. ab735, 1:50), ZO-1 (Thermo Fisher Scientific, catalog no. 339100, 1:100), Mucin-16 (Abcam, catalog no. ab693, 1:100),

and secondary antibody Alexa Fluor 488 (catalog no. A21202; 1:200) was used for labeling.

LIN28A expression measurement

LIN28A expression was measured using the digital droplet PCR system (Bio-Rad), following a previous method.[4] Total RNA was extracted from a quarter of the harvested cell sheets using Sepasol RNA I Super G (Nacalai Tesque Inc). Drop formation was performed using a QX200 Droplet Generator (Bio-Rad) and expression measurements were performed using a QX200 Droplet Reader (Bio-Rad). Measurements were recorded as *LIN28A* expression per 50 ng total RNA.

Sterility test

In this clinical study, sterility testing was conducted using Steritest NEO devices and Trypcase Soy Broth and Fluid Thioglycollate Medium (Merck Millipore, Burlington, MA) according to the membrane filter method described in the Japanese Pharmacopoeia. Specimens for testing were collected two weeks before transplantation and on the day of transplantation, and sterility testing was performed twice in each case.

Mycoplasma test

In this clinical study on iCEPS transplantation, mycoplasma testing was performed by real-time PCR using Myco Finder (Nissui, Tokyo, Japan) as the testing method according to the Japanese Pharmacopoeia. Specimens for testing were collected 2 weeks before-, 3 days before transplantation, and on the day of transplantation.

Endotoxin test

In this clinical study, endotoxin testing was conducted using *the Limulus ES-II* and a Toxinometer ET-6000 (FUJIFILM Wako) according to the Japanese Pharmacopoeia turbidimetric method. Specimens for testing were collected 2 weeks before transplantation, 3 days before transplantation, and on the day of transplantation.

9. Analysis of iCEPS characteristics

In addition to the quality control tests, the following analyses were conducted to examine iCEPS characteristics.

Microscopic observation

The cell morphology of the fabricated iCEPS was observed using an Axio Observer D1 (Carl Zeiss) (Figure 1D).

Haematoxylin and eosin staining

Fabricated iCEPS were fixed in 10% formaldehyde neutral buffer solution (Nacalai Tesque Inc) and embedded in paraffin. Sections of 3 µm thickness were cut and stained with haematoxylin and eosin (Sakura Finetek Japan Co., Ltd.) after deparaffinisation and hydration treatments. The sections were observed under a BZ-X800 microscope (KEYENCE, Osaka, Japan) (Figure 1E).

Immunofluorescence staining

The immunofluorescence staining procedure was like that used in the quality tests, except for the following steps. The sections were either fixed with cold methanol for 1 h or not fixed. Incubation conditions for treatment with the first antibody were overnight at 4°C or for 90 min at 37°C. The following primary antibodies were used: p63 (Abcam; catalog no. ab735, 1:50), Keratin-12 (Abcam; catalog no. ab185627,

1:1000), Keratin-3 (PROGEN, Heidelberg, Germany; catalog no. 61807, 1:200), and Mucin-16 (Abcam; catalog no. 901301, 1:100). The secondary antibody treatment was subsequently performed at RT (1–30°C) for 1 h using Alexa Fluor 488 (Life Technologies, catalog no. A32776, A32790, 1:200). The sections were observed using an FV3000 microscope (Evident, Tokyo, Japan) (Figure 1F).

Colony forming assay

Purified cells for iCEPS fabricating were seeded on MMC-treated NIH-3T3 feeder layers at 500–750 cells per well. They were cultured in CMM for 11–12 days, fixed in 10% formaldehyde neutral buffer solution (Nacalai Tesque Inc), and stained with Rhodamine B (FUJIFILM Wako). Colonies were confirmed using the EVOS cell imaging system M7000 (Thermo Fisher Scientific, Waltham, MA, USA) and colony formation efficiency (%) were calculated. The results for the four iCEPS fabricating cases are presented in Figure S4.

10. Patient recruitment

The inclusion and exclusion criteria are presented in Table S6 (also refer to the Clinical Study Protocol). Briefly, patients with stage IIB, IIC, or III (Figure S5) were included, and those with severe glaucoma, diabetes mellitus, allergy to antibiotics or animals, or a history of malignancy in the last 5 years were excluded. For cases one and two, patients whose HLA type was mismatched with CiRA-supplied iPS cell lines were enrolled. For cases three and four, HLA compatibility was determined by the number of rejections in the first two cases.

11. Clinical procedure

All clinical procedures were conducted in accordance with the established protocols for the clinical study. In all cases, subject characteristics, ophthalmologic examinations, and systemic examinations were performed at the time of screening. Subject characteristics included 1) date when informed consent was obtained; 2) date of birth; 3) sex; 4) causative disease of LSCD; 5) pre-existing medical conditions (ocular and non-ocular); 6) comorbidities and their severity (ocular and non-ocular), 7) history of eye surgery, 8) target eye of iCEPS transplantation, 9) HLA type, and 10) infectious disease status. The HLA type was determined through the secondary use of test results from another clinical study which was conducted at our hospital, “HLA haplotype distribution and immune response to therapeutic iPSCs derived from HLA-homozygous donors in patients with LSCD.”

Ophthalmologic examination included corrected distance visual acuity test using decimal corrected visual acuity and an Early Treatment Diabetic Retinopathy Study (ETDRS) chart, slit-lamp microscopy (Topcon SL-D7; Topcon, Tokyo, Japan), anterior segment optical coherence tomography with CASIA2 (Tomey, Nagoya, Japan), RTVue-100 (Optovue, Inc, Fremont, CA), DRI OCT Triton (Topcon, Tokyo, Japan), subjective symptoms (eye pain, foreign body sensation, lacrimation, photophobia, dryness, and discomfort), and quality of life (QOL) based on the 25-item National Eye Institute Visual Function Questionnaire (NEI VFQ-25). The patients underwent comprehensive laboratory tests, including haematological and urine analyses to assess any irregularities and infections in general conditions. Furthermore, tumour marker evaluations were performed. Subsequently, ophthalmological and systemic examinations were performed according to a predetermined timetable at regular intervals after enrollment.

12. HLA genotyping

HLA analysis of the HLA homozygous donor was outsourced to LSI Medience (Tokyo, Japan). For each patient, peripheral blood samples were collected and genotyped for HLA-A, -B, -C, -DRB1, -DQB1, and -DPB1 using next-generation sequencing high-resolution HLA typing at the HLA Foundation Laboratory

(Kyoto, Japan) (Table S7).

13. Midterm evaluation

A midterm evaluation was conducted 24 weeks after the second subject underwent transplantation (Figure S6). The midterm evaluation consisted of evaluation of immunological rejection. Rejection status was comprehensively determined based on findings, including corneal stromal oedema, ciliary injection, and corneal epithelial defect.

14. Surgical procedure

Superficial conjunctival scar tissue covering the corneal surface was dissected to expose the bare corneal stroma up to 3 mm outside the limbus. In subject two, a case of LSCD associated with ocular mucous membrane pemphigoid accompanied by severe symblepharon, amniotic membrane transplantation was performed over the sclera. The cell sheet was lifted from its temperature-responsive culture dish, directly grafted onto the corneal stroma, and sutured. A therapeutic soft contact lens was placed on the eye to protect the ocular surface after checking the transplanted cell sheets for epithelial defects using 0·5% fluorescein solution.

15. Outcome evaluation

The safety of iCEPS transplantation was assessed by collecting all adverse events, including immunological rejection and tumour formation, during the 52-week follow-up period subsequent to transplantation (Table S8). An additional one-year monitoring period collected adverse events up to week 104 (Table S9).
Secondary endpoints were as follows: 1) the LSCD stage, 2) corrected distance visual acuity using a decimal visual acuity chart and an ETDRS chart, 3) the degree of corneal opacification, 4) the severity of corneal epithelial defect, 5) the degree of corneal neovascularisation, 6) the degree of symblepharon, 7) subjective symptoms, and 8) QOL as evaluated by NEI VFQ-25. All were reported for the 52-week follow up, with some recorded at the 104-week endpoint of the additional monitoring period as described in the following figures and tables.

1) The LSCD stage: severity was determined by slit-lamp microscopy using the severity classification of LSCD (Figure S5 and S7, Table S10).[11]

2) Corrected distance visual acuity: visual acuity was measured using the standard visual acuity table specified in JIS T 7309 (Landolt ring chart) converted to LogMAR (Table S11). Visual acuity was measured using the ETDRS visual acuity test chart (Tables S12 and S13).

3-1) The degree of corneal opacification: The cornea was divided radially into eight sections, and the degree of corneal opacification[12] in each section was determined using slit-lamp microscopy (Table S14).

Grade 0: The cornea is transparent, and the iris can be observed in detail.
Grade 1: Details of the iris can be partially observed.
Grade 2: The iris details cannot be clearly observed, and the rim of the pupil is scarcely observed.
Grade 3: Neither the iris nor rim of the pupil are observed.
3-2) Corneal thickness: the cornea was radially divided into eight sections, and the corneal thickness in each section was measured using AS-OCT CASIA2 (Table S15).
3-3) Transparency of the corneal epithelium: a commercially available SS-OCT device (DRI OCT Triton, Topcon, Tokyo, Japan) was used to image the corneal area using 3×3 mm^2 volume scans. The average

OCT intensity was computed for the segmented epithelium layer within the eight selected distinct segments (Table S16).
3-4) Corneal epithelial thickness: the cornea was radially divided into eight sections, and the epithelial thickness was measured using RTvue-100 (Table S17).

4) The severity of corneal epithelial defect: determined by slit-lamp microscopy (Table S18).[12]
Grade 0: No corneal epithelial defect.
Grade 1: Corneal epithelial defect in <1/4 of the surface of the cornea.
Grade 2: Corneal epithelial defect in ≥1/4 and <1/2 of the surface of the cornea.
Grade 3: Corneal epithelial defect in ≥1/2 of the surface of the cornea.

5-1) The degree of corneal neovascularisation: the cornea was radially divided into eight sections, and the degree of corneal neovascularisation was determined using slit-lamp microscopy[12] and OCT (Table S19).
Grade 0: No neovascularisation.
Grade 1: Neovascularisation around the cornea.
Grade 2: Neovascularisation to the rim of the pupil.
Grade 3: Neovascularisation exceeding the rim of the pupil.
5-2) The degree of subepithelial neovascularisation: the cornea was radially divided into eight sections, and the degree of subepithelial neovascularisation was determined by slit-lamp microscopy[12] and OCT (Table S20).
Grade 0: No neovascularisation.
Grade 1: Neovascularisation around the cornea.
Grade 2: Neovascularisation to the rim of the pupil.
Grade 3: Neovascularisation exceeding the rim of the pupil.

6) The degree of symblepharon: determined by slit-lamp microscopy[12] (Table S21).
Grade 0: No symblepharon.
Grade 1: Symblepharon limited to the surface of the conjunctiva.
Grade 2: Symblepharon <1/2 of the surface of the cornea.
Grade 3: Symblepharon ≥1/2 of the surface of the cornea.

7) Subjective symptoms: the severity of subjective symptoms (eye pain, foreign body sensation, lacrimation, photophobia, dryness, and discomfort) was evaluated through patient interviews (Tables S22–S27).
Grade 0: No symptoms.
Grade 1: Mild symptoms.
Grade 2: Moderate symptoms.
Grade 3: Severe symptoms.
Grade 4: Markedly severe symptoms.

8) Quality of life related to visual function was evaluated using the Japanese version of the NEI VFQ-25 (Tables S28 and S29).

16. Corneal epithelium-specific antibody tests

Corneal epithelium-specific antibody tests were conducted using a partially modified method of a previous report.[13] Cultured iCEPS in a 96-well plate were fixed with methanol (−30°C) overnight or longer after removing the culture supernatants. They were subsequently washed three times with TBS, and permeabilised and blocked with TBS containing 1% BSA and 0·3% Triton X-100 for 1 h at room temperature. Serum samples collected preoperative and after surgery (×500) or commercially available (control, ×500), Keratin-14 (positive control, 1 μg/mL, rabbit IgG, BioLegend), and isotype control rabbit IgG (negative control for Keratin-14, 1 μg/mL, Bio-Techne, Minneapolis, MN, USA) were used as primary antibodies. Additionally, iCEPS without primary antibodies (negative control for serum samples) was included. The iCEPS cells were incubated with or without primary antibodies at 4°C overnight, washed three times with 0·05% Tween in TBS for 10 min for serum samples and negative control for serum samples,

and stained with secondary antibodies for 1 h at RT (1–30°C). Alexa Fluor 488 anti-human IgG (20 µg/mL, Thermo Fisher Scientific) was used as the secondary antibody in samples with serum as the primary antibody and in negative control samples without primary antibody. Alexa Fluor 488 anti-rabbit IgG (20 µg/mL, Thermo Fisher Scientific) was used as the secondary antibody in samples with anti-Keratin-14 antibody as the primary antibody and in samples with rabbit IgG isotype control. Cell nuclei were counterstained with Hoechst 33342 (Merck Millipore, Burlington, MA, USA). Mean fluorescence intensity was measured using a high-content imaging system (Operetta, PerkinElmer, Waltham, MA, USA). A well containing only TBS without iCEPS was used as a blank control (Table S30).

17. Mixed lymphocyte reaction (MLR)

Blood samples were collected from the recipients to perform the MLR assay. Subsequently, peripheral blood mononuclear cells (PBMCs) were isolated from transplanted recipients as responder effector cells and co-cultured with single iCEPS cells as target cells for 7 days. As a positive control for allogeneic immune rejection, transformed B cells were prepared from HLA-mismatched healthy adult donors after obtaining informed consent.[14] The culture medium and detailed methods were previously reported.[1] Before the assay, B cells and single iCEPS cells were incubated for 45 min at 37°C with 50 µg/mL mitomycin C to inactivate their cell proliferative activity. The supernatants were harvested after 7 days and assessed for IFN-γ secretion using Quantikine Human IFN-γ ELISA Kit (R&D Systems, Minneapolis, MN, USA). Peripheral blood mononuclear cells and PBMCs co-cultured with single iCEPS cells or transformed B cells were harvested for flow cytometric analysis.

The harvested PBMCs In MLR were incubated with APC anti-CD4 antibody (Miltenyi Biotec, Bergisch Gladbach, Germany, catalog no. 130-091-232 or 130-113-250), APC anti-CD8a antibody (Thermo Fisher Scientific, catalog no. 17-0088-42), APC anti-CD11b antibody (Miltenyi Biotec, catalog no. 130-091-241 or 130-113-231), and FITC anti-CD56 antibody (BioLegend, catalog no. 304604) for 1 h at RT (1–30°C). Intracellular staining with PE anti-human Ki-67 antibody (BioLegend, catalog no. 350504) was performed using fixation and permeabilisation solutions (BD Cytofix/Cytoperm kit, BD Biosciences). All samples were analysed using a FACSVerse flow cytometer (BD Biosciences). Data were analysed using FlowJo software (BD Biosciences) (TableS31).

18. Statistical tests

Statistical tests were performed to compare preoperative and postoperative LSCD stage, corrected distance visual acuity (CDVA; decimal visual acuity) and corneal opacification at each visit of four patients using the Friedman test and a repeated measures ANOVA. The contrast setting was a comparison of eight preoperative and eight postoperative time points (2, 4, 8, 16, 24, 32, 40 and 52 weeks after transplantation) for repeated measures ANOVA, and the Bonferroni method was used for multiple comparisons. The F values with (1, 3) degrees of freedom and unadjusted P values of the test results are shown in tables S32-S34. The time points at which statistically significant differences were found are indicated by *.

The Friedman non-parametric test demonstrated that there were significant differences in the LSCD stage, CDVA and corneal opacification values throughout the follow-up period (p-values of 0·001, 0·03 and 0·01, respectively). Despite the difficulty in demonstrating a normal distribution due to the limited number of cases in this study (n = 4), we performed a repeated measures ANOVA on the assumption that these outcomes follow a normal distribution, particularly visual acuity, which typically follows a normal distribution. To ensure that the pairwise comparisons in the post hoc analysis were conducted with the Bonferroni correction, we applied one of the strictest correction methods. Tables S32-34 in the supplementary appendix show the ANOVA data, which are consistent with those obtained by the aforementioned Friedman test.

SUPPLEMENTARY FIGURES

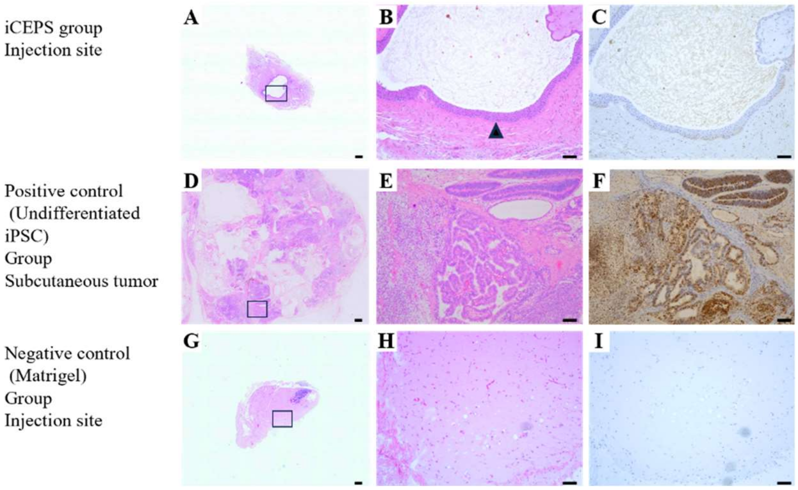

Figure S1. ***In vivo*** **tumourigenicity test**
As the histopathology results of the subcutaneous injection test in NOG/Shi-scid, IL-2RγKO Jic (NOG) mice, the upper rows (A–C) show injection sites in the induced pluripotent stem cell-derived corneal epithelial cell sheet (iCEPS)-treated group, the middle rows (D–F) show subcutaneous tumour tissue in the positive control group, and the lower rows (G–I) show injection sites in the negative control group. The squares in the histopathology images on the left (A, D, G) are enlarged in the middle (B, E, H). The right column (C, F, I) shows anti-Ki-67 immunohistochemical staining images. The arrowhead in (B) indicates residual transplanted iCEPS. The iCEPS group did not have increased injection sites, like the negative control group. Positive control group showing cell expansion and tumour formation. (A, D, G): Scale bar, 500 μm. (B, C, E, F, H, I): Scale bar, 100 μm.

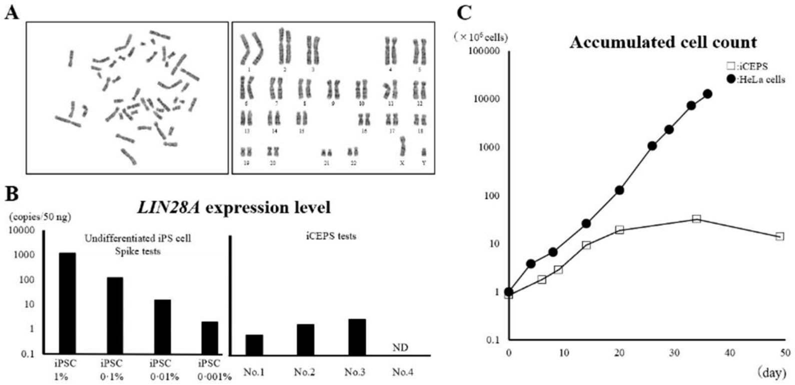

Figure S2. ***In vitro*** **tumourigenicity tests**
(A) iCEPS karyotype test (G-band). Chromosome mitotic image on the left and karyogram on the right. The karyotype was normal (46, XY). (B) *LIN28A* expression measurement (digital droplet PCR). Left: spike samples, right: four iCEPS samples refer to expression levels. *LIN28A* expression in iCEPS was extremely low and close to the detection limit, comparable to the iPSC 0·001% spike sample. (C) Serial passage culture tests. The increase in the number of accumulated cells in iCEPS and positive control (HeLa cells). Positive control (HeLa cells) continued to proliferate, whereas iCEPS showed reduced proliferation after 30–40 days.

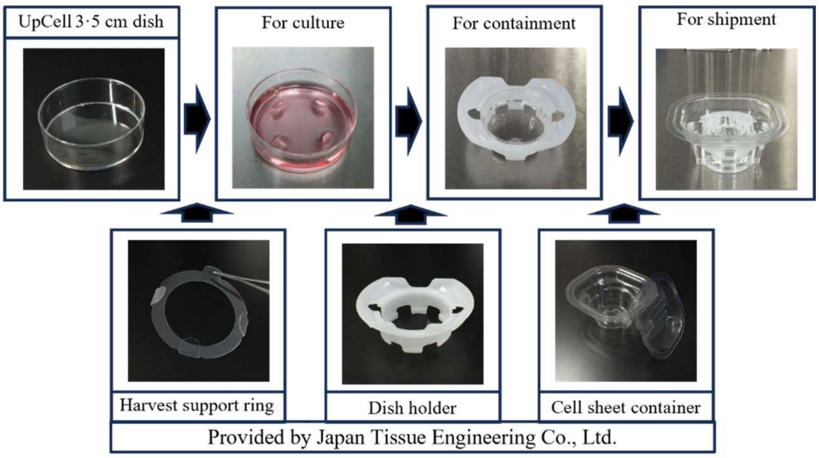

Figure S3. Components for iCEPS culture and shipment

The components used to culture and ship iCEPS are shown. The harvest support ring, dish holder, and cell sheet container were provided by Japan Tissue Engineering Co. Ltd. Cells were cultured on UpCell 3·5 cm dishes with a harvest support ring attached. At the time of shipment, the dish holder was attached and packaged in the cell sheet container.

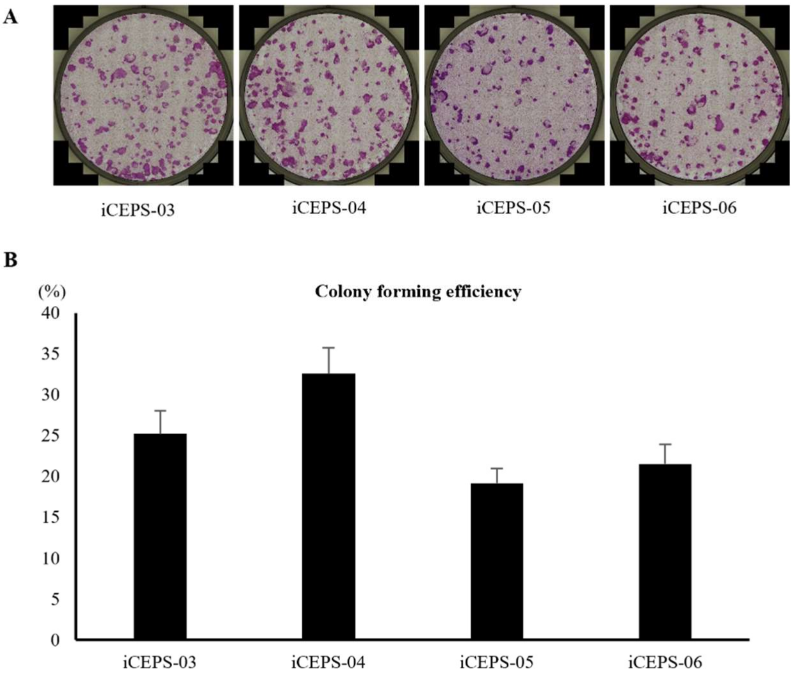

Figure S4. Colony forming assay

(A) Colony formation images of four cases from iCEPS-03 to -06. The number of cells seeded was 750 cells/well. (B) Colony forming efficiency in the fabrication of iCEPS-03 to -06. The colony forming efficiency of iCEPS-03 to -06 was approximately 20%–30%. Error bars indicate standard deviation.

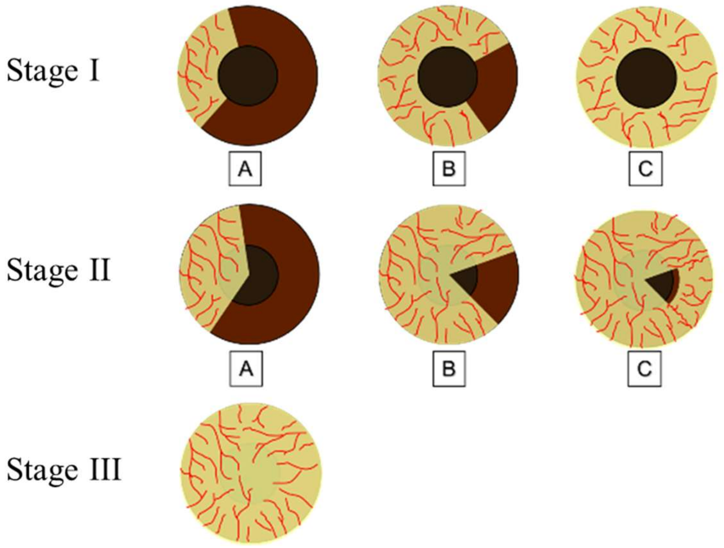

Figure S5. Stage of limbal stem cell deficiency
Staging of LSCD was performed based on the slit-lamp examination using a previously described system[11] with a minor modification.
Stage I: No conjunctivalisation is present in the centre of the cornea (diameter 5 mm), and the percentage of conjunctivalisation in the limbus is as follows: A: <50%, B: ≥50% and <100%, C: 100%.
Stage II: Conjunctivalisation is present in the centre of the cornea (diameter 5 mm), and the percentage of conjunctivalisation in the limbus is as follows: A: <50%, B: ≥50% and <100%, C: 100%.
Stage III: The whole corneal surface is covered by the conjunctival tissue.

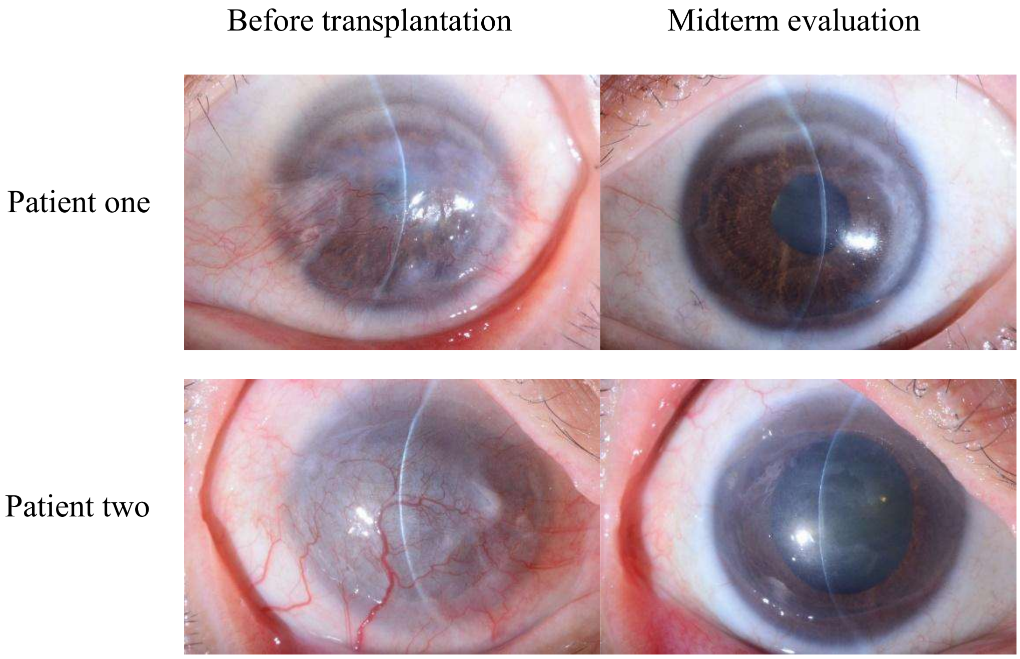

Figure S6. Slit-lamp photographs of patients one and two on midterm evaluation

Midterm evaluation was performed for the two patients of the first series at postoperative 10 months for patient one, and postoperative 6 months for patient two to determine the requirement of HLA compatibility and immunosuppressive agents for the subsequent two patients of the second series. No immunological rejection was observed in either patient one or patient two. Preoperative photographs of patients 1 and 2 are also shown in Figure3.

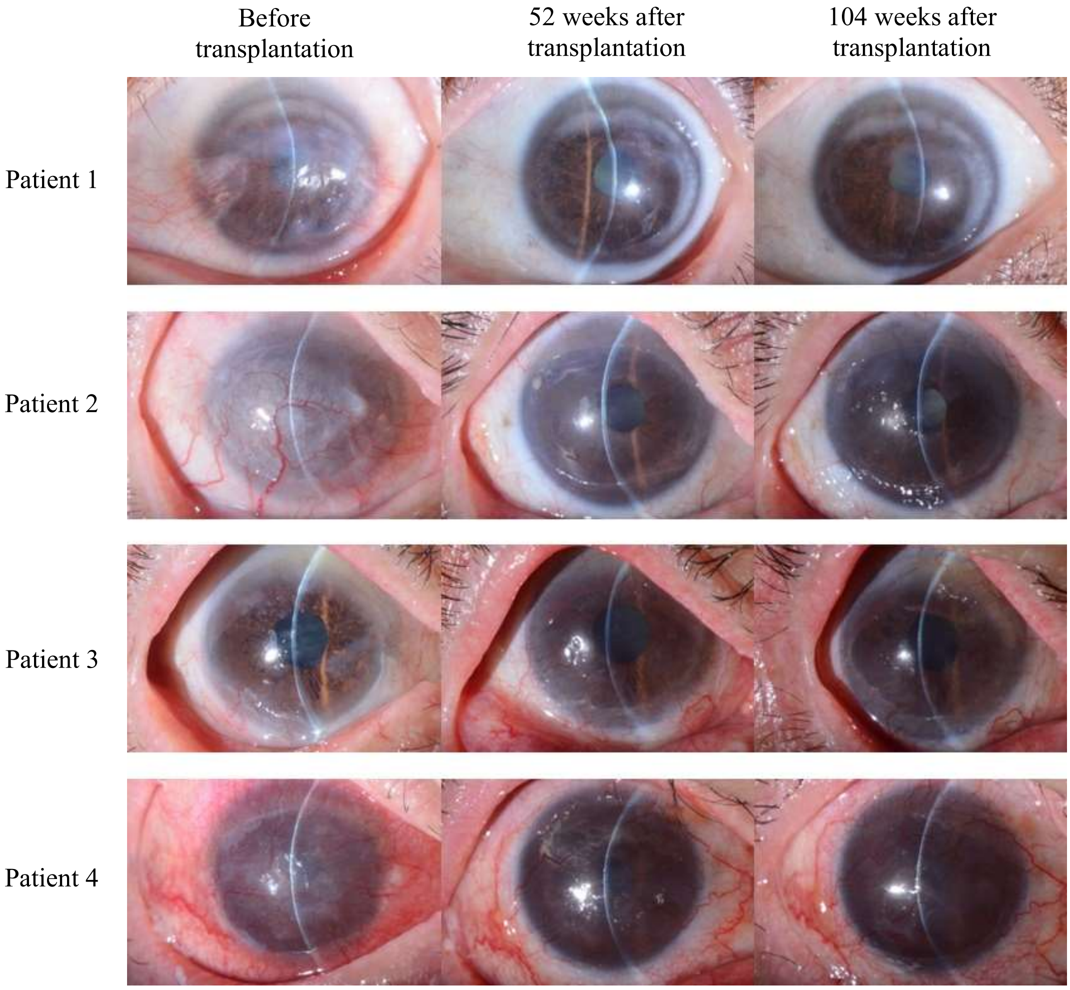

Figure S7. Slit-lamp photographs of patients before, and 52 and 104 weeks after transplantation
In all cases, the corneas maintained their condition at 104 weeks postoperatively. Preoperative and 52 weeks postoperative photographs are also shown in Figure3.

SUPPLEMENTARY TABLES

Table S1. Summary of *in vivo* tumourigenicity

Necropsy

Dose sample	Animal no.	Inspection site						Abnormal findings
		Brain	Lung	Liver	Kidney	Spleen	Injection site	
iCEPS*	B1[(1]	−†	−	−	−	−	−[#1]	
	B2[(1]	−	−	−	−	−	−[#1]	
	B3[(1]	−	−	−	−	−	−[#1]	
	B4[(1]	−	−	−	−	−	−[#1]	
	B5[(1]	−	−	−	−	−	−[#1]	
	B6[(1]	−	−	−	−	−	−[#1]	
	B7[(1]	−	−	−	−	−	−[#1]	
	B8[(1]	−	−	−	−	−	−[#1]	
	B9[(1]	−	−	−	−	−	−[#1]	
	B10[(1]	−	−	−	−	−	−[#1]	
	B11[(1]	−	−	−	−	−	−[#1]	
	B12[(1]	−	−	−	−	−	−[#1]	
Positive control (iPSC 201B7 on MEF)	G1[(7]	−	−	−	−	−	+§	
	G2[(6]	−	−	−	−	−	+	
	G3[(7]	−	−	−	−	−	+	
	G4[(7]	−	−	−	−	−	+	
	G5[(2]	−	−	−	−	−	+	
	G6[(7]	−	−	−	−	−	+	
	G7[(3]	−	−	−	−	−	+	
	G8[(4]	−	−	−	−	−	+	
	G9[(6]	−	−	−	−	−	+	
	G10[(5]	−	−	−	−	−	+	
	G11[(4]	−	−	−	−	−	+	
	G12[(6]	−	−	−	−	−	+	
Negative control (Matrigel only)	R1[(2]	−	−	−	−	−	−[#1]	+[#2]
	R2[(1]	−	−	−	−	−	−[#1]	
	R3[(1]	−	−	−	−	−	−	
	R4[(1]	−	−	−	−	−	−	
	R5[(1]	−	−	−	−	−	−[#1]	
	R6[(1]	−	−	−	−	−	−[#1]	
	R7[(1]	−	−	−	−	−	−	
	R8[(1]	−	−	−	−	−	−	
	R9[(1]	−	−	−	−	−	−[#1]	
	R10[(1]	−	−	−	−	−	−	
	R11[(1]	−	−	−	−	−	−	
	R12[(1]	−	−	−	−	−	−	

* iCEPS: induced pluripotent stem cell derived corneal epithelial cell sheet, † −: no abnormality, § +: abnormality.
#1: Subcutaneous nodule (nodule diameter: < 17 mm)
#2: Mediastinal tumour adhered to heart and lung
(1: Necropsied 112 days after administration. (2: Necropsied 77 days after administration. (3: Necropsied 63 days after administration. (4: Necropsied 56 days after administration. (5: Necropsied 49 days after administration. (6: Necropsied 42 days after administration. (7: Necropsied 35 days after administration

Table S1. ***Continued.***

Histopathology								
**Dose sample**	**Animal no.**	**Inspection site**						**Abnormal findings**
		**Brain**	**Lung**	**Liver**	**Kidney**	**Spleen**	**Injection site**	
iCEPS*	B1	−†	−	−	−	−	−	
	B2	−	−	−	−	−	−[#1]	
	B3	−	−	−	−	−	−[#1]	
	B4	−	−	−	−	−	−[#2]	
	B5	−	−	−	−	−	−[#1]	
	B6	−	−	−	−	−	−[#1]	
	B7	−	−	−	−	−	−[#1]	
	B8	−	−	−	−	−	−[#2]	
	B9	−	−	−	−	−	−[#1]	
	B10	−	−	−	−	−	−[#1]	
	B11	−	−	−	−	−	−[#1]	
	B12	−	−[#3]	−	−	−	−[#1]	
Positive control (iPSC 201B7 on MEF)	G1	−	−	−	−	−	+§[#4]	
	G2	−	−	−	−	−	+[#4]	
	G3	−	−	−	−	−	+[#4]	
	G4	−	−	−	−	−	+[#4]	
	G5	−	−	−	−	−	+[#4]	
	G6	−	−	−	−	−	+[#4]	
	G7	−	−	−	−	−	+[#4]	
	G8	−	−	−	−	−	+[#4]	
	G9	−	−	−	−	−	+[#4]	
	G10	−	−	−	−	−	+[#4]	
	G11	−	−	−	−	−	+[#4]	
	G12	−	−	−	−	−	+[#4]	
Negative control (Matrigel only)	R1	−	+[#5]	+[#5]	+[#5]	−	+[#5]	+[#6]
	R2	−	−	−	−	−	−[#2]	
	R3	−	−	−	−	−		
	R4	−	−	−	−	−		
	R5	−	−	−	−	−	−	
	R6	−	−	−	−	−	−[#2]	
	R7	−	−	−	−	−		
	R8	−	−	−	−	−		
	R9	−	−	−	−	−	−[#2]	
	R10	−	−	−	−	−		
	R11	−	−	−	−	−		
	R12	−	−	−	−	−		

* iCEPS: induced pluripotent stem cell derived corneal epithelial cell sheet, † −: no abnormality, § +: abnormality.
#1: Cyst formation, wall formed by several layers of epithelial cells
#2: Subcutaneous nodule, the remaining of Matrigell
#3: Small nodules in the vessel wall of non-human cell origin, no proliferative activity
#4: Tumourous proliferation of implanted cells
#5: Lymphoid tumour cells with proliferative activity
#6: Mediastinal tumour, compatible with thymoma

Table S2. Number of SNVs* and Indels† and CNVs§ detected in iCEPS¶

Cellular product name	Control	Assay	No. of SNVs and Indels			No. of CNVs	
			# CDS**, splicing regions	# COSMIC85††	# Census + Shibata list§§	# CNV	# Census + Shibata list
iCEPS-nonclinical	Cord blood cells of origin	Whole genome sequencing /SNP array (only for CNV)	0	0	−	0	−
		Exome sequencing	0	0	−	NT¶¶	NT

* SNVs: Single nucleotide variants, † Indels: insert or deletion, § CNVs: copy number variations, ¶ iCEPS: induced pluripotent stem cell derived corneal epithelial cell sheet, ** CDS: CoDing sequence, †† COSMIC85: COSMIC Cancer Gene Ceusus 85, §§ Census + Shibata list: the list of genes associated with tumourigenicity by the Japan Ministry of Health, Labor and Welfare, ¶¶ NT: not tested.

Table S3. Sequencing metrics of whole genome sequencings and exome sequencings

Whole genome sequencing					
**Cellular product name**	**Library prep kit**	**Sequencer**	**Sequence length**	**Sequencing reads**	**Depth of coverage**
iCEPS-nonclinical	Kapa Hyper Prep (PCR Free)	HiSeq2500	126bp, PE	2094.4M*	74·49
iCEPS-03	Kapa Hyper Prep (PCR Free)	HiSeq2500	126bp, PE	2117.9M	75·17
iCEPS-04	Kapa Hyper Prep (PCR Free)	HiSeq2500	126bp, PE	2148.9M	76·30
iCEPS-05	Kapa Hyper Prep (PCR Free)	NovaSeq6000	151bp, PE	2047.5M	71·65
iCEPS-06	Kapa Hyper Prep (PCR Free)	NovaSeq6000	151bp, PE	1952.0M	71·71

*M: million

Table S3. ***Continued***

		Exome sequencing			
**Cellular product name**	**Library prep kit**	**Sequencer**	**Paired end sequence length (bp)**	**Sequencing reads (M)**	**Depth of coverage**
iCEPS-nonclinical	SeqCap EZ	HiSeq2500	126	86·0	89·32
iCEPS-03	SeqCap EZ	HiSeq2500	126	113·1	111·75
iCEPS-04	SeqCap EZ	HiSeq2500	126	93·6	83·93
iCEPS-05	Twist Comprehensive Exome	NovaSeq6000	101	135·8	146·75
iCEPS-06	Twist Comprehensive Exome	NovaSeq6000	101	135·7	148·11

*M: million.

Table S4. Quality control tests and genome analysis in iCEPS* for transplantation

Patient	Cellular product name	Quality control test											Genome analysis	
		Sheet peeling	Total cell number (cells/ sheet)	Cell viability (%)	Cell purity (%)	Stem cell content	Corneal barrier function factors		iPSC marker (*LIN28A*) expression (copies/ RNA 50 ng)	Sterility test	Myco plasma	Endotoxin (EU†/mL)	Whole genome sequencing /SNP array	Exome sequencing
		SV§: peeling without damage	SV: $\geq 1.0 \times 10^6$	SV: ≥50	SV: ≥80	SV: +p63 cells at basal layer	SV: +ZO-1 sites between cells	SV: +Mucin-16 sites on surface	SV: ≤20	SV: negative	SV: negative	SV: <3	Census/ Shibata list¶ collation of mutations	(Same to the left)
1	iCEPS-03	○**	$6·7 \times 10^6$	86·5	99·3	+ ††	+	+	1	Negative	Negative	0·01909	-¶¶	-
2	iCEPS-04	○	$7·7 \times 10^6$	84·9	95·7	+	+	+	0·8	Negative	Negative	0·00972	-	-
3	iCEPS-05	○	$4·4 \times 10^6$	94·3	97·7	+	+	+	1·3	Negative	Negative	0·01365	-	-
4	iCEPS-06	○	$4·1 \times 10^6$	95·1	96·3	+	+	+	ND§§	Negative	Negative	0·02750	-	-

* iCEPS: induced pluripotent stem cell derived corneal epithelial cell sheet, † EU: endotoxin unit, § SV: standard value, ¶ Census/Shibata list: the list of genes associated with tumourigenicity by the Japan Ministry of Health, Labour and Welfare, ** ○: the result conformed to the standard value, †† +:positivity, §§ ND: not detectable as below limit, ¶¶ -:not found.

Table S5. Schedule and examination

Tests/observations \ Timing	Before enrollment	Day of transplantation			After transplantation (follow-up period)							
		Visit 1			Visit 2	Visit 3	Visit 4	Visit 5	Visit 6	Visit 7	Visit 8	Visit 9
	Screening	Day 0			Week 1 (Days 3–7)	Week 2 (Day 14)	Week 4 (Day 28)	Week 8 (Day 56)	Week 12 (Day 84)	Week 16 (Day 112)	Week 20 (Day 140)	Week 24 (Day 168)
**Acceptable range**	Within −56 days of enrollment					±3 days	±4 days	±7 days	±14 days	±14 days	±14 days	±14 days
		Before transplan-tation	During transplantation: After the removal of conjunctival tissue	During transplantation: Immediately after transplantation								
**Subject characteristics**	○											
**Slit-lamp microscopy and anterior segment photography**	○	○[*3]	○[*5]	○[*5]	○[*6]	○	○	○		○		○
**Anterior segment optical coherence tomography**	○	○[*3]				○	○	○		○		○
**Visual acuity test**	○	○[*3]				○	○	○		○		○
**Subjective symptoms**	○	○[*3]				○	○	○		○		○
**QOL**	○	○[*3]										
**Blood tests: Haematology**	○	○[*4]				○	○	☆	○	☆	☆	○
**Blood tests: Biochemistry**	○	○[*4]				○	○	☆	○	☆	☆	○
**Blood tests: Immunoserology**	○	○[*4]				○	○		○			○
**Blood tests: Tests for infectious diseases**	○											
**Blood tests: Tumour marker measurement[*1]**	○						○		○			○
**Blood tests: Trough level measurement**						☆	☆	☆	☆	☆	☆	☆
**Collection of blood samples for storage**		○[*4]										
**Urinalysis[*2]**	○	○[*3]										

Table S5. ***Continued***

Tests/observations \ Timing		After transplantation (follow-up period)									AM period[*7]
		Visit 10	Visit 11	Visit 12	Visit 13	Visit 14	Visit 15	Visit 16	Day of discontinuation		
		Week 28 (Day 196)	Week 32 (Day 224)	Week 36 (Day 252)	Week 40 (Day 280)	Week 44 (Day 308)	Week 48 (Day 336)	Week 52 (Day 364)			Week 104 (Day 728)
**Acceptable range**		±14 days	±14 days	±14 days	±14 days	±14 days	±14 days	±14 days	Within +7 days		±28 days
**Slit-lamp microscopy and anterior segment photography**			○		○			○	○		○
**Anterior segment optical coherence tomography**			○		○			○	○		○
**Visual acuity test**			○		○			○	○		○
**Subjective symptoms**			○		○			○	○		○
**QOL**								○	○		
**Blood tests**	**Hematology**	☆	☆	○	☆	☆	☆	○	○		
	**Biochemistry**	☆	☆	○	☆	☆	☆	○	○		
	**Immunoserology**			○				○	○		
	**Tests for infectious diseases**										
	**Tumor marker measurement**[*1]			○				○	○		
	**Trough level measurement**	☆	☆	☆	☆	☆	☆	☆	☆		

During the clinical study period, monitoring of systemic adverse events will be conducted as needed through blood tests and patient interviews.
Pathological diagnosis (biopsy tissue diagnosis) will be performed if an obvious abnormality is observed in the corneal epithelium on slit-lamp microscopy. If a neoplastic lesion is observed, an X-ray CT scan will be performed.
○: To be performed
☆: To be performed in subjects receiving immunosuppressive agents as specified in "6.2 Administration of Immunosuppressive Agents After iCEPS Transplantation"
*1: If values after iCEPS transplantation are higher than screening values and are abnormal, an oncologist will collaborate in the subject's care.
*2: Urine pregnancy tests will be performed for women of childbearing potential.
*3: To be performed within −4 days of iCEPS transplantation
*4: To be performed on the day of iCEPS transplantation
*5: Anterior segment photographs should be obtained by extracting frames from surgical videos.
*6: Only slit-lamp microscopy will be performed.
*7: A one-year Additional safety Monitoring (AM) period will be performed after the end of the 52-week follow-up period.

Table S6. Inclusion and exclusion criteria

Inclusion criteria

- Patients diagnosed with LSCD classification stage IIB, IIC, or III
- Cases one and two
  Patients whose HLA type is mismatched with CiRA_F-supplied iPS cell lines
- Cases three and four
  Will be determined on the number of cases of uncontrolled rejection in the first two cases
  1) Zero cases of rejection: HLA-mismatched patients like cases one and two
  2) One case of rejection: HLA-mismatched patients like cases one and two
  3) Two cases of rejection: HLA-matched patients
- Patients who are at least 20 years of age at the time of informed consent

Exclusion criteria

- Patients in whom drugs used in the clinical research are contraindicated
- Allergic to antibiotics (such as penicillin and streptomycin)
- Allergic to animals (cows, pigs, and rodents)
- Patients with infection meeting any of the following criteria:
  1) Active hepatitis B infection or a history of hepatitis B infection as determined by HBs antigen, HBs antibody, or HBc antibody testing
  2) Active hepatitis C infection as determined by HCV antibody or HCV-RNA testing
  3) Positive for HIV antibody
- History of malignant tumour within 5 years prior to screening
- Glaucoma with poorly controlled intraocular pressure
- Diabetes mellitus with poor glycemic control
- Pregnant women, lactating women, women who are possibly pregnant,
- Patients who have participated in another clinical study within 16 weeks
- Other patients who are not eligible for this clinical research

Table S7. HLA-haplotype in donors and patients with LSCD

Patient	HLA-A	HLA-B	HLA-C	HLA-DRB1	HLA-DQB1	HLA-DPB1
YZWJ (Donor)	24:02/−	52:01/−	12:02/−	15:02/−	06:01/−	09:01/-
1	02:01/02:06	35:01/46:01	01:02/03:04	09:01/12:01	03:01/03:03	03:01/14:01
2	02:01/33:03	35:01/44:03	03:03/14:03	04:03/08:03	03:02/06:01	02:01/02:02
3	26:01/31:01	40:02/54:01	01:02/03:03	04:05/09:01	03:03/04:01	05:01/09:01
4	02:07/24:02	40:02/44:03	14:03/15:02	08:02/14:54	04:02/05:03	05:01/06:01

Table S8. Adverse events up to the end of the 52-week follow-up period in the four patients

Series	Patient	Adverse events	Date of onset	Expression site	Severity level	Serious adverse event	Treatment for adverse events	Outcome of adverse events	Date of the outcome	Causality relationship between adverse events and the protocol	Cause of adverse events
1st series	1	Eye complication associated with device	16 Sep 2019	Treated eye	Mild	No	Wear contact lens	Recovered	16 Sep 2019	Possible	Surgery
		Eye pain	25 Jul 2020	Treated eye	Mild	No	Analgesics	Recovered	27 Jul 2020	Yes	Surgery
		Intraocular pressure increased	18 Nov 2019	Treated eye	Mild	No	Anti-glaucoma eye drop	Recovered	20 Jul 2020	Possible	Corticosteroids
		Corneal epithelium defect	10 Oct 2019	Treated eye	Mild	No	Use of minimal essential eyedrops	Unrecovered	20 Jul 2020	Possible	Side effect of eyedrop
		Cataract	29 Jan 2020	Treated eye	Mild	No	None	Unrecovered	20 Jul 2020	Possible	Corticosteroids
	2	Constipation	17 Dec 2019	Outside the eyes	Grade 1	No	Laxatives	Recovered	17 Dec 2019	No	Random coincidence
		Corneal herpes	20 Jan 2020	Treated eye	Mild	No	Anti-herpes drug	Recovered	30 Mar 2020	Possible	Corticosteroids and immunosuppressive agent
		Cataract aggravated	13 Apr 2020	Treated eye	Moderate	No	None	Unrecovered	16 Nov 2020	Possible	Corticosteroids
		Corneal epithelium defect	10 Feb 2020	Treated eye	Mild	No	Use of minimal essential eyedrops	Unrecovered	16 Nov 2020	Possible	Side effect of eyedrop
		Intraocular pressure increased	8 Jan 2020	Treated eye	Mild	No	Anti-glaucoma eye drop	Recovered	16 Nov 2020	Possible	Corticosteroids

Table S8. ***Continued***

Series	Patient	Adverse events	Date of onset	Expression site	Severity level	Serious adverse event	Treatment for adverse events	Outcome of adverse events	Date of the outcome	Causality relationship between adverse events and the protocol	Cause of adverse events
2nd series	3	Constipation	2 Aug 2020	Outside the eyes	Grade 1	No	Laxatives	Recovered	14 Aug 2020	No	Random coincidence
		Corneal epithelium Defect	7 Sep 2020	Treated eye	Mild	No	Anti-herpes drug	Recovered	14 Sep 2020	Possible	Mechanical trauma
		Corneal epithelium Defect	14 Dec 2020	Treated eye	Mild	No	Corticosteroids	Recovered	25 Jan 2021	No	Primary disease process
		Conjunctival Hyperemia	14 Dec 2020	Treated eye	Mild	No	Corticosteroids	Recovered	25 Jan 2021	No	Primary disease process
		Intraocular pressure Increased	12 Jan 2021	Treated eye	Mild	No	Anti-glaucoma eye drop	Recovered	28 Jun 2021	Possible	Corticosteroids
		Corneal epithelium Defect	15 Mar 2021	Treated eye	Mild	No	none	Recovered	12 Apr 2021	No	Primary disease process
		Corneal epithelium Defect	10 May2021	Treated eye	Mild	No	Corticosteroids	Recovered	31 May 2021	No	Primary disease process
		Corneal epithelium Defect	28 Jun 2021	Treated eye	Mild	No	Corticosteroids	Recovered	12 Jul 2021	No	Primary disease process
	4	Eye pain	24 Dec 2020	Treated eye	Mild	No	Analgesics	Recovered	25 Dec 2020	Possible	Surgery
		Constipation	19 Jan 2021	Outside the eyes	Grade 1	No	Laxatives	Recovered	20 Jan 2021	No	Random coincidence
		Conjunctival hyperemia	1 Feb 2021	Treated eye	Mild	No	Corticosteroids	Unrecovered	13 Dec 2021	No	Primary disease process
		Cushingoid	24 May 2021	Outside the Eyes	Grade 1	No	None	Unrecovered	13 Dec 2021	No	Corticosteroids
		Common cold	30 Aug 2021	Outside the eyes	Grade 1	No	None	Recovered	13 Sep 2021	No	Random coincidence
		Ocular hypertension	15 Feb 2021	Treated eye	Mild	No	Anti-glaucoma eye drop	Unrecovered	13 Dec 2021	No	Primary disease process
		White blood cell Increased	5 Jan 2021	Outside the Eyes	Grade 1	No	None	Recovered	13 Dec 2021	No	Random coincidence
		Decreased appetite	27 Sep 2021	Outside the eyes	Grade 1	No	None	Unrecovered	13 Dec 2021	No	random coincidence

Table S9. Adverse events recorded during the week 52-to104 additional safety monitoring period in the four patients

Series	Patient	Adverse events	Date of onset	Expression site	Severity level	Serious adverse event	Treatment for adverse events	Outcome of adverse events	Date of the outcome	Causality relationship between adverse events and the protocol	Cause of adverse events
1st series	1	Cataract	19 Jan 2019	Treated eye	Moderate	No	None	Unrecovered*	19 Jul 2021	Possible	Corticosteroids
		Eye pruritus	9 Nov 2020	Both eyes	Moderate	No	Converted anti-allergic eye drop to immunosuppressant eye drop	Recovered	7 Dec 2020	No	Exacerbation of pre-existing allergic conjunctivitis
		Corneal epithelium Defect	17 May 2021	Treated eye	Mild	No	Use of minimal essential eyedrops	Unrecovered	19 Jul 2021	Possible	Side effect of eyedrop
	2	Cataract aggravated	13 Apr 2020	Treated eye	Moderate	No	None	Unrecovered†	22 Nov 2021	Possible	Corticosteroids
		Corneal epithelium Defect	25 Oct 2021	Treated eye	Mild	No	Use of minimal essential eyedrops	Unrecovered	20 Dec 2021	Possible	Side effect of eyedrop
2nd series	3	Cataract	24 Aug 2021	Treated eye	Moderate	No	Cataract Surgery	Recovered	10 Mar 2022	Possible	Corticosteroids
		Corneal epithelium Defect	20 Jun 2022	Treated eye	Mild	No	Use of minimal essential eyedrops	Unrecovered	11 Jul 2022	Possible	Side effect of eyedrop
	4	Cataract	6 Jun 2022	Treated eye	Mild	No	None	Unrecovered	16 Jan 2023	Possible	Corticosteroids
		Intraocular pressure Increased	4 Jul 2022	Treated eye	Mild	No	Anti-glaucoma drugs and change to lower corticosteroid eyedrop	Recovered	22 Aug 2022	Possible	Corticosteroids
		Corneal epithelium Defect	3 Oct 2022	Treated eye	Mild	No	Use of minimal essential eyedrops	Unrecovered	16 Jan 2023	Possible	Side effect of eyedrop

* Cataract surgery performed on 6 Jan, 2022 restored visual acuity from 1·10 to 0·22, 130 weeks after iCEPS transplantation.
† Cataract surgery performed on 27 Jan, 2022 restored visual acuity from 2·00 to 1·00, 118 weeks after iCEPS transplantation.

Table S10. The LSCD* stage following iCEPS transplantation

Series	Patient	Preoperative	2W	4W	8W	16W	24W	32W	40W	52W	104W
1st Series	1	III	IA	IA	IA	IA	IA	IA	IA	IA	IA
	2	III	IA	IA	IA	IA	IA	IA	IA	IA	IA
2nd Series	3	IIB	IA	IA	IA	IA	IA	IA	IA	IA	IA
	4	III	IA	IA	IA	IA	IA	IA	IB	IIB	III

*LSCD: limbal stem cell deficiency

Table S11. Corrected distance visual acuity following iCEPS transplantation (Decimal visual acuity converted to LogMAR)

Series	Patient	Preoperative	2W	4W	8W	16W	24W	32W	40W	52W	104W
1st Series	1	1·52	0·30	0·30	0·22	0·14	0·30	0·40	0·22	0·52	1·10*
	2	2·00	1·04	0·70	0·82	0·52	1·00	1·00	1·04	0·82	2·00†
2nd Series	3	0·82	0·10	0·16	0·30	0·10	0·22	0·16	0·22	0·16	0·15
	4	1·68	0·82	0·82	0·82	0·70	0·52	0·70	1·04	1·40	2·00

* Cataract surgery performed on 6 Jan, 2022 restored visual acuity from 1·10 to 0·22, 130 weeks after iCEPS transplantation.
† Cataract surgery performed on 27 Jan, 2022 restored visual acuity from 2·00 to 1·00, 118 weeks after iCEPS transplantation.

Table S12. Early treatment diabetic retinopathy study total score following iCEPS transplantation

Series	Patient	Preoperative	2W	4W	8W	16W	24W	32W	40W	52W	104W
1st Series	1	17	61	63	70	60	52	57	66	56	21
	2	0	28	35	56	58	39	30	47	54	0
2nd Series	3	34	63	53	51	61	60	58	54	53	62
	4	0	37	29	33	42	35	45	33	0	0

Table S13. Early treatment diabetic retinopathy study visual acuity following iCEPS transplantation

Series	Patient	Preoperative	2W	4W	8W	16W	24W	32W	40W	52W	104W
1st Series	1	1·36	0·48	0·44	0·30	0·50	0·66	0·56	0·38	0·58	1.28
	2	3·00	1·14	1·00	0·58	0·54	0·92	1·10	0·76	0·62	3.0
2nd Series	3	1·02	0·44	0·64	0·68	0·48	0·50	0·54	0·62	0·64	0.46
	4	3·00	0·96	1·12	1·04	0·86	1·00	0·80	1·04	3·00	2.0

Table S14. Degree of corneal opacification following iCEPS transplantation

Series	Patient	Section	Preoperative	2W	4W	8W	16W	24W	32W	40W	52W	104W
1st Series	1	1	3	0	0	0	0	0	0	0	1	1
		2	3	0	0	0	0	0	0	0	1	1
		3	3	0	0	0	0	0	0	0	1	1
		4	3	0	0	0	0	0	0	0	0	0
		5	3	0	0	0	0	0	0	0	0	0
		6	2	0	0	0	0	0	0	0	0	0
		7	2	0	0	0	0	0	0	0	0	0
		8	3	0	0	0	0	0	0	0	1	1
		Average	2·8	0·0	0·0	0·0	0·0	0·0	0·0	0·0	0·5	0·5
	2	1	3	1	1	1	1	1	1	1	1	0
		2	3	1	1	1	1	1	1	1	1	0
		3	3	1	0	1	1	1	1	1	1	0
		4	3	1	1	1	1	1	1	1	1	0
		5	3	1	1	1	1	1	1	1	1	0
		6	3	1	1	1	1	1	1	1	1	0
		7	3	1	1	1	1	1	1	1	1	0
		8	3	1	1	1	1	1	1	1	1	0
		Average	3·0	1·0	0·9	1·0	1·0	1·0	1·0	1·0	1·0	0

Table S14. *Continued*

Series	Patient	Section	Preoperative	2W	4W	8W	16W	24W	32W	40W	52W	104W
2nd Series	3	1	1	0	0	0	0	1	1	1	1	0
		2	1	0	0	0	0	1	1	1	1	1
		3	1	0	0	0	0	1	1	1	1	1
		4	1	0	0	0	0	0	0	0	0	0
		5	1	0	0	0	0	0	0	0	0	0
		6	1	0	0	0	0	0	0	0	0	0
		7	1	0	0	0	0	0	0	0	0	0
		8	1	0	0	0	0	0	0	0	0	0
		Average	1·0	0·0	0·0	0·0	0·0	0·4	0·4	0·4	0·4	0·3
	4	1	3	1	2	1	1	1	1	1	2	2
		2	3	1	2	1	1	1	1	1	2	2
		3	3	1	2	1	1	1	1	1	2	2
		4	3	1	2	1	1	1	1	1	1	2
		5	3	1	2	1	1	1	1	1	1	2
		6	3	1	2	1	1	1	1	1	1	2
		7	3	1	2	1	1	1	1	1	2	2
		8	3	1	2	1	1	1	1	1	2	2
		Average	3·0	1·0	2·0	1·0	1·0	1·0	1·0	1·0	1·6	2·0

Table S15. Corneal thickness (μm) following iCEPS transplantation

Series	Patient	Section	Preoperative	2W	4W	8W	16W	24W	32W	40W	52W	104W
1st Series	1	1	661	490	434	430	460	426	437	421	431	443
		2	644	483	413	413	434	416	433	423	441	450
		3	603	456	374	384	402	385	402	372	406	421
		4	529	431	348	364	389	356	370	347	372	384
		5	535	426	352	385	383	356	359	339	348	371
		6	556	455	388	415	428	373	383	364	363	388
		7	622	493	424	435	472	419	425	407	390	410
		8	665	504	454	444	482	445	443	432	424	434
		Centre	576	447	387	394	419	367	383	365	374	398
	2	1	646	508	498	513	494	504	535	517	505	426
		2	636	506	487	498	513	482	571	503	501	417
		3	684	478	462	481	493	422	472	464	470	415
		4	661	498	442	470	467	424	461	382	423	429
		5	656	497	453	476	469	399	462	427	442	464
		6	663	488	449	476	447	400	440	403	441	449
		7	639	474	460	494	445	434	458	451	447	453
		8	646	500	490	514	476	486	525	508	486	458
		Centre	643	466	437	465	459	389	438	406	409	386

Table S15. ***Continued***

Series	Patient	Section	Preoperative	2W	4W	8W	16W	24W	32W	40W	52W	104W
2nd Series	3	1	445	460	437	425	447	445	449	445	443	408
		2	442	462	437	424	445	432	442	443	452	401
		3	423	458	448	440	447	441	444	457	453	402
		4	420	456	462	432	443	450	458	469	450	397
		5	419	455	465	443	431	433	447	453	428	376
		6	425	457	457	436	444	446	448	459	436	411
		7	423	454	451	444	453	456	453	452	443	404
		8	438	461	450	449	459	454	461	451	445	433
		Centre	422	453	447	437	435	426	442	449	438	410
	4	1	596	419	446	472	429	422	456	426	448	373
		2	638	438	456	453	442	427	432	448	482	386
		3	621	436	448	435	428	414	435	443	481	475
		4	611	432	439	439	411	390	407	424	462	540
		5	546	413	427	404	396	375	398	387	443	469
		6	557	412	435	399	390	372	403	400	436	461
		7	536	401	422	414	384	419	411	407	425	448
		8	540	386	405	422	387	420	434	398	407	380
		Centre	561	421	434	437	420	396	400	413	443	446

Table S16. Transparency of the corneal epithelium following iCEPS transplantation

Series	Patient	Section	Preoperative	2W	4W	8W	16W	24W	32W	40W	52W
1st Series	1	1	0·78	0·23	0·14	0·18	0·26	0·22	0·22	0·15	0·13
		2	1·22	0·30	0·15	0·19	0·25	0·24	0·26	0·19	0·25
		3	0·60	0·15	0·11	0·13	0·16	0·19	0·20	0·12	0·07
		4	0·62	0·17	0·10	0·16	0·20	0·22	0·22	0·14	0·14
		5	0·49	0·15	0·10	0·16	0·16	0·17	0·18	0·12	0·11
		6	0·80	0·21	0·17	0·24	0·24	0·27	0·28	0·20	0·15
		7	0·70	0·20	0·16	0·17	0·24	0·23	0·22	0·18	0·13
		8	1·06	0·40	0·22	0·24	0·34	0·34	0·32	0·26	0·19
		Average	0·78	0·23	0·14	0·18	0·23	0·24	0·24	0·17	0·15
	2	1	0·91	0·12	0·12	0·16	0·15	0·19	0·18	0·17	0·09
		2	1·43	0·17	0·19	0·20	0·21	0·27	0·27	0·25	0·18
		3	1·15	0·14	0·16	0·20	0·17	0·16	0·18	0·13	0·09
		4	1·35	0·26	0·21	0·25	0·20	0·22	0·19	0·16	0·11
		5	0·98	0·17	0·19	0·19	0·18	0·15	0·16	0·14	0·09
		6	1·48	0·23	0·29	0·28	0·26	0·20	0·21	0·19	0·16
		7	1·00	0·19	0·21	0·21	0·20	0·16	0·23	0·11	0·13
		8	1·28	0·21	0·22	0·22	0·21	0·25	0·29	0·26	0·17
		Average	1·20	0·19	0·20	0·21	0·20	0·20	0·21	0·18	0·13

Table S16. ***Continued***

Series	Patient	Section	Preoperative	2W	4W	8W	16W	24W	32W	40W	52W
2nd Series	3	1	0·22	0·20	0·14	0·17	0·16	0·17	0·16	0·16	0·20
		2	0·25	0·24	0·18	0·20	0·21	0·18	0·17	0·17	0·25
		3	0·17	0·16	0·15	0·16	0·15	0·11	0·12	0·13	0·19
		4	0·27	0·18	0·18	0·19	0·19	0·14	0·18	0·17	0·22
		5	0·18	0·14	0·17	0·22	0·15	0·14	0·16	0·14	0·16
		6	0·27	0·21	0·25	0·29	0·25	0·22	0·26	0·23	0·25
		7	0·22	0·19	0·19	0·22	0·21	0·18	0·20	0·21	0·21
		8	0·29	0·27	0·24	0·31	0·26	0·26	0·29	0·28	0·28
		Average	0·23	0·20	0·19	0·22	0·20	0·18	0·19	0·19	0·22
	4	1	0·60	0·17	0·12	0·19	0·10	0·14	0·22	0·22	0·20
		2	0·95	0·18	0·17	0·17	0·13	0·27	0·27	0·36	0·35
		3	0·62	0·17	0·11	0·11	0·14	0·19	0·15	0·16	0·29
		4	0·92	0·32	0·16	0·16	0·14	0·15	0·22	0·23	0·35
		5	0·53	0·21	0·11	0·07	0·08	0·10	0·15	0·12	0·24
		6	0·74	0·25	0·20	0·09	0·13	0·27	0·19	0·25	0·35
		7	0·50	0·20	0·18	0·11	0·16	0·30	0·19	0·26	0·24
		8	0·74	0·28	0·15	0·20	0·16	0·35	0·33	0·33	0·30
		Average	0·70	0·22	0·15	0·14	0·13	0·22	0·22	0·24	0·29

Table S17. Corneal epithelial thickness (μm) following iCEPS transplantation

Series	Patient	Section	Preoperative	2W	4W	8W	16W	24W	32W	40W	52W
1st Series	1	1	162	60	34	64	73	56	52	56	48
		2	180	48	30	26	38	26	38	30	30
		3	175	34	38	22	38	22	34	30	38
		4	151	30	30	22	30	26	26	26	26
		5	101	38	34	56	30	22	26	18	30
		6	108	38	38	48	30	38	22	22	30
		7	166	56	34	38	86	38	52	38	30
		8	190	43	69	34	34	34	30	26	30
		Centre	139	42	30	38	34	38	34	34	34
	2	1	160	38	43	22	34	18	30	30	26
		2	185	47	43	38	47	34	26	22	18
		3	247	43	43	51	64	8	30	26	26
		4	179	47	38	51	56	43	38	21	38
		5	145	38	30	34	38	21	30	34	34
		6	180	47	47	47	43	34	26	13	25
		7	180	34	38	60	30	47	17	21	13
		8	156	30	38	38	56	26	30	18	13
		Centre	173	46	38	46	46	21	25	17	21

Table S17. ***Continued***

Series	Patient	Section	Preoperative	2W	4W	8W	16W	24W	32W	40W	52W
2nd Series	3	1	26	43	22	30	26	26	26	26	18
		2	38	38	26	34	38	30	26	18	38
		3	30	30	22	22	30	26	26	22	30
		4	13	51	26	26	26	34	22	22	26
		5	13	30	38	34	22	43	30	18	18
		6	38	8	18	26	30	48	30	18	26
		7	34	22	22	30	30	30	30	18	4
		8	18	30	26	43	22	26	34	13	18
		Centre	21	34	25	34	30	25	30	21	21
	4	1	107	30	34	30	38	43	18	52	70
		2	150	18	17	22	22	26	21	51	60
		3	94	22	30	26	22	22	17	51	60
		4	124	22	22	34	22	17	13	38	56
		5	94	43	26	22	18	26	4	51	47
		6	128	13	30	22	22	26	8	52	34
		7	107	13	18	43	22	30	13	52	69
		8	115	18	13	8	13	13	22	38	52
		Centre	106	25	38	13	30	34	13	30	46

Table S18. Severity of corneal epithelial defect following iCEPS transplantation

Series	Patient	Preoperative	2W	4W	8W	16W	24W	32W	40W	52W	104W
1st Series	1	0	0	0	0	0	0	0	0	0	1
	2	1	0	0	1	1	0	0	0	0	1
2nd Series	3	1	0	1	1	1	1	1	1	0	1
	4	1	1	1	0	1	1	1	1	1	1

Table S19. Degree of corneal neovascularisation following iCEPS transplantation

Series	Patient	Section	Preoperative	2W	4W	8W	16W	24W	32W	40W	52W	104W
1st Series	1	1	3	2	2	2	2	2	2	2	2	2
		2	3	1	1	1	1	1	1	1	1	1
		3	3	1	1	1	1	1	1	1	1	1
		4	3	1	1	1	1	1	1	1	1	1
		5	3	0	0	0	0	0	1	1	1	0
		6	3	1	1	1	1	0	1	1	1	1
		7	3	1	1	1	1	1	1	1	1	1
		8	3	1	1	1	1	1	1	1	1	1
		Average	3·0	1·0	1·0	1·0	1·0	0·9	1·1	1·1	1·1	1·0
	2	1	3	0	1	1	1	1	1	1	1	1
		2	3	0	0	0	1	1	1	1	1	1
		3	3	0	0	1	0	0	1	1	1	1
		4	3	0	1	1	1	1	1	1	1	1
		5	3	0	1	1	1	1	1	1	1	1
		6	3	1	0	1	1	1	1	1	1	1
		7	3	1	1	1	1	1	1	1	1	1
		8	3	0	1	1	1	1	1	1	1	1
		Average	3·0	0·3	0·6	0·9	0·9	0·9	1·0	1·0	1·0	1·0

Table S19. ***Continued***

Series	Patient	Section	Preoperative	2W	4W	8W	16W	24W	32W	40W	52W	104W
2nd Series	3	1	2	2	2	2	2	2	2	2	2	2
		2	1	1	1	1	1	1	1	1	1	2
		3	1	1	1	1	1	1	1	1	2	2
		4	2	1	1	1	1	1	1	1	2	2
		5	2	1	1	1	1	1	1	2	2	2
		6	1	1	1	1	1	1	1	2	2	2
		7	1	1	1	1	1	1	1	1	1	1
		8	2	1	2	2	1	2	2	2	2	1
		Average	1·5	1·1	1·3	1·3	1·1	1·3	1·3	1·5	1·8	1·8
	4	1	3	0	1	1	1	2	2	2	3	3
		2	3	0	1	1	1	2	2	3	3	3
		3	3	0	0	1	1	1	2	3	3	3
		4	3	1	1	1	1	1	2	2	3	3
		5	3	1	1	1	1	1	2	2	3	3
		6	3	1	1	1	1	2	2	2	3	3
		7	3	1	1	1	1	3	3	3	3	3
		8	3	0	1	1	1	1	3	3	3	3
		Average	3·0	0·5	0·9	1·0	1·0	1·6	2·3	2·5	3·0	3·0

Table S20. Degree of subepithelial corneal neovascularisation following iCEPS transplantation

Series	Patient	Section	Preoperative	2W	4W	8W	16W	24W	32W	40W	52W	104W
1st Series	1	1	3	1	1	1	1	1	1	1	1	1
		2	3	1	1	1	1	1	1	1	1	1
		3	3	0	1	1	1	0	1	1	1	1
		4	3	0	1	1	1	0	0	0	1	1
		5	3	0	0	0	0	0	1	1	1	0
		6	3	0	1	1	1	0	1	1	1	1
		7	3	0	1	1	1	1	1	1	1	1
		8	3	1	1	1	1	1	1	1	1	1
		Average	3·0	0·4	0·9	0·9	0·9	0·5	0·9	0·9	1·0	0·9
	2	1	3	0	1	1	1	1	1	1	1	1
		2	3	0	0	0	1	1	1	1	1	1
		3	3	0	0	0	0	0	0	1	1	1
		4	3	0	0	1	0	1	1	1	1	1
		5	3	0	1	0	1	1	1	1	1	1
		6	3	0	0	1	1	1	1	1	1	1
		7	3	0	0	1	1	1	1	1	1	1
		8	3	0	1	1	1	1	1	1	1	1
		Average	3·0	0·0	0·4	0·6	0·8	0·9	0·9	1·0	1·0	1·0

Table S20. ***Continued***

Series	Patient	Section	Preoperative	2W	4W	8W	16W	24W	32W	40W	52W	104W
2nd Series	3	1	1	1	1	1	1	2	2	1	1	1
		2	1	1	1	1	1	1	1	1	1	1
		3	1	1	1	1	1	1	1	1	1	1
		4	2	1	1	1	1	1	1	1	1	1
		5	2	1	1	1	1	1	1	2	2	1
		6	1	1	1	1	1	1	1	2	2	1
		7	1	1	1	1	1	1	1	1	1	1
		8	2	1	1	1	1	2	2	1	1	1
		Average	1·4	1·0	1·0	1·0	1·0	1·3	1·3	1·3	1·3	1·0
	4	1	3	0	1	1	1	1	1	2	3	3
		2	3	0	1	1	1	1	1	1	3	3
		3	3	0	0	1	1	1	1	2	3	3
		4	3	1	1	1	1	1	2	2	3	3
		5	3	1	1	1	1	1	1	2	3	3
		6	3	1	1	1	1	2	2	2	3	3
		7	3	0	1	1	1	3	3	3	3	3
		8	3	0	1	1	1	1	3	3	3	3
		Average	3·0	0·4	0·9	1·0	1·0	1·4	1·8	2·1	3·0	3·0

Table S21. Degree of symblepharon following iCEPS transplantation

Series	Patient	Preoperative	2W	4W	8W	16W	24W	32W	40W	52W	104W
1st Series	1	0	0	0	0	0	0	0	0	0	0
	2	1	0	0	0	0	1	1	1	1	0
2nd Series	3	0	0	0	0	0	0	0	0	0	0
	4	0	0	0	0	0	0	0	0	0	0

Table S22. Ocular pain following iCEPS transplantation

Series	Patient	Preoperative	2W	4W	8W	16W	24W	32W	40W	52W	104W
1st Series	1	0	0	0	0	0	0	0	0	0	0
	2	0	0	0	1	0	0	1	0	1	1
2nd Series	3	0	0	0	0	0	0	0	0	0	0
	4	1	0	1	1	1	1	0	1	1	2

Table S23. Foreign body sensation following iCEPS transplantation

Series	Patient	Preoperative	2W	4W	8W	16W	24W	32W	40W	52W	104W
1st Series	1	1	0	1	1	1	1	1	1	1	1
	2	1	0	1	1	1	1	1	1	1	1
2nd Series	3	0	0	0	0	0	0	0	0	0	0
	4	2	3	1	2	2	2	2	2	2	3

Table S24. Lacrimation following iCEPS transplantation

Series	Patient	Preoperative	2W	4W	8W	16W	24W	32W	40W	52W	104W
1st Series	1	1	2	1	0	1	0	0	0	0	1
	2	1	0	1	1	0	0	0	0	0	0
2nd Series	3	0	1	0	0	0	0	0	1	1	0
	4	0	1	1	0	0	1	1	1	1	1

Table S25. Photophobia following iCEPS transplantation

Series	Patient	Preoperative	2W	4W	8W	16W	24W	32W	40W	52W	104W
1st Series	1	4	3	1	0	1	0	1	1	1	2
	2	1	0	1	2	1	0	2	1	1	0
2nd Series	3	1	1	0	0	1	1	1	1	1	1
	4	4	4	3	3	4	4	4	4	4	4

Table S26. Dryness following iCEPS transplantation

Series	Patient	Preoperative	2W	4W	8W	16W	24W	32W	40W	52W	104W
1st Series	1	1	1	1	0	2	2	1	1	2	1
	2	1	1	0	1	1	1	0	1	1	1
2nd Series	3	0	0	0	1	0	0	0	0	0	0
	4	4	0	0	2	2	2	2	2	2	2

Table S27. Discomfort following iCEPS transplantation

Series	Patient	Preoperative	2W	4W	8W	16W	24W	32W	40W	52W	104W
1st Series	1	1	0	0	1	1	1	1	0	1	0
	2	2	0	1	1	1	1	1	1	1	1
2nd Series	3	1	0	0	1	0	0	0	0	0	0
	4	2	3	2	1	2	2	1	2	2	2

Table S28. Quality of life score using NEI VFQ-25 following iCEPS transplantation

Series	Patient	Preoperative	52W
1st Series	1	52·1	82·2
	2	26·5	29·7
2nd Series	3	59·7	73·1
	4	65·9	39·9

Table S29. National Eye Institute Visual Function Questionnaire-25 subscale scores following iCEPS transplantation

Series	Patient	Time	General health	General vision	Ocular pain	Near vision	Distance vision	Vision specific social function	Vision specific mental health	Vision specific role limitation	Vision specific dependency	Driving	Color vision	Peripheral vision
1st Series	1	preoperative	50*	40	87·5	62·5**	50	62·5	43·8	25	50	*	75	25
		52W	50*	80	87·5	62·5**	75	87·5	75	87·5	91·7	*	100	75
	2	preoperative	50*	0	87·5	0**	8·3	0	31·3	12·5	0	0*	50	75
		52W	75*	60	75	25	16·7	0	37·5	25	8·3	0*	0	50
2nd Series	3	preoperative	75*	60	100	66·7	50	50	50	37·5	58·3	50*	75	50
		52W	50*	60	87·5	50	83·3	75	62·5	62·5	75	37·5*	100	75
	4	preoperative	50*	40	62·5	50**	50	75	68·8	87·5	75	0*	100	50
		52W	50*	20	37·5	25**	33·3	50	50	50	33·3	0*	75	25

* Not used to calculate QOL scores·

** Since the response indicates that the activity is not performed for reasons unrelated to visual acuity, it is treated as a missing value according to the "NEI VFQ-25 Japanese version (v1·4) Instructions for Use" guidelines·

Table S30. Immunological tests in patients with LSCD–corneal epithelium-specific antibody tests

Series	Patient	Primary antibody		Preoperative	2W	4W	12W	24W	36W	52W
1st Series	1	Preoperative patient serum (as baseline)	Sample 1	-	990	990	990	846	846	846
			Sample 2	-	930	930	930	828	828	828
			Sample 3	-	951	951	951	853	853	853
			Sample 4	-	938	938	938	836	836	836
		Patient serum	Sample 1	880	958	911	959	822	801	799
			Sample 2	897	879	895	920	801	795	809
			Sample 3	852	937	894	922	829	836	826
			Sample 4	901	907	868	933	855	794	836
		Healthy donor serum (Control)	Sample 1	803	926	926	926	803	803	803
			Sample 2	828	915	915	915	828	828	828
			Sample 3	841	877	877	877	841	841	841
			Sample 4	833	867	867	867	833	833	833
		Negative control (without primary antibody)		608	606	606	606	608	608	608
		Blank		585	556	556	556	585	585	585
		Keratin-14		5172	2949	2949	2949	5172	5172	5172
		Isotype control for Keratin-14 (Rabbit IgG)		621	604	604	604	621	621	621

Table S30. ***Continued***

Series	Patient	Primary antibody		Preoperative	2W	4W	12W	24W	36W	52W
1st Series	2	Preoperative patient serum (as baseline)	Sample 1	-	1197	1197	1061	1061	1061	1061
			Sample 2	-	1095	1095	1008	1008	1008	1008
			Sample 3	-	1106	1106	1014	1014	1014	1014
			Sample 4	-	1108	1108	1003	1003	1003	1003
		Patient serum	Sample 1	904	974	879	1075	998	886	1025
			Sample 2	909	933	939	1064	1007	841	1049
			Sample 3	947	919	905	1043	998	853	1113
			Sample 4	901	908	883	1007	1014	866	999
		Healthy donor serum (Control)	Sample 1	803	926	926	820	820	820	820
			Sample 2	828	915	915	834	834	834	834
			Sample 3	841	877	877	855	855	855	855
			Sample 4	833	867	867	825	825	825	825
		Negative control (without primary antibody)		608	606	606	601	601	601	601
		Blank		585	556	556	567	567	567	567
		Keratin-14		5172	2949	2949	5565	5565	5565	5565
		Isotype control for Keratin-14 (Rabbit IgG)		621	604	604	608	608	608	608

Table S30. ***Continued***

Series	Patient	Primary antibody		Preoperative	2W	4W	12W	24W	36W	52W
2nd Series	3	Preoperative patient serum (as baseline)	Sample 1	-	807	807	807	807	807	807
			Sample 2	-	816	816	816	816	816	816
			Sample 3	-	848	848	848	848	848	848
			Sample 4	-	803	803	803	803	803	803
		Patient serum	Sample 1	814	769	774	807	796	827	805
			Sample 2	878	757	763	782	775	797	824
			Sample 3	896	791	762	803	807	895	786
			Sample 4	858	794	767	773	805	855	779
		Healthy donor serum (Control)	Sample 1	820	789	789	789	789	789	789
			Sample 2	834	827	827	827	827	827	827
			Sample 3	855	832	832	832	832	832	832
			Sample 4	825	792	792	792	792	792	792
		Negative control (without primary antibody)		601	633	633	633	633	633	633
		Blank		567	593	593	593	593	593	593
		Keratin-14		5565	4871	4871	4871	4871	4871	4871
		Isotype control for Keratin-14 (Rabbit IgG)		608	602	602	602	602	602	602

Table S30. ***Continued***

Series	Patient	Primary antibody		Preoperative	2W	4W	12W	24W	36W	52W
2nd Series	4	Preoperative patient serum (as baseline)	Sample 1	-	901	901	901	901	901	1016
			Sample 2	-	894	894	894	894	894	1063
			Sample 3	-	908	908	908	908	908	1038
			Sample 4	-	899	899	899	899	899	1035
		Patient serum	Sample 1	901	900	866	831	779	772	987
			Sample 2	919	880	836	835	795	830	1019
			Sample 3	894	899	856	880	766	858	1009
			Sample 4	911	841	806	907	773	789	987
		Healthy donor serum (Control)	Sample 1	878	878	878	878	878	878	1031
			Sample 2	892	892	892	892	892	892	1061
			Sample 3	875	875	875	875	875	875	1050
			Sample 4	902	902	902	902	902	902	1054
		Negative control (without primary antibody)		607	607	607	607	607	607	665
		Blank		550	550	550	550	550	550	647
		Keratin-14		5140	5140	5140	5140	5140	5140	4563
		Isotype control for Keratin-14 (Rabbit IgG)		637	637	637	637	637	637	653

Table S31. Immunological tests in patients with LSCD–MLR

Series	Patient	Sample		Preoperative	2W	4W	12W	24W	36W	52W
1st Series	1	CD4-Ki67 (%)	PBMC	4·3	8·5*	7·3*	6·4*	17·3	16·9	14·2
			PBMC+iCEPS	7·7	21·4	13·4	19·2	24·6	12·7	11·3
			PBMC+B cell	17·1	34	28·6	27	20·3	49	23·2
		CD8-Ki67 (%)	PBMC	3·6	5·3*	3·6*	5·2*	7	5·5	11·2
			PBMC+iCEPS	2·8	7·7	7·5	6·1	10	6·4	7·4
			PBMC+B cell	26·5	38	29·4	22·9	31·8	46·1	32·6
		CD11b-Ki67 (%)	PBMC	3·1	7·8*	4·7*	6·9*	14·6	17·4	18
			PBMC+iCEPS	10·7	14·3	10·7	16	19·2	12·4	15·3
			PBMC+B cell	8·8	24·2	13·5	12·6	18·3	31·7	22·6
		CD56-Ki67 (%)	PBMC	0·6	2·8*	0·8*	2·2*	3·6	6·5	7·9
			PBMC+iCEPS	1·2	2	1·1	3·8	3·7	3·8	8
			PBMC+B cell	6·5	8	2·9	4·4	8·7	14·7	13·2
		IFN-γ (ng/mL)	PBMC	472	1329*	2014*	1999*	787	698	573
			PBMC+iCEPS	320	1110	963	735	890	613	1617
			PBMC+B cell	14648	3211	10462	14897	23439	28056	30838

*Value at half the culture medium

Table S31. ***Continued***

Series	Patient	Sample		Preoperative	2W	4W	12W	24W	36W	52W
1st Series	2	CD4-Ki67 (%)	PBMC	22·6	7·5	4·8	9·9	17·4	-**	24·3
			PBMC+iCEPS	35·7	16·9	18·5	10·6	28·6	-**	31·6
			PBMC+B cell	29·2	26·5	29·1	17·8	44·9	-**	47·4
		CD8-Ki67 (%)	PBMC	22	7·3	2·3	7·6	16·1	-**	15·5
			PBMC+iCEPS	20·9	11·7	12·7	9·2	15·8	-**	19·7
			PBMC+B cell	38·3	27·5	29·6	18·8	44·2	-**	44
		CD11b-Ki67 (%)	PBMC	17·3	7·9	2·7	10·6	19·7	-**	17·6
			PBMC+iCEPS	17·4	14·6	10·2	8·9	17·9	-**	24·9
			PBMC+B cell	16·5	21·6	22·9	13·1	46·5	-**	44·3
		CD56-Ki67 (%)	PBMC	12	2·7	0·7	4·4	6·8	-**	5·1
			PBMC+iCEPS	7·2	2·8	2·1	2·9	4·6	-**	7·7
			PBMC+B cell	19·3	22	32·7	18·7	43·2	-**	45·1
		IFN-γ (ng/mL)	PBMC	3334	220	661	682	451	-**	520
			PBMC+iCEPS	1223	190	555	688	401	-**	855
			PBMC+B cell	38916	2240	19438	34557	19297	-**	18568

**not conducted

Table S31. ***Continued***

Series	Patient	Sample		Preoperative	2W	4W	12W	24W	36W	52W
2nd Series	3	CD4-Ki67 (%)	PBMC	4·9	-**	15·3	15·2	20·4	11·5	12·6
			PBMC+iCEPS	10·1	-**	23·8	19·6	29·8	26·9	20·4
			PBMC+B cell	35·1	-**	47·6	49·4	19·4	28·1	34·9
		CD8-Ki67 (%)	PBMC	2·7	-**	8·8	6·8	6·6	5·3	10
			PBMC+iCEPS	3·7	-**	6·6	4·4	6·4	7·5	5
			PBMC+B cell	29·4	-**	47·1	36·4	35	23·8	28·3
		CD11b-Ki67 (%)	PBMC	6·9	-**	13	14·9	12·1	13·4	14·9
			PBMC+iCEPS	6·7	-**	18·1	17·9	24·8	24·6	11·1
			PBMC+B cell	30·9	-**	34·5	39·8	33·5	35·7	27·5
		CD56-Ki67 (%)	PBMC	3·5	-**	3·3	5·1	2·4	5·6	6·1
			PBMC+iCEPS	1·7	-**	2·1	3·4	1·4	5·3	1·3
			PBMC+B cell	29·7	-**	30·6	38·6	29·1	37·8	31·9
		IFN-γ (ng/mL)	PBMC	109	-**	255	198	313	137	342
			PBMC+iCEPS	131	-**	244	320	454	145	450
			PBMC+B cell	3447	-**	782	3895	2017	2170	2721

**not conducted

Table S31. ***Continued***

Series	Patient	Sample		Preoperative	2W	4W	12W	24W	36W	52W
2nd Series	4	CD4-Ki67 (%)	PBMC	16·2	13·6	10·9	14·6	10·2	14·4	13·7
			PBMC+iCEPS	25·6	25·3	18·6	28	26·2	17·5	18·6
			PBMC+B cell	27·9	30·4	29·8	23·1	28·1	31·9	26·5
		CD8-Ki67 (%)	PBMC	6·7	6·6	3·9	6·9	7·3	4·3	4·7
			PBMC+iCEPS	8·4	7·4	7·5	7·5	9	4·1	5·6
			PBMC+B cell	44·4	35·8	42·1	45·9	48·4	36·4	41·6
		CD11b-Ki67 (%)	PBMC	12·6	11·7	6·8	17·3	16·9	9·3	10·8
			PBMC+iCEPS	19·5	24·1	13·4	25·6	26·4	15·7	12·3
			PBMC+B cell	35·1	30·5	25	35·5	33·9	17·6	17·3
		CD56-Ki67 (%)	PBMC	1·7	1·5	0·9	2·5	2·6	0·5	1·5
			PBMC+iCEPS	2·6	2·2	0·7	2	1·9	0·4	1·3
			PBMC+B cell	26	19·3	17·9	24·3	16·1	3·6	10·1
		IFN-γ (ng/mL)	PBMC	166	289	222	199	239	250	386
			PBMC+iCEPS	102	196	139	136	261	343	454
			PBMC+B cell	2256	1639	3877	1102	2380	1951	5957

Table S32. A repeated measures ANOVA test with Bonferroni correction of LSCD stage

	2 W	4 W	8 W	16 W	24 W	32 W	40 W	52 W
F (1, 3)	100·12	100·12	100·12	100·12	100·12	100·12	102·40	23·64
P value	0·002*	0·002*	0·002*	0·002*	0·002*	0·002*	0·002*	0·02

* Statistical significance with Bonferroni's correction.

Table S33. A repeated measures ANOVA test with Bonferroni correction of CDVA (LogMAR)

	2 W	4 W	8 W	16 W	24 W	32 W	40 W	52 W
F (1, 3)	79·37	44·81	30·41	41·57	50·79	91·41	28·83	15·44
P value	0·003*	0·007	0·01	0·008	0·006*	0·002*	0·01	0·03

* Statistical significance with Bonferroni's correction.

Table S34. A repeated measures ANOVA test with Bonferroni correction of corneal opacification (grade)

	2 W	4 W	8 W	16 W	24 W	32 W	40 W	52 W
F (1, 3)	27·99	15·21	27·99	27·99	16·36	16·36	16·36	17·64
P value	0·01	0·03	0·01	0·01	0·03	0·03	0·03	0·02

SUPPLEMENTARY REFERENCES

1. Yoshinaga Y, Soma T, Azuma S, et al. Long-term survival in non-human primates of stem cell-derived, MHC-unmatched corneal epithelial cell sheets. *Stem Cell Rep*. 2022; **17**(7): 1714-1729. doi:10.1016/j.stemcr.2022.05.018.
2. Yoshida S, Kato TM, Sato Y, et al. A clinical-grade HLA haplobank of human induced pluripotent stem cells matching approximately 40% of the Japanese population. *Med*. 2023; 4(1): 51-66. e10. doi:10.1016/j.medj.2022.10.003.
3. Hayashi R, Ishikawa Y, Katori R, et al. Coordinated generation of multiple ocular-like cell lineages and fabrication of functional corneal epithelial cell sheets from human iPS cells. *Nat Protoc*. 2017; **12**(4): 683-696. doi:10.1038/nprot.2017.007.
4. Hayashi R, Ishikawa Y, Katayama T, Quantock AJ, Nishida K. CD200 facilitates the isolation of corneal epithelial cells derived from human pluripotent stem cells. *Sci Rep*. 2018; **8**(1): 16550. doi:10.1038/s41598-018-34845-2.
5. Ministry of Health, Labour and Welfare of Japan notification., No.0613-3., 13 June 2016. https://www.mhlw.go.jp/content/10800000/000726910.pdf
6. Kuroda T, Yasuda S, Kusakawa S, et al. Highly sensitive in vitro methods for detection of residual undifferentiated cells in retinal pigment epithelial cells derived from human iPS cells. *PLoS One*. 2012; **7**(5): e37342. doi:10.1371/journal.pone.0037342.
7. Kono K, Takada N, Yasuda S, et al. Characterization of the cell growth analysis for detection of immortal cellular impurities in human mesenchymal stem cells. *Biologicals*. 2015; **43**(2): 146-149. doi:10.1016/j.biologicals.2014.11.007.
8. Sondka Z, Bamford S, Cole CG, Ward SA, Dunham I, Forbes SA. The COSMIC Cancer Gene Census: describing genetic dysfunction across all human cancers. *Nat Rev Cancer*. 2018; **18**(11): 696-705. doi:10.1038/s41568-018-0060-1.
9. http://www.pmda.go.jp/files/000152599.pdf
10. Ministry of Health, Labour and Welfare of Japan notification., No.0118-1., 18 January 2010., https://db.bioip-lab.org/uploads/attachment/file/177/4da4da7a-7230-4a56-8d87-cdb084c2e570.pdf
11. Deng SX, Borderie V, Chan CC, et al. Global consensus on definition, classification, diagnosis, and staging of limbal stem cell deficiency. *Cornea*. 2019; **38**(3): 364-375. doi:10.1097/ICO.0000000000001820.
12. Sotozono C, Ang LP, Koizumi N, et al. New grading system for the evaluation of chronic ocular manifestations in patients with Stevens-Johnson syndrome. *Ophthalmology*. 2007; **114**(7): 1294-1302. doi:10.1016/j.ophtha.2006.10.029.
13. Sugita S, Makabe K, Fujii S, et al. Detection of retinal pigment epithelium-specific antibody in iPSC-derived retinal pigment epithelium transplantation models. *Stem Cell Rep*. 2017; **9**(5): 1501-1515. doi:10.1016/j.stemcr.2017.10.003.
14. Fujiki F, Oka Y, Tsuboi A, et al. Identification and characterization of a WT1 (Wilms Tumour Gene) protein-derived HLA-DRB1*0405-restricted 16-mer helper peptide that promotes the induction and activation of WT1-specific cytotoxic T lymphocytes. *J Immunother*. 2007; **30**(3): 282-293. doi:10.1097/01.cji.0000211337.91513.94.

First-in-human clinical research of induced pluripotent stem (iPS) cell-derived corneal epithelial cell sheet transplantation for patients with limbal stem cell deficiency

Final Protocol

Principal investigator
Prof. Kohji Nishida, M.D., Ph.D.
Ophthalmology, Department of Ophthalmology, Graduate School of Medicine, Osaka University

Ver. 2.2
Prepared: May 18, 2020

Confidentiality statement

This protocol contains confidential information. Disclosure of the information is limited to persons involved in this research for conduct of the research only.

Disclosure of the protocol to a third party without obtaining written approval from the investigator is strictly prohibited. However, the protocol may be disclosed to subjects and witnesses when obtaining informed consent from subjects.

Revision history

Version no.	Date of revision	Rationale/details of the revision	Effective date
Ver. 1.0	January 8, 2019	Issued	—
Ver. 1.1	February 26, 2019	To address items raised at the 37th meeting of the Health Sciences Council (Subcommittee for Evaluation of Regenerative Medicine Products)	—
Ver. 1.2	March 15, 2019	To address instructions received at the 38th meeting of the Health Sciences Council (Subcommittee for Evaluation of Regenerative Medicine Products)	March 27, 2019
Ver. 1.3	May 15, 2019	Clarified that the items are data to be collected in Case Report Forms (6.3), added laboratory equipment (8.3), and corrected a mistranslation (9.2: 7))	May 15, 2019
Ver. 1.4	June 18, 2019	Changed exclusion criterion 4) (Synopsis, 3.2.2), changed blood collection volume and test parameters (8.7: 1), (4))	September 12, 2019
Ver. 2.0	October 30, 2019	・To address partial amendments to the Order for Enforcement of the Act on the Safety of Regenerative Medicine<br>・Extended the research period (Synopsis, 7), changed the duties of the principal investigator based on the conflict of interest management plan (4.4), 9.3, 9.4, 10.1.1, 10.4), changed the evaluation timing for secondary endpoints (degree of corneal opacification, degree of corneal neovascularization) (9.2: 6), 7)), changed who determines the regimen for the third and subsequent iCEPS transplantations and who is responsible for final evaluation (9.4, 9.5), registration in NRMD/CR (14.3)	December 19, 2019
Ver. 2.1	February 5, 2020	Added new registration number (Synopsis), changed medication dispensing rules (6.2), changed blood collection volume (8.7: 1), (3))	May 25, 2020
Ver. 2.2	May 18, 2020	・Additions due to the transfer of operations from the Center for iPS Cell Research and Application, Kyoto University (CiRA) to the CiRA Foundation (List of terms, 18.1.1)<br>・To address preliminary questions for the 49th meeting of the Health Sciences Council (Subcommittee for Evaluation of Regenerative Medicine Products) (6.2)	May 25, 2020

List of abbreviations and terms

List of abbreviations

Abbreviation	Description
ETDRS	Early Treatment Diabetic Retinopathy Study
LSCD	Limbal Stem Cell Deficiency

List of terms

Term	Description
HLA type	Human leukocyte antigen<br>* In this research, having at least the three loci HLA-A, -B, and -DR compatible with a CiRA-supplied iPS cell line established from HLA-homozygous donors with the most common Japanese haplotypes is defined as "HLA-matched," and any other case as "HLA-mismatched."
iCEPS	Abbreviation of "induced pluripotent stem cell-derived corneal epithelial cell sheet"
Center for iPS Cell Research and Application, Kyoto University (CiRA)	Operations were transferred to the CiRA Foundation on April 1, 2020.
Principal investigator	Refers to the "responsible party" as defined in the Act on the Safety of Regenerative Medicine (hereinafter referred to as "the Act").
Investigators	The principal investigator and subinvestigators. Refers to "physicians providing regenerative medicine" in the Act.
Head of the research site	Refers to "the administrator of the institution providing regenerative medicine" in the Act.
Subjects	Refers to "individuals receiving regenerative medicine" in the Act.

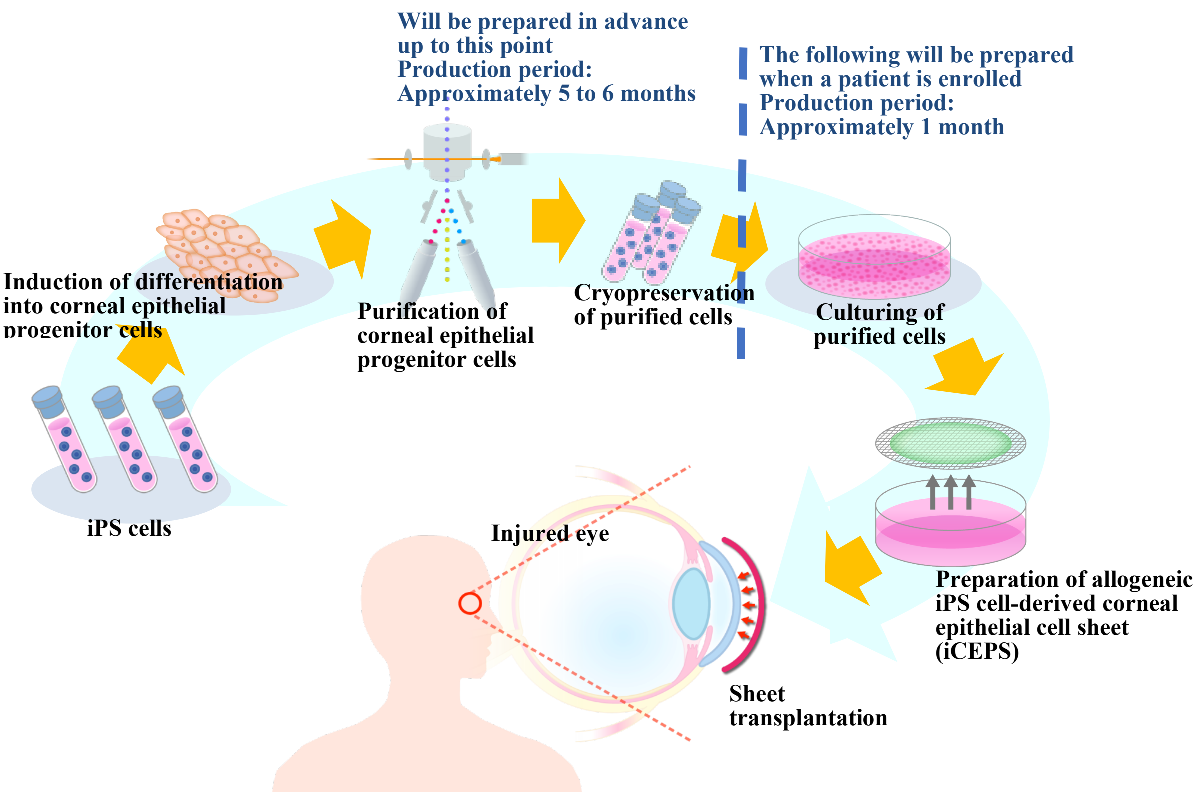

Transplantation of Allogeneic iPS Cell-derived Corneal Epithelial Cell Sheet

Synopsis

Objectives

To confirm the safety of transplantation of the specific processed cell product (induced pluripotent stem cell-derived corneal epithelial cell sheet [iCEPS]) in patients diagnosed with limbal stem cell deficiency (LSCD), evaluate the efficacy, and explore the feasibility of the protocol treatment.

Target disease

Limbal stem cell deficiency

Inclusion/exclusion criteria

Patients who meet all of the following inclusion criteria and none of the exclusion criteria are eligible for the research.

iCEPS will be transplanted only in one eye. If both eyes meet the transplantation criteria, the transplantation approach will be determined comprehensively by referring to the following items and consulting with the patient:

・Visual acuity
・Corneal stromal opacity
・Cataract
・Tear fluid volume, etc.

Inclusion criteria:

1) Patients diagnosed with LSCD classification stage IIB, IIC, or III

Figure 1

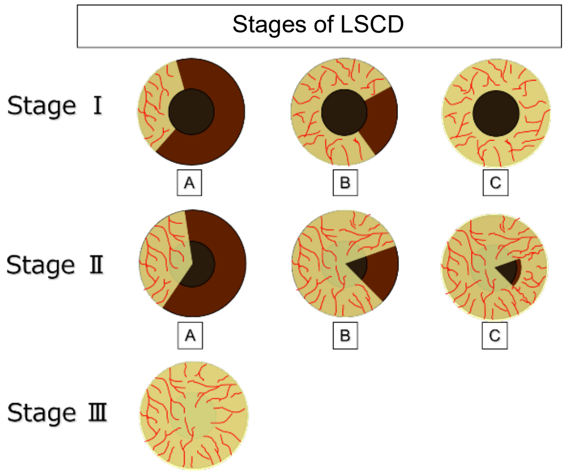

Stage I: No conjunctivalization is present in the center of the cornea (diameter 5 mm), and the percentage of conjunctivalization in the limbus is as follows:
A: <50%
B: ≥50% and <100%
C: 100%

Stage II: Conjunctivalization is present in the center of the cornea (diameter 5 mm), and the percentage of conjunctivalization in the limbus is as follows:
A: < 50%
B: ≥50% and <100%
C: 100%

Stage III: The whole corneal surface is covered by conjunctival tissue.

2) (1) iCEPS transplantation cases 1 and 2
Patients whose HLA type is mismatched with CiRA-supplied iPS cell lines established from donors homozygous for the most common HLA haplotypes in Japan
(2) iCEPS transplantation cases 3 and 4
Will be determined as follows based on the number of cases of uncontrolled rejection in the midterm evaluation of the first two iCEPS transplantation cases:

a. Zero cases of rejection: HLA-mismatched patients like cases 1 and 2
b. One case of rejection: HLA-mismatched patients like cases 1 and 2
c. Two cases of rejection: HLA-matched patients

3) Patients who are at least 20 years of age at the time of informed consent and who have given written consent to participate in this research.

Exclusion criteria:

1) Patients in whom antibacterial drugs, corticosteroids, immunosuppressive agents, or anesthetic drugs used in the clinical research are contraindicated (Patients who have “relative contraindications” will not be excluded.)
2) Patients who are allergic to antibiotics (e.g., penicillin, streptomycin)
3) History of allergy to animals (cows, pigs, rodents)
4) Patients with infection meeting any of the following criteria:
   (1) Active hepatitis B infection or a history of hepatitis B infection as determined by HBs antigen, HBs antibody, or HBc antibody testing
   (2) Active hepatitis C infection as determined by HCV antibody or HCV-RNA testing
   (3) Positive for HIV antibody
5) History of malignant tumor within 5 years prior to screening, or currently suspected with malignant tumor
6) Glaucoma with poorly controlled intraocular pressure
7) Diabetes mellitus with poor glycemic control
8) Pregnant women, lactating women, women who are possibly pregnant, or women who want to be pregnant during the clinical research period
9) Patients who have participated in another clinical study within 16 weeks prior to transplantation of iCEPS, who are currently participating in another clinical study, or who are scheduled to participate in another clinical study during participation in the present clinical research (Patients who are participating in observational research will not be excluded.)
10) Other patients who are not eligible for the clinical research in the opinion of the principal investigator or subinvestigators

Clinical research design
Single-center, open-label, non-controlled study

Method and timing of obtaining informed consent
Method
Written informed consent will be obtained directly from the patient.
Timing
Before screening tests and observations

Prohibited concomitant drugs and therapies
Prohibited concomitant drugs
None

Prohibited concomitant therapies<br>None
Name of specific processed cell product<br>Allogeneic iPS cell-derived corneal epithelial cell sheet<br>(iCEPS)
Details and timing of tests/observations<br>Tests/observations will be conducted according to the test/observation schedule (Table 1).
Endpoints<br>Primary endpoint<br>Safety will be evaluated by collecting adverse event data.<br>Secondary endpoints<br>The following efficacy items will be evaluated.<br>1) LSCD stage<br>2) Severity of corneal epithelial defect<br>3) Subjective symptoms<br>4) Corrected distance visual acuity<br>5) QOL<br>6) Degree of corneal opacification<br>7) Degree of corneal neovascularization<br>8) Degree of symblepharon
Planned sample size: 4 subjects
Research period: May 23, 2019 to March 31, 2024
Registration number for the Regenerative Medicine Provision Plan: jRCTa050190084 (former number: PA8180004)

Table 1 Test/Observation Schedule

Tests/observations \ Timing	Before enrollment	Day of transplantation			After transplantation (follow-up period)							
		Visit 1			Visit 2	Visit 3	Visit 4	Visit 5	Visit 6	Visit 7	Visit 8	Visit 9
	Screening	Day 0			Week 1 (Days 3–7)	Week 2 (Day 14)	Week 4 (Day 28)	Week 8 (Day 56)	Week 12 (Day 84)	Week 16 (Day 112)	Week 20 (Day 140)	Week 24 (Day 168)
Acceptable range	Within −56 days of enrollment					±3 days	±4 days	±7 days	±14 days	±14 days	±14 days	±14 days
		Before transplant-ation	During transplantation: After removal of conjunctival tissue	During transplantation: Immediately after transplantation								
Subject characteristics	○											
Slit-lamp microscopy and anterior segment photography	○	○*3	○*5	○*5	○*6	○	○	○		○		○
Anterior segment optical coherence tomography	○	○*3				○	○	○		○		○
Visual acuity test	○	○*3				○	○	○		○		○
Subjective symptoms	○	○*3				○	○	○		○		○
QOL	○	○*3										
Blood tests: Hematology	○	○*4				○	○	☆	○	☆	☆	○
Blood tests: Biochemistry	○	○*4				○	○	☆	○	☆	☆	○
Blood tests: Immunoserology	○	○*4				○	○		○			○
Blood tests: Tests for infectious diseases	○											
Blood tests: Tumor marker measurement*1	○						○		○			○
Blood tests: Trough level measurement						☆	☆	☆	☆	☆	☆	☆
Collection of blood sample for storage		○*4										
Urinalysis*2	○	○*3										

Tests/observations \ Timing		After transplantation (follow-up period)								AM period[*7]
		Visit 10	Visit 11	Visit 12	Visit 13	Visit 14	Visit 15	Visit 16	Day of discontinuation	
		Week 28 (Day 196)	Week 32 (Day 224)	Week 36 (Day 252)	Week 40 (Day 280)	Week 44 (Day 308)	Week 48 (Day 336)	Week 52 (Day 364)		Week 104 (Day 728)
Acceptable range		±14 days	±14 days	±14 days	±14 days	±14 days	±14 days	±14 days	Within +7 days	±28 days
Slit-lamp microscopy and anterior segment photography			○		○			○	○	○
Anterior segment optical coherence tomography			○		○			○	○	○
Visual acuity test			○		○			○	○	○
Subjective symptoms			○		○			○	○	○
QOL								○	○	
Blood tests	Hematology	☆	☆	○	☆	☆	☆	○	○	
Blood tests	Biochemistry	☆	☆	○	☆	☆	☆	○	○	
Blood tests	Immunoserology			○				○	○	
Blood tests	Tests for infectious diseases									
Blood tests	Tumor marker measurement[*1]			○				○	○	
Blood tests	Trough level measurement	☆	☆	☆	☆	☆	☆	☆	☆	

<u>During the clinical research period, monitoring of systemic adverse events will be conducted as needed through blood tests and patient interviews.</u>

<u>Pathological diagnosis (biopsy tissue diagnosis) will be performed if an obvious abnormality is observed in the corneal epithelium on slit-lamp microscopy. If a neoplastic lesion is observed, an X-ray CT scan will be performed.</u>

○: To be performed

☆: To be performed in subjects <u>receiving</u> immunosuppressive agents as specified in “6.2 Administration of Immunosuppressive Agents After iCEPS Transplantation”
*1: If values after iCEPS transplantation are higher than screening values and are abnormal, an oncologist will collaborate in the subject’s care.
*2: Urine pregnancy tests will be performed for women of childbearing potential.
*3: To be performed within −4 days of iCEPS transplantation
*4: To be performed on the day of iCEPS transplantation
*5: Anterior segment photographs should be obtained by extracting frames from surgical videos.
*6: Only slit-lamp microscopy will be performed.
*7: A one-year Additional safety Monitoring (AM) period will be performed after the end of the 52-week follow-up period.

Scheme

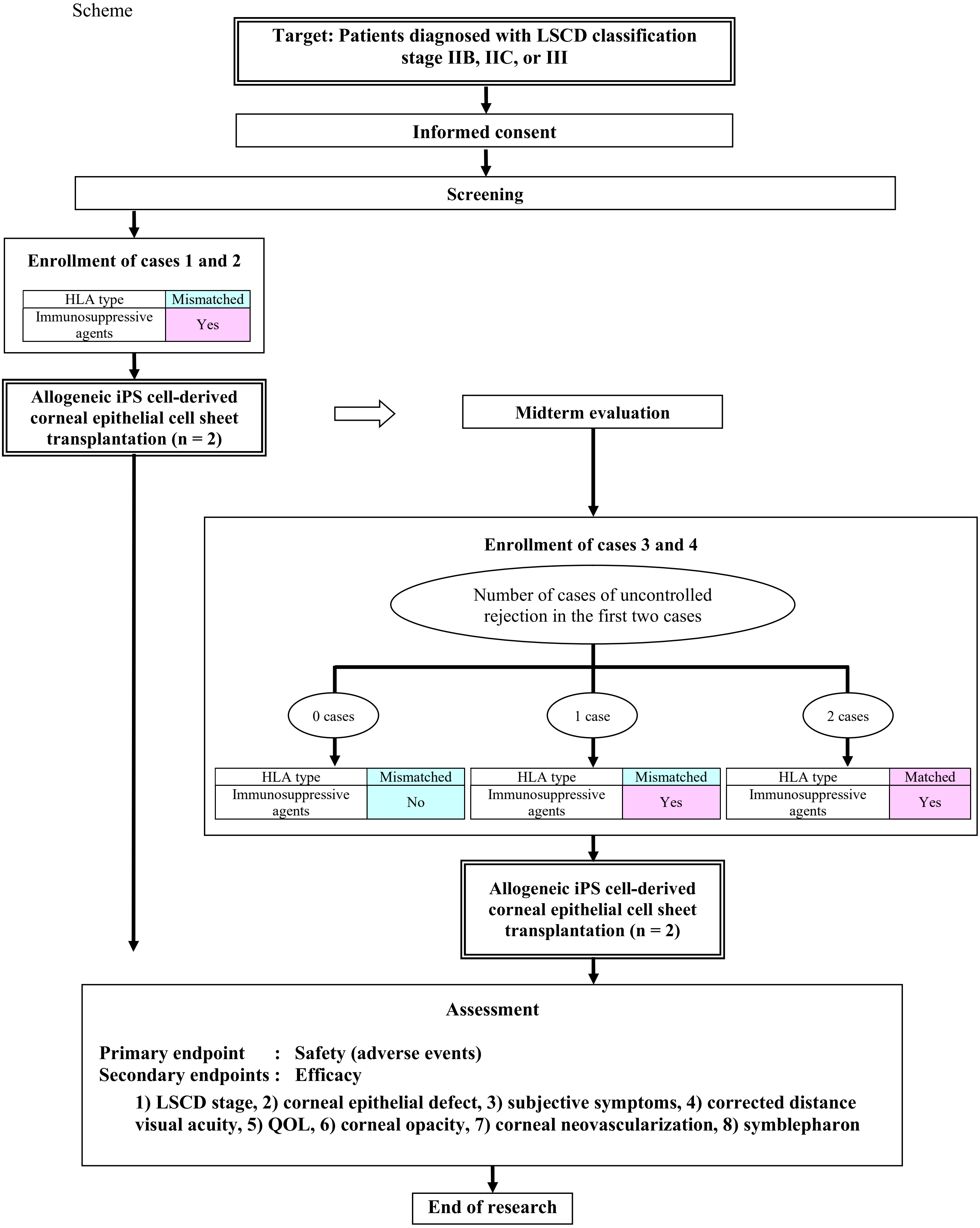
Target: Patients diagnosed with LSCD classification stage IIB, IIC, or III
Informed consent
Screening
Enrollment of cases 1 and 2
HLA type
Mismatched
Immunosuppressive agents
Yes
Allogeneic iPS cell-derived corneal epithelial cell sheet transplantation (n = 2)
Midterm evaluation
Enrollment of cases 3 and 4
Number of cases of uncontrolled rejection in the first two cases
0 cases
1 case
2 cases
HLA type
Mismatched
Immunosuppressive agents
No
HLA type
Mismatched
Immunosuppressive agents
Yes
HLA type
Matched
Immunosuppressive agents
Yes
Allogeneic iPS cell-derived corneal epithelial cell sheet transplantation (n = 2)
Assessment
Primary endpoint : Safety (adverse events)
Secondary endpoints : Efficacy
1) LSCD stage, 2) corneal epithelial defect, 3) subjective symptoms, 4) corrected distance visual acuity, 5) QOL, 6) corneal opacity, 7) corneal neovascularization, 8) symblepharon
End of research

Table of contents

1. Objectives 5
2. Background of Development and Rationale for Planning the Research 5
2.1 Description of Limbal Stem Cell Deficiency (LSCD) 5
2.2 Existing Therapies 5
2.2.1 Allogeneic limbal transplantation 5
2.2.2 Autologous limbal transplantation 6
2.3 Development of New Therapies 6
2.3.1 Autologous cultivated limbal epithelial cell sheet transplantation 6
2.3.2 Autologous cultivated oral mucosal epithelial sheet transplantation (COMET) 6
2.4 Rationale for Planning the Clinical Research 6
2.5 Research Design 8
3. Target Disease and Eligibility Criteria 9
3.1 Target Disease 9
3.2 Eligibility Criteria 9
3.2.1 Inclusion criteria 9
3.2.2 Exclusion criteria 11
4. Enrollment of Subjects 12
5. Cells and Specific Processed Cell Product To Be Used in Regenerative Medicine 13
5.1 Cells To Be Used in Regenerative Medicine (Cells Composing the Specific Processed Cell Product) 13
5.2 Specific Processed Cell Product 14
5.3 Overview of Preclinical Study Results 17
6. Treatment Plan and Criteria for Treatment Modification 18
6.1 Transplantation of Allogeneic iPS Cell-derived Corneal Epithelial Cell Sheet . 18
6.2 Administration of Medications After iCEPS Transplantation 19
6.3 Prohibited Concomitant Drugs and Therapies 19
6.3.1 Prohibited concomitant drugs 19
6.3.2 Prohibited concomitant therapies 19
6.4 Discontinuation of Subjects 20
6.4.1 Discontinuation criteria 20
6.4.2 Procedures and follow-up for subjects who discontinue the research .. 20
6.5 Pregnancy During the Clinical Research Period 20
7. Planned Sample Size and Research Period 21
8. Tests/Observations and Schedule 22

8.1 Subject characteristics ... 22
8.2 Slit-lamp Microscopy and Anterior Segment Photography ... 22
8.3 Anterior Segment Optical Coherence Tomography (AS-OCT) ... 23
8.4 Visual Acuity Test ... 23
8.5 Subjective Symptoms ... 23
8.6 QOL ... 24
8.7 Laboratory Tests ... 24
8.8 Histopathological Tests ... 26
8.9 X-ray CT Scan ... 26
8.10 Adverse Events ... 26
8.11 Acceptable Range of Test/Observation Schedule ... 26
8.12 Test/Observation Schedule ... 28

9. Definition of Efficacy and Safety Evaluations and Endpoints ... 30
9.1 Primary Endpoint ... 30
9.2 Secondary Endpoints ... 30
9.3 Decision Items for Individual Endpoints ... 33
9.4 Midterm Evaluation ... 33
9.5 Final Evaluation ... 34
9.6 Safety Monitoring Committee ... 34

10. Reporting Adverse Events ... 35
10.1 Definitions of Adverse Events, Serious Adverse Events, Malfunctions, Diseases, and Related Terms ... 35
10.1.1 Adverse events ... 35
10.1.2 Serious adverse events ... 35
10.1.3 Malfunctions ... 35
10.1.4 Diseases or the like ... 36
10.2 Assessment of Adverse Events ... 36
10.3 Criteria for Causality Assessment ... 36
10.4 Response and Follow-up Investigation After Occurrence of Adverse Events ... 37
10.5 Measures for Serious Adverse Events and Malfunctions ... 37
10.6 Measures for Diseases or the Like ... 37
10.7 Expected Adverse Events ... 39
10.7.1 Adverse events caused by transplantation surgery/procedures ... 39
10.7.2 Adverse events caused by the specific processed cell product (iCEPS) 39
10.7.3 Adverse events caused by general anesthesia ... 40

11. Discontinuation of the Entire Clinical Research ... 40
11.1 Discontinuation Criteria ... 40
11.2 Discontinuation Procedures ... 40

12. Case Report Forms ... 40

12.1 Preparation, Modification, and Correction of Case Report Forms 40
12.2 Review of Case Report Forms 41
12.3 Submission Deadlines for Case Report Forms 41
12.4 Specification of Case Report Form Entries That Are Regarded as Source Documents 41

13. Statistical Considerations 41
13.1 Definitions of Analysis Sets 41
13.2 Analyses and Methods 42
13.2.1 Disposition of subjects 42
13.2.2 Subject characteristics 42
13.2.3 Primary endpoint (safety) 42
13.2.4 Secondary endpoints (efficacy) 42
13.3 Significance Level To Be Used 43
13.4 Procedures for Handling Missing, Rejected, and Abnormal Data 43
13.5 Procedures for Changing the Analysis Plan 43

14. Ethical Considerations 43
14.1 Compliance with Regulatory Requirements 43
14.2 Informed Consent 43
14.2.1 Preparation of the patient information sheet and informed consent form 43
14.2.2 Revision of the patient information sheet and informed consent form. 45
14.2.3 Explaining about the research and obtaining informed consent from subjects 45
14.3 Protection of Personal Information 46
14.4 System for Handling Complaints and Inquiries from Subjects and Other Relevant Parties 46
14.5 Compensation for Health Injury 46
14.6 Handling of Cases When Significant Findings Regarding a Subject's Health or Inheritable Genetic Characteristics Are Obtained During the Course of the Research 46

15. Changes to the Regenerative Medicine Provision Plan 47
15.1 Non-minor Changes to the Regenerative Medicine Provision Plan 47
15.2 Minor Changes to the Regenerative Medicine Provision Plan 47
15.3 Changes to the Regenerative Medicine Provision Plan That Do Not Require Notification 47

16. Periodic Reports 47
16.1 Periodic Reports Regarding the Regenerative Medicine Provision Plan 47
16.1.1 Reporting to the Certified Committee for Regenerative Medicine 47
16.1.2 Reporting to the Minister of Health, Labour and Welfare 48
16.2 Reporting on Safety Information 48

16.3 Reporting of Noncompliance 49
16.4 End-of-study Report 49
16.5 Reporting of Discontinuation 49
16.5.1 Report from principal investigator to head of research site 49
16.5.2 Notification from the head of the research site to the Certified Committee for Regenerative Medicine and submission to the Minister of Health, Labour and Welfare 49
17. Clinical Study Report 50
18. Storage of Samples and Records 50
18.1 Recording and Storage of Records Related to the Regenerative Medicine 50
18.1.1 Records to be retained for 30 years 50
18.1.2 Records to be retained for 5 years 51
18.2 Storage of Samples 51
19. Direct Access to Source Documents 52
20. Monitoring and Audits 52
20.1 Monitoring 52
20.2 Audits 52
21. Sources of Funding for the Clinical Research and Conflicts of Interest 52
22. Burden of Research-related Expenses 53
23. Registration of the Clinical Research and Attribution and Publication of Results 53
23.1 Registration of the Clinical Research 53
23.2 Attribution and Publication of Results 53
24. Administrative Structure of the Research 53
25. References 54

1. Objectives

To confirm the safety of transplantation of the specific processed cell product (induced pluripotent stem cell-derived corneal epithelial cell sheet [iCEPS]) in patients diagnosed with limbal stem cell deficiency (LSCD), evaluate the efficacy, and explore the feasibility of the protocol treatment.

2. Background of Development and Rationale for Planning the Research

2.1 Description of Limbal Stem Cell Deficiency (LSCD)

The cornea consists of three main layers, epithelium, stroma, and endothelium, from the surface of the eyeball. It is understood that the integrity of the cornea is maintained by continuous and balanced turnover of the epithelium, the uppermost layer, with a cycle of proliferation (X), migration (Y), and sloughing (Z) expressed as X + Y = Z[1)]. The corneal epithelial stem cell is thought to be located in the limbus, which is the narrow transitional zone of the ocular surface located between the cornea and bulbar conjunctiva[2)], as a source of corneal epithelial cells. LSCD is a condition in which corneal epithelial stem cells disappear and is characterized by invasion of vascularized conjunctival epithelium to the corneal surface, inducing opacity and reduction in visual acuity. Causes of LSCD include aniridia and sclerocornea as congenital anomalies, alkali corrosion or thermal burn as exogenous causes, and Stevens–Johnson syndrome or ocular pemphigoid as endogenous causes, but in some cases, the cause is unknown[3)].

2.2 Existing Therapies

Existing therapies for LSCD are allogeneic limbal transplantation and autologous limbal transplantation[4-13)]. Another option is medication including antibiotics that alleviate subjective symptoms and prevent LSCD from worsening.

2.2.1 Allogeneic limbal transplantation

In this therapy, the conjunctival pannus is completely removed from the injured eye, and limbal tissue collected from a cornea donor is transplanted. The limbus covers the cornea of the injured eye and protects the cornea from invasion of conjunctival tissue or invasive cells. However, this therapy has two major problems. The first problem is rejection. It is known that limbal transplantation shows a high rejection rate (17-52%) when used in patients with LSCD, a disease of the corneal epithelium[5, 7, 8)]. Even if an immunosuppressive agent is administered, a long-term therapeutic effect cannot be expected, and prognosis is poor. Another problem is shortage of donors. The Japan Eye Bank Association reported that 1,624 patients are waiting for transplantation as of the end of 2017, but only 869 persons donate their eyes a year, indicating a serious shortage of donors.

2.2.2 Autologous limbal transplantation

In this therapy, two biopsy samples of limbal tissue are collected from the uninjured eye of the patient himself/herself and transplanted to the injured eye. This treatment can circumvent the problem of rejection, but a graft in a size of approximately 1/6 to 2/3 of the limbus is necessary to be taken from the healthy eye, which brings about serious damage to the uninjured eye. Adverse events including local corneal opacity, pseudopterygium, filamentary keratitis, and epithelium hypoplasia have been reported. Furthermore, the possibility of occurrence of LSCD in the uninjured eye cannot be ruled out. As a result, autologous limbal transplantation is rarely used these days. In addition, autologous limbal transplantation is not possible for bilateral LSCD.

2.3 Development of New Therapies

Clinical research and clinical trials on transplantation of autologous cultivated limbal epithelial cell sheets derived from autologous limbal tissue and cultivated autologous oral mucosal epithelial cell sheets derived from autologous oral mucosal cells (COMET) are being conducted to address problems with existing therapies, and new therapies are in development.

2.3.1 Autologous cultivated limbal epithelial cell sheet transplantation

Cells are isolated from normal limbal tissue harvested from a patient’s uninjured eye and cultivated into a limbal epithelial cell sheet, which is then transplanted into the injured eye. The cell sheet is produced using a roughly 2 × 3-mm area of limbal tissue, which minimizes damage to the uninjured eye, and transplantation of this cell sheet can be expected to produce a sustained therapeutic effect because post-transplantation rejection does not occur. Clinical research on autologous cultivated limbal epithelium conducted outside Japan showed no adverse events in the unaffected eye and confirmed long-term efficacy[14)]. In February 2015, the European Medicines Agency (EMA) granted conditional manufacturing and marketing approval for an autologous cultured corneal epithelium product called Holoclar. A sponsor-initiated clinical trial of a similar product is underway in Japan (as of March 2018).

2.3.2 Autologous cultivated oral mucosal epithelial sheet transplantation (COMET)

Epithelial cells isolated from normal oral mucosal tissue harvested from the patient are cultivated into an oral mucosal epithelial sheet, which is then transplanted into the injured eye. This method avoids rejection by using autologous tissue and can also be used for bilateral LSCD. Exploratory clinical research conducted in Japan to date has shown that this treatment can provide better outcomes than conventional allogeneic limbal transplantation[15-17)]. In addition, an investigator-initiated clinical trial was conducted with the aim of obtaining insurance coverage for the treatment (as of March 2018).

2.4 Rationale for Planning the Clinical Research

In LSCD, the loss of corneal epithelial stem cells leads to re-epithelialization by the vascularized conjunctival epithelium (conjunctivalization), which causes subjective symptoms such as eye pain, foreign body sensation, lacrimation, photophobia, dryness or discomfort, and visual impairment caused by corneal opacity[18, 19)].

Treatment of LSCD requires removal of the conjunctival tissue present on the cornea and corneal epithelial reconstruction by transplantation of corneal epithelial stem cells. Currently available treatments include allogeneic limbal transplantation and autologous limbal transplantation. However, a long-term therapeutic effect cannot be expected for allogeneic limbal transplantation because of rejection. Damage to the uninjured eye from which tissue is collected is an inherent problem of autologous limbal transplantation; in addition, it cannot be used for bilateral LSCD.

Autologous cultivated limbal epithelial cell sheet transplantation is also being developed as a novel treatment, and although this approach circumvents the problem of post-transplantation immune rejection, it cannot be used for bilateral LSCD. In addition, in some cases, autologous cultivated oral mucosal epithelial sheet transplantation (COMET) has had limited long-term effects because the transplanted oral mucosa has not completely converted to corneal epithelium. Because both treatment methods use autologous cells, production of cultured cell sheets itself may be difficult depending on the condition of the harvested tissues and cells. Therefore, no safe and effective treatment currently exists for LSCD.

To address this need, we have been developing a human iPS cell-based regenerative therapy for corneal epithelium. To date, our team has been the first in the world to establish an innovative method for induction and isolation of corneal epithelial progenitor cells from human iPS cells and technology for production of human iPS cell-derived corneal epithelial cell sheets[20)]. The technology we developed is a two-dimensional culture system that promotes autonomous differentiation of human iPS cells and reproduces the development of the entire eye in a petri dish. In this culture system, a two-dimensional tissue body (called a "self-formed ectodermal autonomous multi-zone," SEAM) with four concentric zonal structures is induced from human iPS cells. The major cell groups that comprise the developing eye (including the corneal epithelium, retina, and lens epithelium) appear at specific sites in the SEAM. We isolated corneal epithelial progenitor cells from the third zonal structure of this SEAM and successfully produced functioning corneal epithelial tissue. In addition, the therapeutic efficacy and safety of human iPS cell-derived corneal epithelial tissue has been demonstrated through transplantation into animal models, and various studies have verified its safety, for example, that it does not cause tumorigenesis (see "5.3 Overview of Preclinical Study Results"). The above facts indicate that with sufficient control of predictable risks, iPS cell-derived corneal epithelial cell sheets can be expected to provide benefits (corneal epithelial reconstruction, improved visual acuity, and relief from subjective symptoms) that outweigh the risks.

The most preferable approach from the viewpoint of transplantation immunity would be autologous transplantation of target cells derived from autologous iPS cells, but establishment of iPS cells and differentiation of those cells into target cells are uncertain and time-consuming processes with extremely high costs. These problems could be addressed through allogeneic transplantation of iPS cells generated from an HLA-matched donor. CiRA is aiming to establish a therapeutic iPS cell stock usable in future cell transplantation treatments by using multiple iPS cell lines established from donors who are HLA-homozygous at three loci: HLA-A, -B, and -DR. Whereas autologous transplantation requires iPS cells to be generated for each patient, this new method allows for use of

homogenized iPS cells and dramatically reduces the time required to generate therapeutic cells.

However, the only iPS cell lines currently available for clinical use are established from HLA-homozygous donors covering the most common haplotypes in the Japanese population, which limits the number of patients who can receive HLA-matched transplantations. As the specific processed cell product to be transplanted in this clinical research consists solely of epithelial cells, rejection should be less likely in allogeneic transplantation than in donor limbal transplantation, which involves transplantation of the corneal epithelium, corneal parenchyma, and limbus. In addition, use of immunosuppressive agents may further reduce the incidence of rejection. Based on the above rationale, this clinical research will evaluate the safety and efficacy of iCEPS transplantation in combination with immunosuppressive agents in patients whose HLA type is mismatched with iPS cells derived from homozygous donors (cases 1 and 2). For cases 3 and 4, the patient's HLA type and use of immunosuppressive agents will be determined based on the results of midterm evaluation of cases 1 and 2 in order to ensure the subjects' safety. Once the safety of iCEPS transplantation is confirmed through this clinical research and the effects of HLA compatibility and need for use of immunosuppressive agents are clarified, we plan to optimize the treatment design based on those findings and evaluate the therapeutic effects of iCEPS transplantation in the next phases of clinical trials.

2.5 Research Design

This clinical research is designed as a single-center, open-label, non-controlled study for the following reasons:

1) No definitive treatment is currently available for LSCD.
2) Blinding is not feasible because the treatment involves surgery.
3) The rarity of LSCD, the target disease of this research, would make random allocation of patients unfeasible.

3. Target Disease and Eligibility Criteria

3.1 Target Disease

Limbal stem cell deficiency

3.2 Eligibility Criteria

Patients who meet all of the inclusion criteria and none of the exclusion criteria are eligible for the research.

iCEPS will be transplanted only in one eye. If both eyes meet the transplantation criteria, the transplantation approach will be determined comprehensively by referring to the following items and consulting with the patient:

- Visual acuity
- Corneal stromal opacity
- Cataract
- Tear fluid volume, etc.

3.2.1 Inclusion criteria

1) Patients diagnosed with LSCD classification stage IIB, IIC, or III

Figure 1

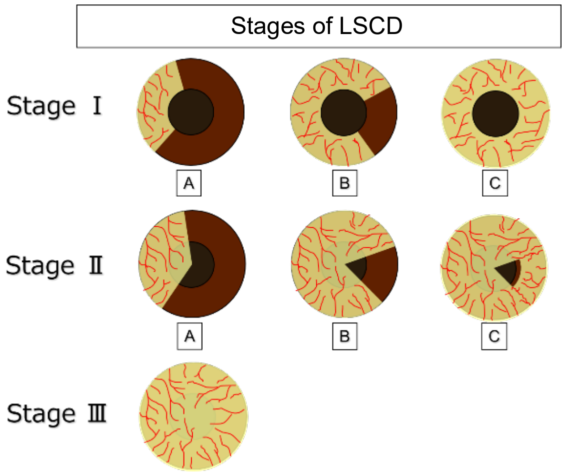

Stage I: No conjunctivalization is present in the center of the cornea (diameter 5 mm), and the percentage of conjunctivalization in the limbus is as follows:

A: < 50%
B: ≥50% and <100%
C: 100%

Stage II: Conjunctivalization is present in the center of the cornea (diameter 5 mm), and the percentage of conjunctivalization in the limbus is as follows:

A: < 50%
B: ≥50% and <100%
C: 100%

Stage III: The whole corneal surface is covered by conjunctival tissue.

2) (1) iCEPS transplantation cases 1 and 2

Patients whose HLA type is mismatched with CiRA-supplied iPS cell lines established from donors homozygous for the most common HLA haplotypes in Japan

(2) iCEPS transplantation cases 3 and 4

Will be determined as follows based on the number of cases of uncontrolled rejection in the midterm evaluation of the first two iCEPS transplantation cases:

a. Zero cases of rejection: HLA-mismatched patients like cases 1 and 2

b. One case of rejection: HLA-mismatched patients like cases 1 and 2

c. Two cases of rejection: HLA-matched patients

3) Patients who are at least 20 years of age at the time of informed consent and who have given written consent to participate in this research.

[Rationale for setting]

1) Subjects were limited to patients with LSCD who had no treatment option besides limbal transplantation. Stage I LSCD does not require limbal transplantation because the center of the cornea is covered by a transparent corneal epithelium and vision is unaffected, and stage IIA can be treated without limbal transplantation because more than 50% of the normal limbus remains[11, 21)]. Therefore, patients diagnosed with stage IIB or higher were selected as the subjects.

2) (1) iCEPS transplantation cases 1 and 2
It was decided to include HLA-mismatched patients as the first and second cases to examine whether this therapy could be used for a broader group of patients. A preclinical study in cynomolgus monkeys conducted prior to this clinical research showed a low rate of rejection with HLA-mismatched corneal epithelial cell sheet transplantation, even without the use of immunosuppressive agents. Therefore, coadministration of immunosuppressive agents should minimize the incidence of rejection.

(2) iCEPS transplantation cases 3 and 4
The protocol for cases 3 and 4, which is based on the number of cases of uncontrolled rejection in the midterm evaluation of the first two iCEPS transplantation cases, was set as follows:

a. Zero cases of rejection: The protocol was set to not include administration of immunosuppressive agents to HLA-mismatched patients, with the aim of exploring a protocol that could further reduce patient burden.

b. One case of rejection: The protocol for cases 3 and 4 was set to the same protocol for cases 1 and 2 in order to continue investigating the possibility of protocol treatment with coadministration of immunosuppressive agents in HLA-mismatched patients.

c. Two cases of rejection: The protocol was set so that cases 3 and 4 would be HLA-matched patients because findings indicate it would be difficult to control rejection by immunosuppressive agents in HLA-mismatched iCEPS transplantation.

3) This criterion is defined for the following reasons:
- The patient can understand the details of the clinical research and decide whether to participate in the research voluntarily.
- The patient can understand and properly answer the QOL questionnaires.

3.2.2 Exclusion criteria

1) Patients in whom antibacterial drugs, corticosteroids, immunosuppressive agents, or anesthetic drugs used in the clinical research are contraindicated (Patients who have "relative contraindications" will not be excluded.)
2) Patients who are allergic to antibiotics (e.g., penicillin, streptomycin)
3) History of allergy to animals (cows, pigs, rodents)
4) Patients with infection meeting any of the following criteria:
   (1) Active hepatitis B infection or a history of hepatitis B infection as determined by HBs antigen, HBs antibody, or HBc antibody testing
   (2) Active hepatitis C infection as determined by HCV antibody or HCV-RNA testing
   (3) Positive for HIV antibody
5) History of malignant tumor within 5 years prior to screening, or currently suspected with malignant tumor
6) Glaucoma with poorly controlled intraocular pressure
7) Diabetes mellitus with poor glycemic control
8) Pregnant women, lactating women, women who are possibly pregnant, or women who want to be pregnant during the clinical research period
9) Patients who have participated in another clinical study within 16 weeks prior to transplantation of iCEPS, who are currently participating in another clinical study, or who are scheduled to participate in another clinical study during participation in the present clinical research (Patients who are participating in observational research will not be excluded.)
10) Other patients who are not eligible for the clinical research in the opinion of the principal investigator or subinvestigators

[Rationale for setting]

1) To ensure safety of the patients, because antibacterial drugs, corticosteroids, immunosuppressive agents, and anesthetic drugs are necessary for transplantation of iCEPS.
2) To ensure safety of the patients, because the possibility of hypersensitivity or allergic reaction induced by residual antibiotics in iCEPS cannot be completely ruled out
3) To ensure safety of the patients, because the possibility of hypersensitivity or allergic reaction induced by residual materials of animal origin in iCEPS cannot be completely ruled out.
4) To ensure safety of the patients, because use of immunosuppressive agents may exacerbate infections.
5) To ensure safety of the patients. In general, malignant tumors can be considered to have been completely cured when there is no recurrence 5 years after treatment.
6) To ensure safety of the patients, because glaucoma may worsen because of increase in intraocular pressure by corticosteroid[22) administered after transplantation of iCEPS.

7) Diabetes mellitus may be complicated with corneal epithelium disorder or retinal disorder[23)], which may interfere with proper evaluation of efficacy and safety of the protocol treatment.
8) To ensure safety of the patients.
9) Repeated participation in clinical studies is ethically not acceptable, and such participation may interfere with proper evaluation of efficacy and safety of the protocol treatment.
10) General consideration for a clinical research.

4. Enrollment of Subjects

Enrollment of subjects is completed as follows.

1) Informed consent
The principal investigator or a subinvestigator obtains written informed consent from candidate patients after explaining about the clinical research in detail by using the patient information sheet.

2) Creation of a subject roster
The principal investigator or a subinvestigator assigns a subject identification code to each patient who gives written informed consent to participate in the research and will record that code in the subject roster. The principal investigator stores the subject roster at a secure location in the research site. The subject identification code consists of a 7-digit alphanumeric code, made from the five alphabet characters "iCEPS," which is an abbreviation for the specific processed cell product to be used in this research, plus a two-digit number that identifies the subject. The two-digit numbers are assigned in the order of informed consent, starting with the number 01 (e.g., iCEPS-01).

3) Screening tests
The principal investigator or a subinvestigator performs screening tests as stipulated in "8. Tests/Observations and Schedule" for subjects who have given written consent to participate in the research and are listed in the subject roster.

4) Determination of eligibility
The subinvestigator who will be the subject's physician determines the subject's eligibility based on the results of the screening tests. The decision is made by the subinvestigator and a physician-evaluator not directly involved in the conduct of this research.

5) Entry into the case registration system
The principal investigator or a subinvestigator enters information regarding the inclusion/exclusion criteria for eligible subjects into the enrollment page of the case registration system.

6) Enrollment and initiation of protocol treatment
The principal investigator or a subinvestigator reviews the "Eligibility Check Page" in the case registration system. If eligibility is confirmed by the case registration system,

a registration number is displayed, and enrollment in the research is complete. The registration number consists of a one-digit number (1) identifying the site and a two-digit serial number for assignment within the site (e.g., 1-01). A subject is considered enrolled in the research when a registration number is assigned. The principal investigator or a subinvestigator copies the registration number to the subject roster. Protocol treatment begins after subject enrollment is complete.

7) Once a subject is enrolled, his/her enrollment may not be canceled unless the subject withdraws consent.

8) The principal investigator and subinvestigators are required to promptly report erroneous/duplicate enrollments to the Data Center.

<u>Data Center</u>

Data Center, Department of Medical Innovation, Osaka University Hospital
2-2 Yamadaoka, Suita, Osaka 565-0871, Japan
Phone: 06-6210-8318
Fax: 06-6210-8320

5. Cells and Specific Processed Cell Product To Be Used in Regenerative Medicine

5.1 Cells To Be Used in Regenerative Medicine (Cells Composing the Specific Processed Cell Product)

1) Name of cells
   HLA-homozygous donor-derived cord blood
2) Medical institutions accepting cell donations
   Name: Kyoto University
   Address: 53 Shogoin-Kawaharacho, Sakyo-ku, Kyoto
3) Cell donor selection and eligibility confirmation
   As described in the “Request for participation in research to establish a therapeutic iPS cell stock from cord blood of donors homozygous for common HLA types” information sheet for cell donors at CiRA
4) Cell procurement
   Human cord blood previously collected at partner sites of Tokai University Cord Blood Bank has been transferred to CiRA. CiRA staff members conduct donor eligibility tests and use cells from eligible donors as the raw materials to make processed cell products. In addition, the following must be observed to ensure proper conduct of the research.
   (1) Consideration of the window period in cell donor eligibility confirmation
   Cell donor eligibility is confirmed by proving the absence of infectious disease through the following tests at the time the cells are accepted into Kyoto University, which will be after the window period.

・Nucleic acid amplification testing (NAT) using donor blood
HBV, HCV, HIV1, HIV2, HTLV-1 and -2, PB19

(2) Measures necessary to prevent microbial contamination during cell donation
Cord blood donation sites have instituted the "Standards for Cord Blood Collection" and "Standard Operating Procedures for Cord Blood" in accordance with the "Ministerial Ordinance on Standards for Quality Assurance of Cord Blood for Transplantation" (MHLW Ordinance No. 139; December 27, 2013). Based on these procedures, staff members take necessary measures, such as collecting samples in a sanitary environment, to prevent contamination by pathogenic microbes and other disease-causing agents during the collection process. They carry out actual sample collection tasks as described in the procedure manual and keep records of those tasks.

(3) Appropriate tests for microbial contamination and presence of microbes in the donated cells
The following blood tests are performed on collected cord blood to check the blood for microbial contamination and rule out the presence of microbes.

・HLA genotyping
Method: r-SSOP: Luminex method (WAK Flow, LABType)

・Tests for infectious diseases
Serological tests
Syphilis TPHA, HBsAg, HBcAb, HCVAb, HIVAb, HTLVAb
Nucleic acid amplification test (NAT)
CMV

5) Measures to be taken when cell safety concerns arise
When concerns regarding the safety of applicable cells arise, the production control manager will promptly report those concerns to the principal investigator. Upon receipt of the report, the principal investigator will instruct the applicable investigators to take necessary measures.

5.2 Specific Processed Cell Product

1) Name of specific processed cell product
Allogeneic iPS cell-derived corneal epithelial cell sheet
(iCEPS)

2) Profile
The specific processed cell product (iCEPS) has the following characteristics and is aseptically filled and sealed in dedicated containers before delivery to the research site. It is produced at the Medical Center for Translational Research Cell Processing Center (MTR-CPC) at Osaka University Hospital.

(1) It is an "allogeneic iPS cell-derived corneal epithelial cell sheet" that is produced at CiRA using iPS cells established from cord blood supplied by HLA-homozygous donors.

(2) Temperature-responsive culture dishes are used as culture vessels.

(3) Sheet shape with an effective diameter of 22 mm

(4) The serial number of the specific processed cell product is printed on a label attached to the surface of the container indicating that it has been approved for shipment.

3) Production and quality control of the specific processed cell product

Production and quality control of the specific processed cell product are conducted at three facilities in the following three phases.

(1) Production process from donor cells to creation of iPS cell stock

・ Name of cell culturing and processing facility: Facility for iPS Cell Therapy (FiT) at CiRA

・Address of cell culturing and processing facility: 53 Shogoin-Kawaharacho, Sakyo-ku, Kyoto

・Overview of methods for production and quality control of the specific processed cell product at the facility

a. Establishment of iPS cells
iPS cells are established using HLA-homozygous donor-derived cord blood as the source cells.

b. Generation of iPS cell stocks
Established iPS cells are expanded and cultured to produce iPS cell stocks.
(Delivered cells and created cell stocks are tested to control quality)

・Assigned tasks: Establishment of iPS cells and creation of iPS cell stock

(2) Production process from iPS cell stocks to creation of iPS cell intermediates

・ Name of cell culturing and processing facility:
Center for Gene and Cell Processing, Takara Bio Inc.

・ Address of cell culturing and processing facility: 7-4-38 Nojihigashi, Kusatsu, Shiga

・ Overview of methods for production and quality control of the specific processed cell product at the facility

c. Creation of iPS cell intermediates
Delivered iPS cell stocks are thawed and expanded to create iPS cell intermediates.
(Delivered cell stocks and created intermediates are tested to control quality)

・Assigned tasks: Creation of iPS cell intermediates

(3) Production process from iPS cell intermediates to release of the specific processed cell product

- Name of cell culturing and processing facility: Medical Center for Translational Research Cell Processing Center (MTR-CPC) at Osaka University Hospital
- Address of cell culturing and processing facility: 2-15 Yamadaoka, Suita, Osaka
- Overview of methods for production and quality control of the specific processed cell product at the facility

  The following steps through "f." below will be performed in advance. Steps from "g." onward will be performed in accordance with the patient enrollment schedule, and the released specific processed cell product will be supplied for administration.

  d. Passaging of iPS cells and seeding for differentiation
  Delivered iPS cell intermediates are thawed and passaged, and cells designated for differentiation are seeded.

  e. Induction of differentiation
  Seeded iPS cells are cultured for about 10 to 12 weeks to induce differentiation.

  f. Cell purification and cryopreservation
  Corneal epithelial progenitor cells are separated from differentiated induced cells and cryopreserved.

  g. Culture of purified cells
  Cryopreserved corneal epithelial progenitor cells are thawed and cultured to produce cell sheets.

  h. Release
  Specification tests are conducted, and the specific processed cell product is released after it is confirmed that the test results conform to release specifications.
- Assigned tasks: None

4) Procedures for control of the specific processed cell product
On the day of the transplantation procedure, staff at the Medical Center for Translational Research Cell Processing Center (MTR-CPC) at Osaka University Hospital will package the specific processed cell product in containers and carry those containers out of the MTR-CPC. A product release decision will be made for each unit of specific processed cell product carried out of the MTR-CPC, and when a unit is approved for release, a label printed with the serial number will be affixed to the surface of the container. After the release decision, the product will be promptly transported to the operating room and kept in its container until immediately before use. The product should be stored at room temperature. The expiration date is 24 hours from the release decision.

5.3 Overview of Preclinical Study Results

1) Overview of nonclinical efficacy and safety studies

Study items	Results
Transplantation of human iPS cell-derived corneal epithelial cell sheets into rabbit disease models	The corneal epithelium was reconstructed, and corneal transparency and barrier function were observed (follow-up period: 2 weeks, n = 7).<br>No abnormalities such as tumor formation or cell migration were observed (follow-up period: 3 weeks, n = 7).<br>Sample tested: Processed product for use in nonclinical studies (iPS cell line: 1383D2)

2) Overview of nonclinical tumorigenicity studies

Study items	Results
Study on subcutaneous transplantation (cell sheet administration) in immunocompromised mice (1)	Autopsy was performed after a 16-week follow-up period. Tumor formation was not observed.<br>(n = 1)
Study on subcutaneous transplantation (cell sheet administration) in immunocompromised mice (2)	Autopsy and histopathological observations were performed after a 16-week follow-up period. Tumor formation was not observed.<br>(n = 12)
Karyotyping	No cytogenetic abnormalities were observed.<br>(n = 1)
Cell proliferation characteristic analysis (serial passaging)	Cell transformation and immortalization were not observed.<br>(n = 1)
Study to rule out the presence of undifferentiated iPS cells (1) (measurement of *LIN28A* expression)	Below detection limit to 2.5 (copies/RNA 50 ng)<br>(n = 6)<br>All conformed to the reference value of ≤ 20
Study to rule out the presence of undifferentiated iPS cells (2) (reculture in medium used for iPS cells)	No undifferentiated iPS cell colonies were detected.<br>(n = 1)
Genome analysis	No genomic abnormalities were observed.<br>(n = 1)

3) (Reference data) Overview of nonclinical tumorigenicity studies using the processed cell product for use in nonclinical studies

Study items	Results
Study of subcutaneous transplantation in immunocompromised mice (cell suspension administration)	Tumor formation was not observed (follow-up period: 16 weeks; nude mice: n = 10, NOD-SCID mice: n = 21).
Study of subcutaneous transplantation in immunocompromised mice (cell sheet administration)	Tumor formation was not observed (follow-up period: 16 weeks; NOD-SCID mice: n = 6, NOG mice: n = 2).

Karyotyping	No cytogenetic abnormalities were observed (n = 5).
Cell proliferation characteristic analysis (serial passaging)	Cell transformation and immortalization were not observed (n = 3).
Study to rule out the presence of undifferentiated iPS cells (1) (measurement of *LIN28A* expression)	*LIN28A* expression was very low (2.4 copies to ≤ detection limit, n = 24).
Study to rule out the presence of undifferentiated iPS cells (2) (reculture in medium used for iPS cells)	No residual undifferentiated iPS cell colonies were detected (n = 22).
Genome analysis	Although SNV/InDel analysis showed mutation sites corresponding to the PMDA/Census list and CNV analysis showed loss, no homozygous mutations directly affecting gene expression were detected in either analysis (samples derived from cell line 1383D2, n = 2).

6. Treatment Plan and Criteria for Treatment Modification

6.1 Transplantation of Allogeneic iPS Cell-derived Corneal Epithelial Cell Sheet

The protocol treatment for this research is defined as allogeneic iPS cell-derived corneal epithelial cell sheet (iCEPS) transplantation.

It will be performed in accordance with the following procedures.

1) Upon receipt of the iCEPS from the person in charge of production, the principal investigator or subinvestigator confirms whether the iCEPS was produced in accordance with the Specific Processed Cell Product Summary using the release decision chart and decides whether or not to administer the product. They also check the condition of the product preservation solution (to ensure no turbidity or foreign matter). The product must not be used if any turbidity or foreign matter is observed.*
2) The product is transplanted under local anesthesia or systemic anesthesia. Transplantation of iCEPS is carried out by hospitalization, and the scheduled hospitalization period is about 3 to 4 weeks.
3) The conjunctiva scar is removed as much as possible from the cornea of the injured eye.
4) Symblepharon, if any, is treated simultaneously by conjunctival sac plasty or amniotic membrane transplantation to the sclera as necessary.
5) The temperature-responsive culture dish containing the cell sheet is removed to a clean area using sterile forceps or another suitable instrument.
6) The cell sheet support is grasped and lifted with sterile forceps or another suitable instrument, and the cell sheet is carefully peeled off. The cell sheet on the temperature-responsive culture dish is transplanted without reversing the top and bottom.

7) iCEPS is placed on the transplantation site. The cell sheet is cut along the perimeter with a scalpel, scissors, or trepan to remove, and the cell sheet is left as it is for approximately 15 minutes. Attachment of iCEPS to the transplantation site is confirmed.
8) After transplantation, a soft therapeutic contact lens is placed, tarsorrhaphy is performed as necessary, the eyelid is closed and fixed, and the subject is placed at rest.
   * The results of genetic analysis performed for reference purposes will be available approximately 4 months after transplantation. If a genetic abnormality is found, the subject will be informed, more careful post-treatment follow-up will be conducted, and appropriate measures will be taken.

6.2 Administration of Medications After iCEPS Transplantation

1) All subjects will receive systemic and local corticosteroids and local antibiotics. The medications, dose, and duration of administration may be modified as appropriate based on the subject's condition.

2) An immunosuppressive agent (cyclosporine) will be administered after iCEPS transplantation to the following subjects:
   (1) iCEPS transplantation cases 1 and 2
   (2) iCEPS transplantation cases 3 and 4, if uncontrolled rejection occurred in 1 or both of the first 2 cases, in accordance with "9.4 Midterm Evaluation"

   In general, cyclosporine should be administered from the day of iCEPS transplantation (after transplantation) until Week 52 after transplantation. Trough levels should be checked, and the dose should be adjusted accordingly.

3) Immunosuppressive agents other than cyclosporine (e.g., mycophenolate mofetil) may generally be used during the perioperative period to treat the primary disease.

Compliance with medication and eye drop use will be confirmed by collecting empty medication sheets and bottles from subjects and interviewing the subjects.

6.3 Prohibited Concomitant Drugs and Therapies

6.3.1 Prohibited concomitant drugs

None

6.3.2 Prohibited concomitant therapies

None

Drugs and therapies used concomitantly during the clinical research period will be documented in the Case Report Form (CRF).

6.4 Discontinuation of Subjects

6.4.1 Discontinuation criteria

In the event of any of the following items, the principal investigator or subinvestigator should discontinue the clinical research of the relevant subject.

1) When the subject withdraws consent
2) When the subject experiences an unacceptable adverse event
3) When iCEPS shows poor performance or the condition of the subject worsens, and the clinical research should be discontinued in the opinion of the investigator or subinvestigator
4) When ineligibility of the subject is revealed after the start of the clinical research
5) When the subject is lost to follow-up because he/she does not visit the research site
6) If the subject becomes pregnant before iCEPS is transplanted
7) Other cases where the investigator or subinvestigator considers it necessary to discontinue the clinical research

6.4.2 Procedures and follow-up for subjects who discontinue the research

Appropriate action should be taken for the subject according to the reason for discontinuation, and mandatory examination/survey at the end of the research should be completed within 7 days after discontinuation as far as practicable to evaluate the condition of the subject at the time of discontinuation. The results should be documented in the CRF.

Follow-up of the subject is continued after discontinuation of the clinical research as far as practicable, regardless of the reason for discontinuation, and the results are entered in the CRF. If a subject discontinued the research due to an adverse event, he/she should be followed up until his/her condition/laboratory values improve or stabilize as far as practicable. If a subject does not come to the research site in the course of the research, the reason and clinical course should be followed up using an appropriate method (e.g., phone calling), and the results should be documented in the medical record and CRF.

6.5 Pregnancy During the Clinical Research Period

Investigators will instruct female subjects of childbearing potential and male subjects with a partner of childbearing potential to use two or more of the contraceptive methods listed in (1) to (4) or either of the contraceptive methods listed in (5) and (6), from the date of informed consent until the end of follow-up or discontinuation date. Subjects meeting these criteria should use appropriate contraception in accordance with their physician’s instructions.

1) Use condoms
2) Use a pessary
3) Take oral contraceptives
4) Use a properly placed intrauterine device or intrauterine system

5) Male sterilization (bilateral vasectomy)
6) Female sterilization (bilateral tubal ligation)

When obtaining consent, the risks of pregnancy should be carefully explained in the informed consent form, the subject should be made clearly aware of the need to avoid pregnancy by using two or more of the methods listed in (1) to (4) or either of the methods listed in (5) and (6), and the subject's consent should be obtained with their full understanding of this information.

If a female subject becomes pregnant after informed consent but before iCEPS transplantation, the clinical research will be discontinued for that subject.

Investigators should instruct subjects to contact them in the event that a female subject or male subject's partner becomes pregnant after iCEPS transplantation. In case of such an occurrence, the investigator will promptly report the pregnancy to the principal investigator.

The investigator will discuss the risks associated with pregnancy continuation and the possible effects on the fetus with that subject or subject's partner and will provide advice. Appropriate medical care will be provided to that subject or subject's partner, and follow-up will continue until the end of the gestation period.

7. Planned Sample Size and Research Period

Planned sample size: 4 subjects

Research period: May 23, 2019 to March 31, 2024

Follow-up period: 1 year (52 weeks) after iCEPS transplantation

Additional safety monitoring period: 1 year (52 weeks) after the end of the follow-up period

*After follow-up is complete, subjects will be encouraged to make regular outpatient visits and participate in follow-up throughout their lives as much as possible so that data can be collected and appropriate measures are taken as needed. Data collected through follow-up surveys will not be included in the analysis of results for this clinical research.

[Rationale for setting]

Osaka University Hospital, the site for this research, sees approximately 100 patients with LSCD each year.

Of these, only about 10% (10 patients) would likely be eligible for the protocol treatment. If we estimate that 20% of these eligible candidates would enroll, we would expect to be able to recruit 2 subjects per year. Therefore, the target sample size was set as 4 subjects.

The additional safety monitoring period of one year (52 weeks) was set in consideration of the following.

1) Overseas research of autologous cultivated corneal epithelial cell sheet transplantation

showed that if the reconstructed corneal epithelium is stable for one year after transplantation, stability of the corneal epithelium can be maintained thereafter, confirming a sustained therapeutic effect[14).].

2) A report of long-term outcomes of autologous cultivated oral mucosal epithelial cell sheet transplantation showed that conjunctivalization, corneal opacity, and corneal neovascularization rates stabilize from one year after transplantation, with few changes[17)].

3) As an follow-up period of about 6 months is generally required after ophthalmic surgery, 52 weeks can be considered a sufficient period to confirm safety.

4) A nonclinical tumorigenicity study of iCEPS, the specific processed cell product used in this research, showed no tumorigenesis in mice for 4 months after transplantation (see "5.3 Overview of Preclinical Study Results"). For the first-in-human transplantation of iCEPS, we believe that a safety confirmation period of 1 year (52 weeks) is reasonable, considering 1) to 3) above. A additional safety monitoring period of one year will be set, but follow-up will continue as long as possible thereafter to confirm the long-term safety of iCEPS.

8. Tests/Observations and Schedule

8.1 Subject characteristics

Information on the following items will be collected at screening.

1) Date when informed consent was obtained
2) Date of birth
3) Sex
4) Causative disease of LSCD
5) Pre-existing medical conditions (ocular and non-ocular)
6) Comorbidities and their severity (ocular and non-ocular)
7) History of eye surgery
8) Information regarding iCEPS transplantation (target eye for transplant: left or right)
9) HLA type*
10) Infectious disease status

*HLA type will be obtained through secondary use of test results from another clinical research, "HLA haplotype distribution and immune response to therapeutic iPS cells derived from HLA-homozygous donors in patients with LSCD."

8.2 Slit-lamp Microscopy and Anterior Segment Photography

The anterior segment of the eye will be photographed using a slit-lamp microscope.

Observation target: Target eye for iCEPS transplantation

Observation items: (1) Safety

Incidence and severity of ocular adverse events

(2) Efficacy

LSCD stage, severity of corneal epithelial defect, degree of corneal opacification, degree of corneal neovascularization, degree of symblepharon

Timing: At screening, day of iCEPS transplantation (before transplantation, during transplantation [after removal of conjunctival tissue and immediately after transplantation]), Weeks 1*, 2, 4, 8, 16, 24, 32, 40, and 52 after iCEPS transplantation or the day of discontinuation, and Week 104 after iCEPS transplantation

* Only safety observations using slit-lamp microscopy will be performed on Week 1 after iCEPS transplantation.

8.3 Anterior Segment Optical Coherence Tomography (AS-OCT)

The anterior segment will be observed with a Tomey CASIA2 Anterior Segment OCT, Optovue RTVue-100, and Topcon DRI OCT Triton.

Observation target: Target eye for iCEPS transplantation

Observation items: (1) Safety

Incidence and severity of ocular adverse events

(2) Efficacy

Degree of corneal opacification, degree of corneal neovascularization

Timing: At screening, day of iCEPS transplantation (before transplantation), Weeks 2, 4, 8, 16, 24, 32, 40, and 52 after iCEPS transplantation or the day of discontinuation, and Week 104 after iCEPS transplantation

8.4 Visual Acuity Test

Corrected distance visual acuity will be measured using the Landolt ring chart, which is the standard visual acuity test chart specified in JIS T 7309, as well as the ETDRS visual acuity test.

Observation target: Target eye for iCEPS transplantation

Timing: At screening, day of iCEPS transplantation (before transplantation), Weeks 2, 4, 8, 16, 24, 32, 40, and 52 after iCEPS transplantation or the day of discontinuation, and Week 104 after iCEPS transplantation

8.5 Subjective Symptoms

Subjective symptoms (eye pain, foreign body sensation, lacrimation, photophobia, dryness, and discomfort) will be evaluated through patient interviews.

Observation target: Target eye for iCEPS transplantation

Timing: At screening, day of iCEPS transplantation (before transplantation), Weeks 2, 4, 8, 16, 24, 32, 40, and 52 after iCEPS transplantation or the day of discontinuation, and Week 104 after iCEPS transplantation

8.6 QOL

The QOL related to visual function will be evaluated using the Japanese version of the NEI VFQ-25 (v1.4).

Timing: At screening, day of iCEPS transplantation (before transplantation), Week 52 after iCEPS transplantation or the day of discontinuation

8.7 Laboratory Tests

1) Blood tests

Blood will be collected. (1) and (2) will be used to observe abnormal fluctuations in laboratory values. Results of (3) will be used as a reference to diagnose rejection. (4) will be used to check for infectious diseases. Results of (5) will be used as an indicator to rule out advanced cancer. (6) will measure blood levels of immunosuppressive agents and be used as an indicator for dose determination. (Numbers in parentheses indicate the volume of blood collected)

(1) Hematology (8 mL for (1) and (2))

Parameters: Red blood cell count, white blood cell count, differential white blood cell count (neutrophils, eosinophils, basophils, and lymphocytes), hemoglobin, hematocrit, and platelet counts

Period A: Subjects receiving immunosuppressive agents as specified in "6.2 Administration of Immunosuppressive Agents After iCEPS Transplantation"

At screening, day of iCEPS transplantation (before transplantation), Weeks 2, 4, 8, 12, 16, 20, 24, 28, 32, 36, 40, 44, 48, and 52 after iCEPS transplantation or the day of discontinuation

Period B: Subjects not receiving immunosuppressive agents as specified in "6.2 Administration of Immunosuppressive Agents After iCEPS Transplantation"

At screening, day of iCEPS transplantation (before transplantation), Weeks 2, 4, 12, 24, 36, and 52 after iCEPS transplantation or the day of discontinuation

(2) Biochemistry

Parameters: AST (GOT), ALT (GPT), serum total protein, serum creatinine, and C-reactive protein (CRP)

Period A: Subjects receiving immunosuppressive agents as specified in "6.2 Administration of Immunosuppressive Agents After iCEPS Transplantation"

At screening, day of iCEPS transplantation (before transplantation), Weeks 2, 4, 8, 12, 16, 20, 24, 28, 32, 36, 40, 44, 48, and 52 after iCEPS transplantation or the day of discontinuation

Period B: Subjects not receiving immunosuppressive agents as specified in "6.2 Administration of Immunosuppressive Agents After iCEPS Transplantation"

At screening, day of iCEPS transplantation (before transplantation), Weeks 2, 4, 12, 24, 36, and 52 after iCEPS transplantation or the day of discontinuation

(3) Immunoserology

Parameter: Mixed lymphocyte reaction

(Screening: 25 mL, non-screening days: 45 mL)

Corneal epithelial-specific antibody identification test (1 mL)

Timing: At screening, day of iCEPS transplantation (before transplantation), Weeks 2, 4, 12, 24, 36, and 52 after iCEPS transplantation or the day of discontinuation

*Will be performed as necessary at times not listed above.

*The dose may be reduced if the subject's physical or general condition is poor.

(4) Infectious diseases (10 mL)

Parameters: HBs antigen, HBs antibody, HBc antibody, HCV antibody, HCV-RNA, HIV antibody

Timing: At screening

(5) Tumor marker measurement (3 mL)

Parameters: CEA, CA19-9, CA125, CA15-3

Timing: At screening, Weeks 4, 12, 24, 36, and 52 after iCEPS transplantation or the day of discontinuation

(6) Trough level measurement (2 mL)

Targets: Subjects receiving immunosuppressive agents as specified in "6.2 Administration of Immunosuppressive Agents After iCEPS Transplantation"

Timing: At Weeks 2, 4, 8, 12, 16, 20, 24, 28, 32, 36, 40, 44, 48, and 52 after iCEPS transplantation or the day of discontinuation

A blood sample for storage (10 mL) will be collected at the time of blood collection on the day of iCEPS transplantation (before transplantation).

2) Urinalysis
A urinalysis is performed in women of childbearing potential.
Timing: At screening, day of iCEPS transplantation (before transplantation)

8.8 Histopathological Tests

Pathological diagnosis (biopsy tissue diagnosis) will be performed if an obvious abnormality is observed in the corneal epithelium on slit-lamp microscopy and supplementary testing in any visit from the day of iCEPS transplantation (during transplantation) until Week 52 after iCEPS transplantation or the date of discontinuation.

8.9 X-ray CT Scan

If histopathology shows a neoplastic lesion at the iCEPS transplantation site, an X-ray CT scan will be performed to search for systemic malignancy.

8.10 Adverse Events

Adverse events occurring from the day of iCEPS transplantation (during transplantation) to Week 52 after iCEPS transplantation or the date of discontinuation will be evaluated.

8.11 Acceptable Range of Test/Observation Schedule

Visit	Timing	Acceptable range
	Screening	Within −56 days of enrollment
1	Day of iCEPS transplantation (before transplantation)	(1) Day of transplantation only: Hematological tests, collection of blood sample for storage<br>(2) Within −4 days of the day of transplantation: Slit lamp microscopy and anterior segment photography,<br>anterior segment optical coherence tomography (AS-OCT),<br>visual acuity tests, subjective symptoms, QOL,<br>urinalysis
2	Week 1 after iCEPS transplantation	Only on the scheduled day
3	Week 2 after iCEPS transplantation	Within ±3 days of the scheduled day
4	Week 4 after iCEPS transplantation	Within ±4 days of the scheduled day

5	Week 8 after iCEPS transplantation	Within ±7 days of the scheduled day
6	Week 12 after iCEPS transplantation	Within ±14 days of the scheduled day
7	Week 16 after iCEPS transplantation	Within ±14 days of the scheduled day
8	Week 20 after iCEPS transplantation	Within ±14 days of the scheduled day
9	Week 24 after iCEPS transplantation	Within ±14 days of the scheduled day
10	Week 28 after iCEPS transplantation	Within ±14 days of the scheduled day
11	Week 32 after iCEPS transplantation	Within ±14 days of the scheduled day
12	Week 36 after iCEPS transplantation	Within ±14 days of the scheduled day
13	Week 40 after iCEPS transplantation	Within ±14 days of the scheduled day
14	Week 44 after iCEPS transplantation	Within ±14 days of the scheduled day
15	Week 48 after iCEPS transplantation	Within ±14 days of the scheduled day
16	Week 52 after iCEPS transplantation	Within ±14 days of the scheduled day
	Follow-up end date	Date performed
	Day of discontinuation	Within +7 days of the day of discontinuation
	Week 104 after iCEPS transplantation	Within ±28 days of the scheduled day

8.12 Test/Observation Schedule

Table 1

Timing / Tests/observations	Before enrollment	Day of transplantation			After transplantation (follow-up period)							
		Visit 1			Visit 2	Visit 3	Visit 4	Visit 5	Visit 6	Visit 7	Visit 8	Visit 9
	Screening	Day 0			Week 1 (Days 3–7)	Week 2 (Day 14)	Week 4 (Day 28)	Week 8 (Day 56)	Week 12 (Day 84)	Week 16 (Day 112)	Week 20 (Day 140)	Week 24 (Day 168)
Acceptable range	Within −56 days of enrollment					±3 days	±4 days	±7 days	±14 days	±14 days	±14 days	±14 days
		Before transplant-ation	During transplantation: After removal of conjunctival tissue	During transplantation: Immediately after transplantation								
Subject characteristics	○											
Slit-lamp microscopy and anterior segment photography	○	○*3	○*5	○*5	○*6	○	○	○		○		○
Anterior segment optical coherence tomography	○	○*3				○	○	○		○		○
Visual acuity test	○	○*3				○	○	○		○		○
Subjective symptoms	○	○*3				○	○	○		○		○
QOL	○	○*3										
Blood tests: Hematology	○	○*4				○	○	☆	○	☆	☆	○
Blood tests: Biochemistry	○	○*4				○	○	☆	○	☆	☆	○
Blood tests: Immunoserology	○	○*4				○	○		○			○
Blood tests: Tests for infectious diseases	○											
Blood tests: Tumor marker measurement*1	○						○		○			○
Blood tests: Trough level measurement						☆	☆	☆	☆	☆	☆	☆
Collection of blood sample for storage		○*4										
Urinalysis*2	○	○*3										

	Timing	After transplantation (follow-up period)								AM period*7
Tests/observations		Visit 10	Visit 11	Visit 12	Visit 13	Visit 14	Visit 15	Visit 16	Day of discontinuation	
		Week 28 (Day 196)	Week 32 (Day 224)	Week 36 (Day 252)	Week 40 (Day 280)	Week 44 (Day 308)	Week 48 (Day 336)	Week 52 (Day 364)		Week 104 (Day 728)
Acceptable range		±14 days	±14 days	±14 days	±14 days	±14 days	±14 days	±14 days	Within +7 days	±28 days
Slit-lamp microscopy and anterior segment photography			○		○			○	○	○
Anterior segment optical coherence tomography			○		○			○	○	○
Visual acuity test			○		○			○	○	○
Subjective symptoms			○		○			○	○	○
QOL								○	○	
Blood tests	Hematology	☆	☆	○	☆	☆	☆	○	○	
	Biochemistry	☆	☆	○	☆	☆	☆	○	○	
	Immunoserology			○				○	○	
	Tests for infectious diseases									
	Tumor marker measurement*1			○				○	○	
	Trough level measurement	☆	☆	☆	☆	☆	☆	☆	☆	

During the clinical research period, monitoring of systemic adverse events will be conducted as needed through blood tests and patient interviews.

Pathological diagnosis (biopsy tissue diagnosis) will be performed if an obvious abnormality is observed in the corneal epithelium on slit-lamp microscopy. If a neoplastic lesion is observed, an X-ray CT scan will be performed.

○: To be performed

☆: To be performed in subjects receiving immunosuppressive agents as specified in "6.2 Administration of Immunosuppressive Agents After iCEPS Transplantation"

*1: If values after iCEPS transplantation are higher than screening values and are abnormal, an oncologist will collaborate in the subject's care.

*2: Urine pregnancy tests will be performed for women of childbearing potential.

*3: To be performed within −4 days of iCEPS transplantation

*4: To be performed on the day of iCEPS transplantation

*5: Anterior segment photographs should be obtained by extracting frames from surgical videos.

*6: Only slit-lamp microscopy will be performed.

*7: A one-year Additional safety Monitoring (AM) period will be performed after the end of the 52-week follow-up period.

9. Definition of Efficacy and Safety Evaluations and Endpoints

9.1 Primary Endpoint

Safety will be evaluated by collecting adverse event data.

Evaluation period: From the day of iCEPS transplantation (during transplantation) to Week 52 after iCEPS transplantation or the date of discontinuation

Recording method: The subinvestigator acting as the subject's physician will record the details of each adverse event in the CRF (see "10.4 Response and Follow-up Investigation After Occurrence of Adverse Events"). The subinvestigator and physician-evaluator will make determinations regarding rejection and uncontrollable rejection.

[Rationale for setting]

The endpoint was set to evaluate the safety of the protocol treatment by assessing the overall incidence of adverse events.

9.2 Secondary Endpoints

The following efficacy items will be evaluated. Evaluation results for each item will be documented in the CRF.

1) LSCD stage

The LSCD stage will be determined by slit-lamp microscopy using the severity classification for LSCD (Fig. 1).

Target of evaluation: Target eye for iCEPS transplantation

Timing: At screening, day of iCEPS transplantation (before transplantation), and Weeks 2, 4, 8, 16, 24, 32, 40, and 52 after iCEPS transplantation or the discontinuation date

Method for recording determination: The consensus of the subinvestigator and physician-evaluator who made the determination will be recorded on a worksheet.

2) Severity of corneal epithelial defect

The severity of corneal epithelial defect will be determined using slit-lamp microscopy.

[Criteria of severity]

Grade 0 : No corneal epithelial defect

Grade 1 : Corneal epithelial defect in <1/4 of the surface of the cornea

Grade 2 : Corneal epithelial defect in ≥1/4 and <1/2 of the surface of the cornea

Grade 3 : Corneal epithelial defect in ≥1/2 of the surface of the cornea

Target of evaluation: Target eye for iCEPS transplantation

Timing: At screening, day of iCEPS transplantation (before transplantation), and Weeks 2, 4, 8, 16, 24, 32, 40, and 52 after iCEPS transplantation or the discontinuation date

Method for recording determination: The consensus of the subinvestigator and physician-evaluator who made the determination will be recorded on a worksheet.

3) Subjective symptoms

The severity of subjective symptoms (eye pain, foreign body sensation, lacrimation, photophobia, dryness, and discomfort) will be evaluated through patient interviews.

[Severity]

0: No symptoms

1: Mild symptoms

2: Moderate symptoms

3: Severe symptoms

4: Markedly severe symptoms

Target of evaluation: Target eye for iCEPS transplantation

Timing: At screening, day of iCEPS transplantation (before transplantation), and Weeks 2, 4, 8, 16, 24, 32, 40, and 52 after iCEPS transplantation or the discontinuation date

Method for recording results: The subinvestigator who conducted the interview will record the subject’s answers in their medical record.

4) Corrected distance visual acuity

(1) Visual acuity will be measured using the standard visual acuity table specified in JIS T 7309 (Landolt ring chart) converted to LogMAR.

(2) Visual acuity will be measured using the ETDRS visual acuity test chart.

Target of evaluation: Target eye for iCEPS transplantation

Timing: At screening, day of iCEPS transplantation (before transplantation), and Weeks 2, 4, 8, 16, 24, 32, 40, and 52 after iCEPS transplantation or the discontinuation date

Method for recording results: The person who conducted the ophthalmological examination will record the results in the subject’s medical record.

5) QOL

The QOL related to visual function will be evaluated using the Japanese version of the NEI VFQ-25 (v1.4).

Timing: At screening, day of iCEPS transplantation (before transplantation), Week 52 after iCEPS transplantation or the day of discontinuation

Method for recording results: The subinvestigator who conducted the QOL survey or the clinical research coordinator will record the results in the CRF supplement (QOL questionnaire form).

6) Degree of corneal opacification

The cornea was divided radially into eight sections, and the degree of corneal opacification in each section will be determined using slit-lamp microscopy.

[Criteria of severity]

Grade 0: The cornea is transparent, and the iris can be observed in detail.

Grade 1: Details of the iris can be partially observed.

Grade 2: The iris details cannot be clearly observed, and the rim of the pupil is scarcely observed.

Grade 3: Neither the iris nor rim of the pupil could be observed.

Target of evaluation: Target eye for iCEPS transplantation
Timing: At screening, day of iCEPS transplantation (before transplantation), and Weeks 2, 4, 8, 16, 24, 32, 40, and 52 after iCEPS transplantation or the discontinuation date
Method for recording determination: The consensus of the subinvestigator and physician-evaluator who made the determination will be recorded on a worksheet.

7) Degree of corneal neovascularization
The cornea was divided radially into eight sections, and the degree of corneal neovascularization will be determined using slit-lamp microscopy and OCT.

[Criteria of severity]

Grade 0 : No neovascularization

Grade 1 : Neovascularization around the cornea

Grade 2 : Neovascularization to the rim of the pupil

Grade 3 : Neovascularization exceeding the rim of the pupil

Target of evaluation: Target eye for iCEPS transplantation
Timing: At screening, day of iCEPS transplantation (before transplantation), and Weeks 2, 4, 8, 16, 24, 32, 40, and 52 after iCEPS transplantation or the discontinuation date
Method for recording determination: The consensus of the subinvestigator and physician-evaluator who made the determination will be recorded on a worksheet.

8) Degree of symblepharon
The degree of symblepharon will be determined using slit-lamp microscopy.

[Criteria of severity]

Grade 0: No symblepharon

Grade 1: Symblepharon limited to the surface of the conjunctiva

Grade 2: Symblepharon <1/2 of the surface of the cornea

Grade 3: Symblepharon ≥1/2 of the surface of the cornea

Target of evaluation: Target eye for iCEPS transplantation
Timing: At screening, day of iCEPS transplantation (before transplantation), and Weeks 2, 4, 8, 16, 24, 32, 40, and 52 after iCEPS transplantation or the discontinuation date
Method for recording determination: The consensus of the subinvestigator and physician-evaluator who made the determination will be recorded on a worksheet.

[Rationale for setting]

1) It is expected that the condition of LSCD is alleviated by transplantation of iCEPS.
2) iCEPS transplantation is expected to reconstruct the corneal epithelium on the surface of the eye.
3) It is known that subjective symptoms associated with LSCD are caused by conjunctivalization of the corneal epithelium. It is expected that subjective

symptoms can be alleviated when the corneal epithelium is reconstructed after transplantation of iCEPS.

4) Visual impairment associated with LSCD is caused by conjunctivalization of the corneal epithelium. It is expected that visual acuity is improved after reconstruction of the transparent corneal epithelium after transplantation of iCEPS.
5) To evaluate improvement in QOL associated with alleviation of subjective symptoms and improvement in visual acuity after corneal epithelial reconstruction.
6) To evaluate corneal opacity after transplantation of iCEPS and to evaluate its relationship with improvement in visual acuity.
7) Corneal neovascularization in LSCD is associated with conjunctivalization of the corneal epithelium. It is expected that corneal neovascularization can be controlled by corneal epithelial reconstruction after transplantation of iCEPS.
8) Symblepharon is seen as a complication in some patients with LSCD. This condition is treated at the time of transplantation, and it is expected that recurrence of symblepharon can be prevented when the corneal epithelium is reconstructed by transplantation of iCEPS.

9.3 Decision Items for Individual Endpoints

Subinvestigators and physician-evaluators will make determinations regarding decision items for the endpoints specified in “9.1 Primary Endpoint” and “9.2 Secondary Endpoints” at each evaluation time point. The final determination will be made by consensus.

9.4 Midterm Evaluation

Subinvestigators will conduct a midterm evaluation of the protocol treatment in each subject at 24 weeks after the second subject undergoes transplantation. Midterm evaluation will consist of evaluation for rejection. The decision will be made by subinvestigators and a physician-evaluator not directly involved in the conduct of this research.

1) Determination of rejection status

   Rejection status is determined comprehensively based on findings (1) through (3) below.

   (1) Corneal stromal edema
   (2) Ciliary injection
   (3) Corneal epithelial defect

   Corneal infection and flare-up of the primary disease should be denied.

2) Determination of uncontrollable rejection

   Rejection is considered uncontrolled if treatment for rejection produces no decrease in stromal edema in the cornea, ciliary injection, or corneal epithelial defect.

Based on the results of the above determination, the principal investigator and subinvestigators will decide the HLA type selection plan for the third and subsequent iCEPS transplantation cases and whether or not to administer immunosuppressive agents after transplantation, as described below.

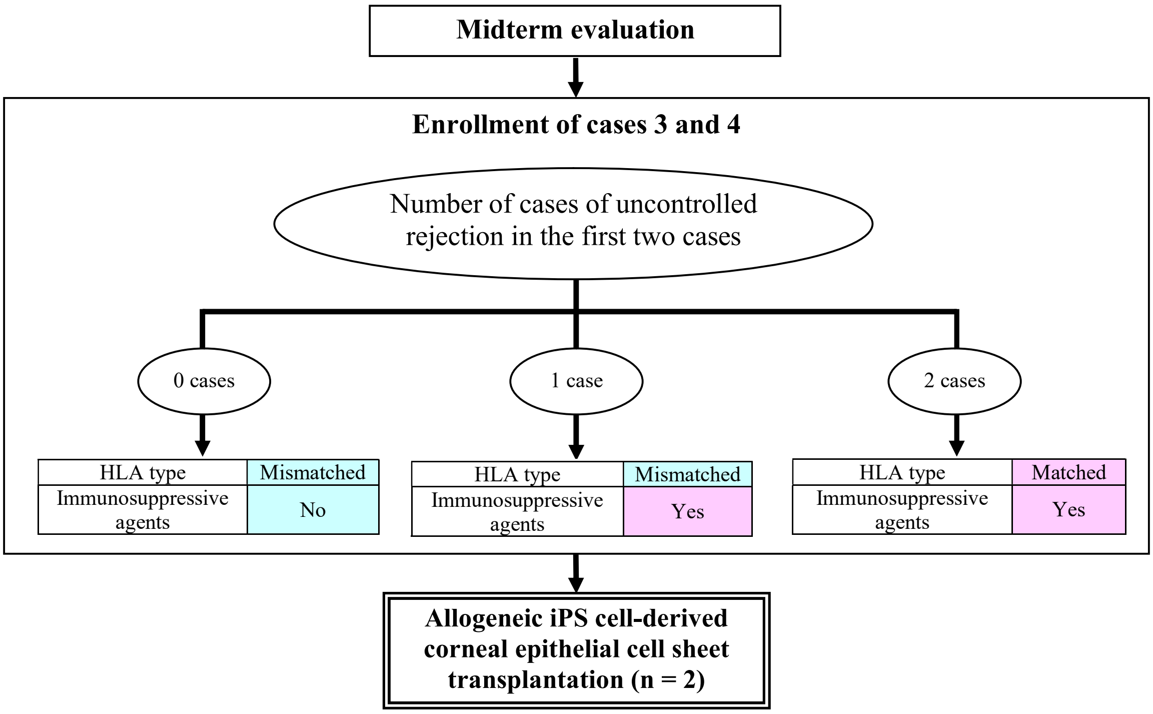

9.5 Final Evaluation

The principal investigator, subinvestigators, and physician-evaluator not directly involved in the conduct of this research will conduct a final evaluation of the protocol treatment at the end of follow-up for all subjects or after discontinuation of the research.

9.6 Safety Monitoring Committee

The principal investigator will establish a Safety Monitoring Committee. The committee will consist of several external evaluation committee members. Committee members will be selected from ophthalmologists who are independent of the parties involved in the conduct of this research and who have academic knowledge in relevant research areas and knowledge of regenerative medicine.

At the request of the principal investigator, the Safety Monitoring Committee will review and provide their opinion on the following items.

1) Causality of serious adverse events
2) The conduct of this clinical research and the impact of information obtained from reports of research on similar treatments and other sources on the continuation of the entire clinical research (e.g., if a neoplastic lesion developed)

3) Need for protocol revision

Discussions will be conducted by appropriate means, such as convening committee meetings, sending internal memos, and hearing opinions by means such as phone, fax, and e-mail.

10. Reporting Adverse Events

10.1 Definitions of Adverse Events, Serious Adverse Events, Malfunctions, Diseases, and Related Terms

10.1.1 Adverse events

An adverse event is any unfavorable or unintended sign (including abnormal laboratory values), symptom, or disease that occurs during the research period, whether or not it is considered related to the protocol treatment. In this research, an adverse event is defined as any of the above signs, symptoms, or diseases that a subinvestigator considers to be clinically problematic.

10.1.2 Serious adverse events

A serious adverse event is any adverse event that:

1) results in death;
2) is life-threatening;
   The term "life-threatening" here refers to an event in which the subject was at risk of death at the time of the event; it does not refer to an event that hypothetically might have caused death if it were more severe.
3) requires hospitalization or results in prolongation of existing hospitalization;
4) results in persistent or significant disability/incapacity;
5) is a congenital anomaly/birth defect; or
6) is a medically important event.

The term "a medically important event" here refers to an important medical event that might not be immediately life-threatening or result in death or hospitalization but might jeopardize the subject or might require intervention to prevent one of the other outcomes listed in the aforementioned definition.

10.1.3 Malfunctions

A malfunction is any issue with the function of the specific processed cell product or adverse effect of the cells on the human body, by a broad scope of definition, regardless of the stage in which it arose, whether it was during production, supply, storage, or use.

10.1.4 Diseases or the like

"Diseases or the like" indicates disease, disability, death, or infectious disease that is suspected to be caused by provision of the regenerative medicine product as described in the regenerative medicine provision plan.

10.2 Assessment of Adverse Events

The severity of adverse events will be determined using the JCOG Japanese translation of the Common Terminology Criteria for Adverse Events v4.0 (CTCAE v4.0 - JCOG)[24]. However, adverse events localized to the eye will be classified into three levels of severity (mild, moderate, or severe) as follows:

Severity assessment of adverse events localized to the eye:

Mild : Signs or symptoms that are easily bearable,
Moderate : Signs or symptoms that interfere with daily activities, or
Severe : Signs or symptoms that hinder work or daily activities.

10.3 Criteria for Causality Assessment

The causal relationship with the protocol treatment (allogeneic iPS cell-derived corneal epithelial cell sheet transplantation) will be assessed according to the following criteria.

Causal relationship	Criteria for causality assessment
Not related	There is sufficient information to show that the event is unrelated to the protocol treatment, or the event can be sufficiently explained by the patient's known clinical history.
Cannot be denied	The event is undeniably correlated with the protocol treatment and cannot be explained by other factors such as the primary disease or a comorbidity, concomitant drug, or concomitant therapy.
Related	The event is correlated with the protocol treatment, and cannot be explained by other factors such as the primary disease or a comorbidity, concomitant drug, or concomitant therapy. When the protocol treatment is discontinued, the clinical course (e.g., reduced severity or disappearance of the event) indicates that the event was caused by the protocol treatment.

When the causal relationship with the protocol treatment (allogeneic iPS cell-derived corneal epithelial cell sheet transplantation) is determined to be "related" or "cannot be denied," the cause will be determined as follows.

1) Caused by transplantation surgery/procedures
2) Caused by the transplanted specific processed cell product (i.e., iCEPS)
3) Caused by general anesthesia
4) Other

10.4 Response and Follow-up Investigation After Occurrence of Adverse Events

The investigator or subinvestigator shall provide appropriate first aid to adverse events occurring in the subject participating in the clinical research, pay attention to ensuring the safety of the subject, and take appropriate measures such as receiving a diagnosis by a specialist doctor as necessary, and endeavor to investigate the cause therein.

In addition, the adverse event name, severity, onset date, outcome, outcome date, severity, causal relationship with the protocol treatment, rationale for causality determination, and, if necessary, details of treatment and progress. If the diagnosis name is identified from the signs or symptoms, give priority to the individual signs and symptoms. If the diagnosis name is not identified from the signs or symptoms, each sign and symptom is filled in as an individual adverse event.

The term of adverse events used by subinvestigators is replaced with standard terms using MedDRA/J for aggregation and analysis. The medical validity of the rereading operation is determined by the investigator acting as the subject's physician.

As much as possible, follow-up investigations will be conducted on adverse events that have occurred, particularly events in which causal relationships with the protocol treatment cannot be denied. Adverse events that develop during the clinical research period are tracked until they are recovered or determined to be clinically unnecessary.

10.5 Measures for Serious Adverse Events and Malfunctions

If an investigator or research collaborator becomes aware of the occurrence of a serious adverse event (excluding diseases or the like) or malfunction, they will take necessary measures, such as explaining the event or malfunction to the subject, and reporting the information promptly to the principal investigator. If the principal investigator becomes aware of the occurrence of a serious adverse event or malfunction, he/she will promptly report it to the head of the research site using Reference Form 1, "Report on Serious Adverse Events and Malfunctions," and take appropriate action. In addition, the principal investigator will promptly share information regarding the occurrence of the serious adverse event or malfunction with investigators and other personnel involved in the conduct of the clinical research.

10.6 Measures for Diseases or the Like

If an investigator becomes aware that a subject has developed a disease or the like, they will promptly report this information to the head of the research site and the principal investigator.

When the head of the research site or the principal investigator receives a report, they will notify applicable investigators to discontinue the clinical research or take other necessary measures. In addition, the cell culturing and processing facility must be promptly notified of the situation that occurred and the measures taken.

1) Reporting to the Certified Committee for Regenerative Medicine

When the head of the research site becomes aware of any of the matters listed below, he/she will report the matter to the Certified Committee for Regenerative Medicine using Appendix Form 1 "Disease Report (for Committee Report)" within the applicable period specified in (1) to (3) below.

(1) Occurrence of disease or the like meeting any of the following criteria that is either suspected to be caused by provision of the regenerative medicine product or result from an infectious disease suspected to be caused by the provision of the regenerative medicine product: 7 days

i. Results in death

ii. Is life-threatening

(2) Occurrence of disease or the like meeting any of the following criteria that is either suspected to be caused by provision of the regenerative medicine product or result from an infectious disease suspected to be caused by the provision of the regenerative medicine product: 15 days

i. Requires hospitalization or results in prolongation of existing hospitalization

ii. Results in disability/incapacity

iii. Poses a risk of disability/incapacity

iv. Is serious

v. Is a congenital disease or anomaly in offspring

(3) Occurrence of diseases or the like suspected to be caused by provision of the regenerative medicine product or by an infectious disease suspected to be caused by provision of the regenerative medicine product (excluding (1) and (2)): Within 10 days of the end of the applicable period every 60 days from the date of submission of the Regenerative Medicine Provision Plan to the Minister of Health, Labour and Welfare

When the head of the research site receives an opinion from the Certified Committee for Regenerative Medicine, the head of the site will report changes made to the Regenerative Medicine Provision Plan and other measures taken based on that opinion back to the Certified Committee for Regenerative Medicine.

2) Reporting to the Minister of Health, Labour and Welfare

When the head of the research site becomes aware of (1) or (2) listed in 1) above, the head of the site will report the matter to the Minister of Health, Labour and Welfare using Appendix Form 2 "Disease Report (for MHLW Report)" within the applicable period specified in (1) or (2) in 1).

10.7 Expected Adverse Events

10.7.1 Adverse events caused by transplantation surgery/procedures

1) Conjunctival hyperemia
   Treatment: Topical or systemic anti-inflammatory agents
2) Subjective symptoms (eye pain, foreign body sensation, lacrimation, photophobia, dryness, and discomfort)
   Treatment: Eye drops, eye ointment, or oral medication for serious cases
3) Superficial punctate keratopathy
   Treatment: Eye drops or eye ointment administered locally for serious cases
4) Corneal epithelial defect
   Treatment: Eye drops or eye ointment for serious cases
5) Symblepharon
   Treatment: Surgical release of adhesions for serious cases
6) Conjunctivalization
   Treatment: Eye drops or eye ointment as needed for serious cases
7) Infectious keratitis
   Treatment: Antibiotics, antivirals, or antifungals, and surgical treatment as needed
8) Increased intraocular pressure
   Treatment: Local or systemic antiglaucoma agents, and surgical treatment as needed
9) Cataract
   Treatment: Surgical treatment for serious cases
10) Corneal keratinization
   Treatment: Removal of corneal keratinization for serious cases
11) Endophthalmitis
   Treatment: Antibiotics, antivirals, or antifungals, and surgical treatment as needed

10.7.2 Adverse events caused by the specific processed cell product (iCEPS)

1) Allergic reaction due to use of materials of animal origin (bovine, porcine, or rodent)
   Treatment: Systemic allergic reactions should be treated by systemic antiallergic agents and corticosteroids, and shock should be treated by vasopressors and bronchodilators.
2) Infectious disease due to use of materials of human and animal origin (bovine, porcine, or rodent)
   Treatment: If infectious keratitis is suspected, identification of the causative microorganism by culture of a scraped sample of the lesion and treatment by means such as topical/systemic antibiotics or graft removal may be necessary.
3) Immunological rejection
   Treatment: Immunosuppressive agents and corticosteroids may be required.
4) Development of a neoplastic lesion
   (1) Local (at site of iCEPS transplantation)

Treatment: Although this is considered extremely unlikely, if it does occur, appropriate treatment should be given based on the type of lesion.

(2) Systemic

Treatment: Development outside the local area is unlikely, but if it does occur, appropriate treatment should be given based on the type of lesion.

10.7.3 Adverse events caused by general anesthesia

Adverse events caused by procedures such as tracheal intubation and anesthetic use may include the following:

Dental injury, pharyngeal injury, hoarseness, bronchospasm (asthma attack), urticaria/shock symptoms, malignant hyperthermia, aspiration pneumonia, etc.

11. Discontinuation of the Entire Clinical Research

11.1 Discontinuation Criteria

The principal investigator will discontinue the entire clinical research in the following cases:

1) The principal investigator determines that there is a safety issue with the protocol treatment
2) Development of the specific processed cell product (iCEPS) is discontinued
3) The research is discontinued based on guidance or orders from regulatory authorities, the opinion of the Certified Committee for Regenerative Medicine, or the judgment of the head of the research site

11.2 Discontinuation Procedures

If the decision is made to discontinue the entire clinical research, the principal investigator or subinvestigator will promptly notify the subjects and provide appropriate treatment. In addition, they will carry out necessary procedures in accordance with “16.5 Report of Discontinuation.”

12. Case Report Forms

12.1 Preparation, Modification, and Correction of Case Report Forms

1) The principal investigator or a subinvestigator will prepare CRFs for enrolled subjects.
2) If the clinical research coordinator is assisting in preparing CRFs, they should only transcribe from source documents, within the scope not requiring medical judgment, and should be supervised by the principal investigator or subinvestigator.
3) Details are described separately in “Guidance on Preparation, Modification, and Correction of Case Report Forms.”

12.2 Review of Case Report Forms

1) When a CRF is prepared by a subinvestigator, the principal investigator will inspect the contents of the form to confirm that there are no problems before submission to the Data Center and will then sign or stamp the form and submit it to the Data Center.
2) The principal investigator will ensure that CRFs submitted to the Data Center are accurate, complete, legible, and submitted in a timely manner, and that subject identification codes and registration numbers are used to identify subjects.

12.3 Submission Deadlines for Case Report Forms

The principal investigator, subinvestigators, and research collaborators will prepare a CRF promptly after the completion of each visit or at research discontinuation for the subject in question. The principal investigator will then sign or stamp the form, submit it to the Data Center, and retain a copy.

12.4 Specification of Case Report Form Entries That Are Regarded as Source Documents

Information on the following 1) to 7) entered directly into CRFs can be used as source documents.

1) Severity of comorbidities
2) Details of concomitant drugs and therapies
3) Adverse event status and details
4) Need for histopathological testing
5) Need for X-ray CT scan
6) Reason for discontinuation
7) Other comments of the investigator and subinvestigator

13. Statistical Considerations

13.1 Definitions of Analysis Sets

1) Safety analysis set

   Includes all enrolled subjects except those meeting the criteria below:

   (1) Did not undergo iCEPS transplantation

   (2) Did not undergo any safety observations

2) Efficacy analysis set

   Includes all enrolled subjects except those meeting the criteria below:

   (1) Did not undergo iCEPS transplantation

   (2) Did not undergo any efficacy observations after iCEPS transplantation

(3) Major protocol violation

13.2 Analyses and Methods

All statistical analyses will be performed after data lock for all subjects.

13.2.1 Disposition of subjects

The number of enrolled subjects, number of ineligible subjects, number of post-enrollment deviations, number included in analysis sets, and number of subjects who discontinued will be tabulated. Reasons will be given for subjects who had post-enrollment deviations or discontinued.

13.2.2 Subject characteristics

Subject characteristics will be presented in a listing table.

13.2.3 Primary endpoint (safety)

Adverse events for which data were collected will be listed for each subject (event name, onset date, outcome date, duration, outcome, severity, seriousness, causal relationship with the protocol treatment, and rationale for causality determination). The number of subjects who experienced the event and the number of events will also be shown for each adverse event. Duration is defined as “outcome date − onset date + 1 (day).” Serious adverse events will be tabulated in a similar fashion.

Neoplastic lesion status and rejection status at each time point will also be listed, along with details of relevant laboratory results.

13.2.4 Secondary endpoints (efficacy)

A time course of severity will be shown in a table and line graph for the following items defined in “9.2 Secondary Endpoints”: 1) LSCD stage, 2) severity of corneal epithelial defect, 3) subjective symptoms, 6) degree of corneal opacification, 7) degree of corneal neovascularization, and 8) degree of symblepharon. For “4) Corrected distance visual acuity,” measured values and the magnitude of change from baseline over time will be shown in a table and line graph. For “5) QOL,” the 12-item subscale scores on the NEI VFQ-25 will be calculated, and the overall score will be calculated from 10 subscales excluding “general health” and “driving.” In addition, summary statistics for overall score at baseline and at Week 52 after iCEPS transplantation or the discontinuation date will be calculated and evaluated. Baseline is defined as the day of transplantation (before transplantation).

13.3 Significance Level To Be Used

No estimations or tests that would require setting of confidence coefficients or significance levels will be used in this research. Instead, descriptive methods will be used in analysis.

13.4 Procedures for Handling Missing, Rejected, and Abnormal Data

Missing data will not be imputed. Handling of deviations or abnormal data will be determined at a Clinical Case Conference prior to data lock.

13.5 Procedures for Changing the Analysis Plan

If a change or addition to the analysis plan is made after the start of the research, the protocol will be revised. The circumstances that led to the change or addition to the analysis plan will be described in the clinical study report (CSR).

14. Ethical Considerations

14.1 Compliance with Regulatory Requirements

This clinical research will be conducted in compliance with the ethical principles of the Declaration of Helsinki (revised October 2013), the Act on the Safety of Regenerative Medicine (Act No. 85 of 2013), and other applicable laws and regulations, as well as this protocol.

14.2 Informed Consent

14.2.1 Preparation of the patient information sheet and informed consent form

The principal investigator will prepare the patient information sheet and informed consent form for this research. The Certified Committee for Regenerative Medicine will review these documents prior to the start of the research. The patient information sheet will include, at a minimum, the explanations of the following:

1) Title of the research and the fact that a Regenerative Medicine Provision Plan has been submitted to the Minister of Health, Labour and Welfare
2) Name of the research site and the names of the administrator, principal investigator, and subinvestigators at that site
3) That the protocol treatment is investigational
4) Objectives and significance of the research
5) Research methods
6) Expected duration of subject participation and expected number of subjects
7) Information on cells used in the research

8) The reason why the subject was selected
9) Expected benefits and disadvantages
10) Availability of alternative treatments, descriptions of those treatments, and comparison of potential benefits and disadvantages
11) That the subject's participation in the research is voluntary
12) That the subject may refuse to participate or withdraw from the research even after giving consent, without penalty or loss of benefits to which the subject is otherwise entitled to, and that their previous treatment will be continued
13) That the subject may withdraw consent to participate in the research at any time
14) That the subject will be informed in a timely manner if information that may affect the subject's willingness to continue participation in the research is obtained
15) That if the subject is unable to sign the informed consent form due to visual impairment, a designated representative must sign the form on the subject's behalf
16) That if the subject is unable to personally read the patient information sheet due to visual impairment, a witness must sign the witness section of the informed consent form
17) How information from the research will be published
18) That the subject or a designated representative may obtain and view the research protocol and other materials related to the conduct of the research on request, and the method of obtaining or viewing such materials
19) Compensation available to the subject in the event of research-related health injury
20) Protection of personal information
21) Methods for storage and disposal of samples and records
22) Costs associated with conducting the research
23) Conflicts of interest
24) Ownership of research results
25) System for handling complaints and inquiries
26) That research results may be presented at academic conferences and similar settings, with personal information kept confidential
27) That the monitors, auditors, and Certified Committee for Regenerative Medicine will be granted access to samples and data pertaining to the subject, within the necessary scope, while maintaining the confidentiality of the subject
28) Provision of medical care to subjects after the research period
29) Handling of research results (including incidental findings) pertaining to a subject when significant findings regarding the subject's health or inheritable genetic characteristics are obtained during the course of the research

30) Matters to be reviewed by the Certified Committee for Regenerative Medicine assigned to review this research, as well as other matters regarding the Certified Committee for Regenerative Medicine of this research
31) That materials of animal origin (bovine, porcine, or rodent) are used for manufacturing iCEPS and, although safety measures against transmission of infections will be taken, the risk of infections from these materials cannot be completely eliminated
32) That iCEPS will not be produced or dispensed if it is found to be contaminated during production or does not conform to test criteria during the production process or to the specifications of iCEPS. In such a case, the iCEPS transplantation date may be postponed or a change to another therapy may be made upon discussion with the principal investigator or subinvestigator.
33) That if a genetic abnormality is found in the genetic analysis performed for reference purposes, the subject will be informed, more careful post-treatment follow-up will be conducted, and appropriate measures will be taken

14.2.2 Revision of the patient information sheet and informed consent form

If the principal investigator becomes aware of matters concerning the quality, efficacy, or safety of the specific processed cell product (iCEPS) or other information important for proper conduct of the research, the principal investigator will revise the patient information sheet and informed consent form as necessary. If revisions are made, the Certified Committee for Regenerative Medicine will advise on those revisions prior to use.

14.2.3 Explaining about the research and obtaining informed consent from subjects

Before subject enrollment, the principal investigator or subinvestigator will obtain written informed consent from candidate patients after explaining about the clinical research in simple language by using the patient information sheet.

The person who provided the explanation and the subject will each sign and date the informed consent form. The original informed consent form will be kept at the research site, and a copy will be given to the subject. If the subject is unable to sign the informed consent form due to visual impairment, a designated representative will sign the form on the subject’s behalf. If the subject is unable to personally read the patient information sheet due to visual impairment, a witness will sign the witness section of the informed consent form. The designated representative or witness must be a person who is not involved in the conduct of this research, to ensure that they will not be unduly influenced by researchers and other staff.

If the patient information sheet and informed consent form are revised, investigators will provide an explanation again to subjects currently participating in the research using the revised documents and obtain written consent again. However, this does not apply to subjects whose period of participation in the research has ended.

14.3 Protection of Personal Information

After a subject's consent is obtained, all handling of cases, including data management and manufacturing management, will be managed using a linkable anonymized subject identification code or registration number. The key for matching the anonymized code to the subject's name and the informed consent form with the subject's name should be stored securely in a lockable document storage room. The data obtained from this research will also be registered and utilized in the National Regenerative Medicine Database (NRMD/CR) maintained by the Japanese Society for Regenerative Medicine, with personally identifying information removed.

When results are published, the subjects' personal information will be protected by means such as ensuring that subject names are not directly published. Records and other information pertaining to this research that are stored at the research site will be disclosed when requested by auditors and regulatory authorities, but confidentiality will be maintained.

14.4 System for Handling Complaints and Inquiries from Subjects and Other Relevant Parties

Complaints and inquiries from subjects or other relevant parties will be handled by the principal investigator and, if necessary, the investigator acting as the subject's physician. In addition, the research help desk at the research site will also provide support for inquiries during long-term follow-up. Contact information will be included in the patient information sheet.

14.5 Compensation for Health Injury

In the event that a subject suffers any health injuries as a result of this research, the site will provide medical treatment and take any other necessary measures.

For this clinical research, all persons involved in the research, including the principal investigator, will enroll in clinical research insurance that will cover compensation for subjects who suffer any health injury for which a causal relationship to the research cannot be denied. This insurance will pay compensation to subjects or their surviving family in the event of a clinical research-related health injury (death or disability equivalent to grade 1 or 2 under the Relief System for Sufferers from Adverse Drug Reactions). It will also pay medical expenses and allowances when subjects receive treatment due to a clinical research-related health injury (adverse event that requires at least hospitalization).

14.6 Handling of Cases When Significant Findings Regarding a Subject's Health or Inheritable Genetic Characteristics Are Obtained During the Course of the Research

If significant findings regarding a subject's health or inheritable genetic characteristics are obtained during screening or during the research period, the investigator will communicate

the relevant research results (including incidental findings) to that subject and provide appropriate medical care.

15. Changes to the Regenerative Medicine Provision Plan

15.1 Non-minor Changes to the Regenerative Medicine Provision Plan

If the principal investigator intends to change the Regenerative Medicine Provision Plan, he/she will prepare Form 2 "Notification of Changes to Regenerative Medicine Provision Plan Items," the plan to be changed, and other documents specified in Article 27, Paragraph 8 of the Order for Enforcement of the Act on the Safety of Regenerative Medicine (hereinafter, "the Enforcement Order"). The head of the research site will ask the Certified Committee for Regenerative Medicine named in the plan in advance for its opinion on whether the plan conforms to the standards for the provision of regenerative medicine. When the head of the research site receives an opinion from the Certified Committee for Regenerative Medicine, the head of the site will report changes made to the Regenerative Medicine Provision Plan and other measures taken based on that opinion back to the Certified Committee for Regenerative Medicine. The head of the research site will submit Form 2, the written opinion of the Certified Committee for Regenerative Medicine named in the plan, the revised plan, and other documents specified in Article 27, Paragraph 8 of the Enforcement Order to the Minister of Health, Labour and Welfare in advance. The ethical, scientific, and medical validity of the changes shall be thoroughly considered.

15.2 Minor Changes to the Regenerative Medicine Provision Plan

If making a minor change, the principal investigator will prepare Form 3, "Notification of Minor Changes to Regenerative Medicine Provision Plan Items." The head of the research site will notify the Certified Committee for Regenerative Medicine of the minor change within 10 days of the change date and will also notify the Minister of Health, Labour and Welfare of the change using Form 3.

15.3 Changes to the Regenerative Medicine Provision Plan That Do Not Require Notification

The principal investigator will record any changes made that do not require notification.

16. Periodic Reports

16.1 Periodic Reports Regarding the Regenerative Medicine Provision Plan

16.1.1 Reporting to the Certified Committee for Regenerative Medicine

The principal investigator will submit to the head of the research site a report regarding items 1) to 5) below concerning the status of provision of regenerative medicine using Appendix Form 3, "Periodic Report on Provision of Regenerative Medicine (for Committee Report)." The report is to be submitted annually starting one year after the date of submission of the

Regenerative Medicine Provision Plan to the Minister of Health, Labour and Welfare, immediately after the expiration of the applicable period. The head of the research site will then report to the Certified Committee for Regenerative Medicine regarding that report.

When making this report, the documents listed in each item of Article 27, Paragraph 8 of the Enforcement Order (only those for which the Certified Committee for Regenerative Medicine does not have the latest version) must be attached.

1) Number of people who have received the regenerative medicine product
2) Status and course of diseases or the like related to the regenerative medicine product
3) Evaluation of the safety and scientific validity of the regenerative medicine product
4) Matters concerning involvement as stipulated in each item of Article 8-8, Paragraph 1 the Enforcement Order regarding the regenerative medicine product
5) Status and subsequent handling of noncompliance with the Enforcement Order pertaining to the regenerative medicine product or to the Regenerative Medicine Provision Plan

The head of the research site and the principal investigator will take necessary measures based on the written opinion issued by the Certified Committee for Regenerative Medicine in response to this report and will report the details of those measures to the Certified Committee for Regenerative Medicine.

16.1.2 Reporting to the Minister of Health, Labour and Welfare

The head of the research site will report the items listed in 1) of 16.1.1 and other necessary items concerning the status of regenerative medicine provision to the Minister of Health, Labour and Welfare using Appendix Form 4, “Periodic Report on Provision of Regenerative Medicine (for MHLW Report).” The report is to be submitted annually starting one year after the date of submission of the Regenerative Medicine Provision Plan to the Minister of Health, Labour and Welfare, within 90 days of the expiration of the applicable period.

When making this report, the opinion issued by the Certified Committee for Regenerative Medicine regarding the report described in 16.1.1 and the documents listed in each item of Article 27, Paragraph 8 of the Enforcement Order (only those for which the Minister of Health, Labour and Welfare does not have the latest version) must be attached.

16.2 Reporting on Safety Information

“Safety information” refers to facts such as new scientific findings or safety measures taken by Japanese and overseas regulatory authorities after the start of the research that could change the burden on subjects or the overall evaluation of expected risks and benefits.

Investigators will report to the principal investigator if any safety information is obtained. If the principal investigator receives a report on safety information that may affect the continuation of this clinical research, he/she will report it to the head of the research site using Reference Form 2 “Safety Information Report” and discontinue or suspend the research or change the Regenerative Medicine Provision Plan as necessary.

16.3 Reporting of Noncompliance

"Noncompliance" refers to noncompliance with the Enforcement Order, Regenerative Medicine Provision Plan, and clinical protocol, including deviations and falsification or fabrication of research data. In addition, "particularly serious noncompliance (hereinafter, "serious noncompliance") refers to noncompliance that affects subjects' human rights or safety or the reliability of results.

Investigators will report any noncompliance they discover to the principal investigator. The principal investigator will report to the head of the research site using Reference Form 3 "Noncompliance Report" for non-serious cases of noncompliance and Appendix Form 10 "Serious Noncompliance Report" for serious cases of noncompliance. The head of the research site will report serious cases of noncompliance to the Certified Committee for Regenerative Medicine for review.

The head of the research site and the principal investigator will take necessary measures based on the written opinion issued by the Certified Committee for Regenerative Medicine and will report details of those measures to the Certified Committee for Regenerative Medicine.

Investigators will record all cases of noncompliance, regardless of the reason for noncompliance.

16.4 End-of-study Report

Appendix Form 9 "Synopsis of Clinical Study Report" shall be submitted to the Minister of Health, Labour and Welfare within one month from the date that the Certified Committee for Regenerative Medicine issues its opinion on the CSR and CSR synopsis for this research. The date when the CSR synopsis is published in the Japan Registry of Clinical Trials (jRCT) will be considered the date of completion of this research. However, if results are to be published in a journal article or similar medium, the timing of publication in jRCT may be set after publication in that journal or similar medium after reporting the intent to publish to the Certified Committee for Regenerative Medicine. In such a case, the CSR synopsis should be submitted to the Minister of Health, Labour and Welfare by the due date, and the planned timing for publication in jRCT should be indicated at the time of submission.

16.5 Reporting of Discontinuation

16.5.1 Report from principal investigator to head of research site

When provision of the regenerative medicine product is discontinued, the principal investigator will immediately inform the head of the research site.

16.5.2 Notification from the head of the research site to the Certified Committee for Regenerative Medicine and submission to the Minister of Health, Labour and Welfare

When the head of the research site discontinues the entire clinical research, including when he/she receives a discontinuation report from the principal investigator as described in 16.5.1,

the head of the research site will notify the Certified Committee for Regenerative Medicine of the discontinuation within 10 days of the discontinuation date and will notify the Minister of Health, Labour and Welfare using Form 4 "Notification of Discontinuation of Regenerative Medicine Provision."

17. Clinical Study Report

The principal investigator will prepare a CSR and CSR synopsis and will submit these to the head of the research site. The deadline for submission will generally be within one year from the date of completion of data collection for all research endpoints. The head of the research site will report the information in these documents to the Certified Committee for Regenerative Medicine.

18. Storage of Samples and Records

18.1 Recording and Storage of Records Related to the Regenerative Medicine

The head of the research site will, as a rule, delegate the storage of records related to the regenerative medicine to the principal investigator.

18.1.1 Records to be retained for 30 years

The following items related to the regenerative medicine will be recorded for each subject.

1) Subject's address, name, sex, and date of birth
2) Diagnosis and main symptoms
3) Type of specific processed cell product used, route of administration, and other details regarding the regenerative medicine, as well as evaluations
4) Information on cells used in the regenerative medicine
5) If the production of the specific processed cell product is outsourced, the contracted party and details of outsourced tasks
6) Date of provision of regenerative medicine
7) Name of physician who provided the regenerative medicine
8) Other items necessary to provide the regenerative medicine

	Locations of records
1), 2), 3), 6), 7), 8)	Records will be maintained at the Ophthalmology, Department of Ophthalmology, Graduate School of Medicine of Osaka University and the Department of Ophthalmology at Osaka University Hospital.
4)	Records will be maintained at the following three facilities.<br>(1) CiRA Foundation (formerly the Facility for iPS Cell Therapy [FiT])<br>(2) Center for Gene and Cell Processing, Takara Bio Inc.<br>(3) Medical Center for Translational Research Cell Processing Center

	(MTR-CPC)
5)	For the following two facilities, records will be kept at the Ophthalmology, Department of Ophthalmology, Graduate School of Medicine of Osaka University.<br>(1) Facility for iPS Cell Therapy (FiT)<br>(2) Center for Gene and Cell Processing, Takara Bio Inc.

The above records, together with the documents specified in Article 34, Paragraph 3 of the Enforcement Order, will be retained for 30 years from the date of discontinuation or completion of the clinical research. After the retention period, records will be destroyed appropriately in accordance with the disposal guidelines of Osaka University Graduate School of Medicine and Osaka University Hospital.

18.1.2 Records to be retained for 5 years

The following items related to the regenerative medicine will be recorded for each subject.

1) Items identifying subjects (subject screening roster)
2) Items related to medical treatment and examination of subjects
3) Items related to research participation (informed consent forms)
4) Other items necessary to provide the regenerative medicine as an investigative therapy

The above records, together with the documents specified in Article 34, Paragraph 4 of the Enforcement Order, will be retained at the Ophthalmology, Department of Ophthalmology, Graduate School of Medicine of Osaka University and the Department of Ophthalmology at Osaka University Hospital for 5 years from the date of discontinuation or completion of the clinical research. If a record is corrected, the name of the corrector and the date the correction was made must be recorded and retained with the corrected record. After the retention period, records will be destroyed appropriately in accordance with the disposal guidelines of Osaka University Graduate School of Medicine and Osaka University Hospital.

18.2 Storage of Samples

The head of the research site will, as a rule, delegate the storage of samples to the principal investigator.

The following samples for each research subject will be stored at the research site for 10 years in order to investigate the cause of any infectious disease that may develop in a subject. After the retention period, samples will be destroyed appropriately in accordance with the disposal guidelines of Osaka University Hospital.

1) Blood samples
2) Reserve samples of the specific processed cell product (cell suspensions for cryopreservation will be prepared from multiple shipments of untransplanted iCEPS, including reserve stock)

19. Direct Access to Source Documents

The principal investigator shall accept and cooperate with monitoring and auditing by monitoring and auditing officers appointed by the principal investigator and investigations by the Certified Committee for Regenerative Medicine and regulatory authorities. In such a case, direct access shall be given for all research-related records, such as the source documents.

The source documents are the records necessary for reproducing and evaluating the factual course of the clinical research, such as medical records, informed consent forms, various test data, documents related to the specific processed cell product and its management, and data from which CRFs are prepared.

20. Monitoring and Audits

20.1 Monitoring

Monitors will consider the following and conduct monitoring in accordance with the "Procedures for Monitoring."

1) That subjects' human rights are protected and their safety is ensured
2) That the research is being conducted in compliance with the latest clinical protocol as well as laws and regulations
3) That written consent to the research has been obtained from subjects
4) That records can be verified as accurate by comparison with source documents

20.2 Audits

For purposes of assuring the quality of the clinical research, the principal investigator will engage an auditor who is independent and separate of routine monitoring and research quality control tasks to carry out audits.

The auditor will conduct audits in accordance with the "Procedure for Conducting Audits" at the research site.

21. Sources of Funding for the Clinical Research and Conflicts of Interest

This clinical research will be conducted with research funds from Osaka University (research funds granted by the Japan Agency for Medical Research and Development).

It should also be noted that in the future, the "allogeneic iPS cell-derived corneal epithelial cell sheets" used in this research may be commercialized by Raymei Inc., a venture company launched by researchers at Osaka University. The principal investigator owns stock in and serves on the board of Raymei Inc. In addition, Osaka University holds patent rights for "allogeneic iPS cell-derived corneal epithelial cell sheets," and inventors on that patent are among the parties involved in conducting this research (the principal investigator and some of the personnel involved in the production of iCEPS).

Conflicts of interest pertaining to the principal investigator, subinvestigators, and other relevant parties will be managed appropriately by the principal investigator, with the head of the research site assuming responsibility.

22. Burden of Research-related Expenses

Expenses related to this clinical research will be borne by the principal investigator.

23. Registration of the Clinical Research and Attribution and Publication of Results

23.1 Registration of the Clinical Research

This research is registered in the University Hospital Medical Information Network Clinical Trials Registry (UMIN-CTR) and the Japan Registry for Clinical Trials (jRCT) organized by the Ministry of Health, Labour and Welfare, and a research synopsis and information on the progress and results will be published in these registries.

23.2 Attribution and Publication of Results

Intellectual property rights arising from this clinical research shall belong to Osaka University and the researchers conducting this research.

When presenting part or all of the results of this clinical research for academic purposes, or when submitting to a specialized medical journal, etc., the principal investigator will decide whether it is appropriate.

When making public, the names of the subjects will not be directly disclosed, and the protection of personal information shall be given due consideration.

24. Administrative Structure of the Research

As described in Appendix 1

25. References

1) Thoft RA, Friend J. The X, Y, Z hypothesis of corneal epithelial maintenance. Invest Ophthalmol Vis Sci. 1983; 24:1442-3.

2) Cotsarelis G, Cheng SZ, Dong G, Sun TT, Lavker RM. Existence of slow-cycling limbal epithelial basal cells that can be preferentially stimulated to proliferate: implications on epithelial stem cells. Cell. 1989; 57:201-9.

3) Nishida K. Tissue engineering of the cornea. Cornea. 2003; 22:S28-34.

4) Nishida K, Watanabe H: 4. Corneal epithelial transplantation. In: Corneal Clinic, 2nd ed. (Manabe R, Kinoshita S, Ohashi Y, supervising eds.). Igaku-Shoin, Tokyo, pp.261-263 (2003)

5) Ilari L, Daya SM. Long-term outcomes of keratolimbal allograft for the treatment of severe ocular surface disorders. Ophthalmology. 2002; 109:1278-84.

6) Tsubota K, Satake Y, Kaido M, Shinozaki N, Shimmura S, Bissen-Miyajima H, et al. Treatment of severe ocular-surface disorders with corneal epithelial stem-cell transplantation. N Engl J Med. 1999; 340:1697-703.

7) Liang L, Sheha H, Tseng SC. Long-term outcomes of keratolimbal allograft for total limbal stem cell deficiency using combined immunosuppressive agents and correction of ocular surface deficits. Arch Ophthalmol. 2009; 127:1428-34.

8) Han ES, Wee WR, Lee JH, Kim MK. Long-term outcome and prognostic factor analysis for keratolimbal allografts. Graefes Arch Clin Exp Ophthalmol. 2011; 249:1697-704.

9) Dua HS, Azuara-Blanco A. Autologous limbal transplantation in patients with unilateral corneal stem cell deficiency. Br J Ophthalmol. 2000; 84:273-8.

10) Kenyon KR, Tseng SC. Limbal autograft transplantation for ocular surface disorders. Ophthalmology. 1989; 96:709-22.

11) Liang L, Sheha H, Li J, Tseng SC. Limbal stem cell transplantation: new progresses and challenges. Eye (Lond). 2009; 23:1946-53.

12) Kheirkhah A, Raju VK, Tseng SC. Minimal conjunctival limbal autograft for total limbal stem cell deficiency. Cornea. 2008; 27:730-3.

13) Chen JJ, Tseng SC. Corneal epithelial wound healing in partial limbal deficiency. Invest Ophthalmol Vis Sci. 1990; 31:1301-14.

14) Rama P, Matuska S, Paganoni G, Spinelli A, De Luca M, Pellegrini G. Limbal stem-cell therapy and long-term corneal regeneration. N Engl J Med. 2010; 363:147-55.

15) Nishida K, Yamato M, Hayashida Y, Watanabe K, Yamamoto K, Adachi E, et al. Corneal reconstruction with tissue-engineered cell sheets composed of autologous oral mucosal epithelium. N Engl J Med. 2004; 351:1187-96.

16) Clinical Study on Cultivated Autologous Oral Mucosal Epithelial Sheet Transplantation for Treating Patients with Intractable Keratoconjunctival Disease. Clinical Research Report for Future Medicine P0402 ID: UMIN000012819

17) Nakamura T, Takeda K, Inatomi T, Sotozono C, Kinoshita S. Long-term results of autologous cultivated oral mucosal epithelial transplantation in the scar phase of severe ocular surface disorders. Br J Ophthalmol. 2011; 95:942-6.

18) Dua HS, Saini JS, Azuara-Blanco A, Gupta P. Limbal stem cell deficiency: concept, aetiology, clinical presentation, diagnosis and management. Indian J Ophthalmol. 2000; 48:83-92.

19) Burillon C, Huot L, Justin V, Nataf S, Chapuis F, Decullier E, et al. Cultured autologous oral mucosal epithelial cell sheet (CAOMECS) transplantation for the treatment of corneal limbal epithelial stem cell deficiency. Invest Ophthalmol Vis Sci. 2012; 53:1325-31.

20) Hayashi R, Ishikawa Y, Sasamoto Y, Katori R, Nomura N, Ichikawa T, et al. Co-ordinated ocular development from human iPS cells and recovery of corneal function. Nature. 2016; 376-80.

21) Fernandes M, Sangwan VS, Rao SK, Basti S, Sridhar MS, Bansal AK, et al. Limbal stem cell transplantation. Indian J Ophthalmol. 2004; 52:5-22.

22) Bakhtiari P, Djalilian A. Update on limbal stem cell transplantation. Middle East Afr J Ophthalmol.2010; 17:9-14.

23) Shimizu Y: Diabetic eye disease. Diabetic retinopathy. J Nippon Med Sch 51(6): 110-113 (1984)

24) JCOG website: Common Terminology Criteria for Adverse Events (CTCAE) version 4.0
URL: http://www.jcog.jp/doctor/tool/ctcaev4.html
